# Mechanosensory encoding of surface mechanics optimizes locomotion

**DOI:** 10.1038/s41467-026-75352-7

**Published:** 2026-07-25

**Authors:** Aleksandra Pidde, Montserrat Porta-de-la-Riva, Costanza Agazzi, Carmen Martínez-Fernández, Alice Lorrach, Ashutosh Bijalwan, Neus Sanfeliu-Cerdán, Alba Calatayud-Sanchez, Eric Torralba-Sales, Ravi Das, José J. Muñoz, Michael Krieg

**Affiliations:** 1 ICFO - Institut de Ciéncies Fotóniques, Castelldefels, The Barcelona Institute of Science and Technology, Barcelona, Spain; 2 Centre Internacional de Métodes Numérics en Enginyeria (CIMNE), Barcelona, Spain; 3 Laboratori de Cálcul Numéric (LaCáN), Department of Mathematics, Escola d, Universitat Politécnica de Catalunya (UPC), Barcelona, Spain; 4 Institut de Matemátiques de la UPC- BarcelonaTech (IMTech), Barcelona, Spain

**Keywords:** Decision, Biomedical engineering, Optical imaging, Biological physics, Touch receptors

## Abstract

Locomotion - whether walking, running, or crawling - depends on the precise coordination of forces between the body and its surroundings. Two critical factors in this process are the force that resists the relative motion between two bodies, and mechanosensation, the body’s ability to sense and respond to mechanical forces. Together, these processes enable efficient locomotion across diverse environments, yet how body-environment interaction forces shape neural activity in freely moving animals remains poorly understood. Here we show that the ‘gentle touch’ receptor neurons (TRNs) in the *Caenorhabditis elegans* body wall are sensitive to dynamic surface mechanics. Using a combination of calcium imaging and traction force microscopy in freely behaving animals informed by micromechanical stimulation, connectome-scale simulations, and optimal control theory, we identify friction force and crawling velocity as key determinants of mechanoreceptor activity. Mutations disrupting MEC-4 DEG/ENaC ion channel activity in body wall mechanoreceptors produce lethargic animals with impaired proprioceptive regulation, suggesting functional coupling between surface mechanoreceptors and proprioceptors. Together, our findings reveal that gentle ‘touch’ receptors encode body-environment interaction forces during movement to optimize locomotor energetics.

## Abstract (web summary)

Locomotion emerges from a constant dialogue between the body and the physical environment, requiring both frictional interactions and mechanosensation to coordinate movement. By combining experiments and theory, the authors show that classical gentle-touch receptor neurons in *C. elegans* sense dynamic surface traction during crawling, uncovering a previously unrecognized role for skin mechanoreceptors in proprioceptive locomotion control.

## Introduction

Mechanosensation allows us to explore the immediate physical environment^1^. In addition to conscious touch, mechanosensation plays a key role in communication, bonding, and social comfort^2,3^, and provides essential mechanical information about the surface when we walk^4^. This feedback mechanism is crucial for adaptive locomotion and proprioception, enabling individuals to respond to uneven terrain and varying support surfaces^5,6^. Although they play a critical role in the somatosensory system, the cellular and mechanical mechanisms of how animals process plantar feedback and which mechanical stimuli activate these receptors remain understudied and therefore poorly understood.

**Fig. 1:**
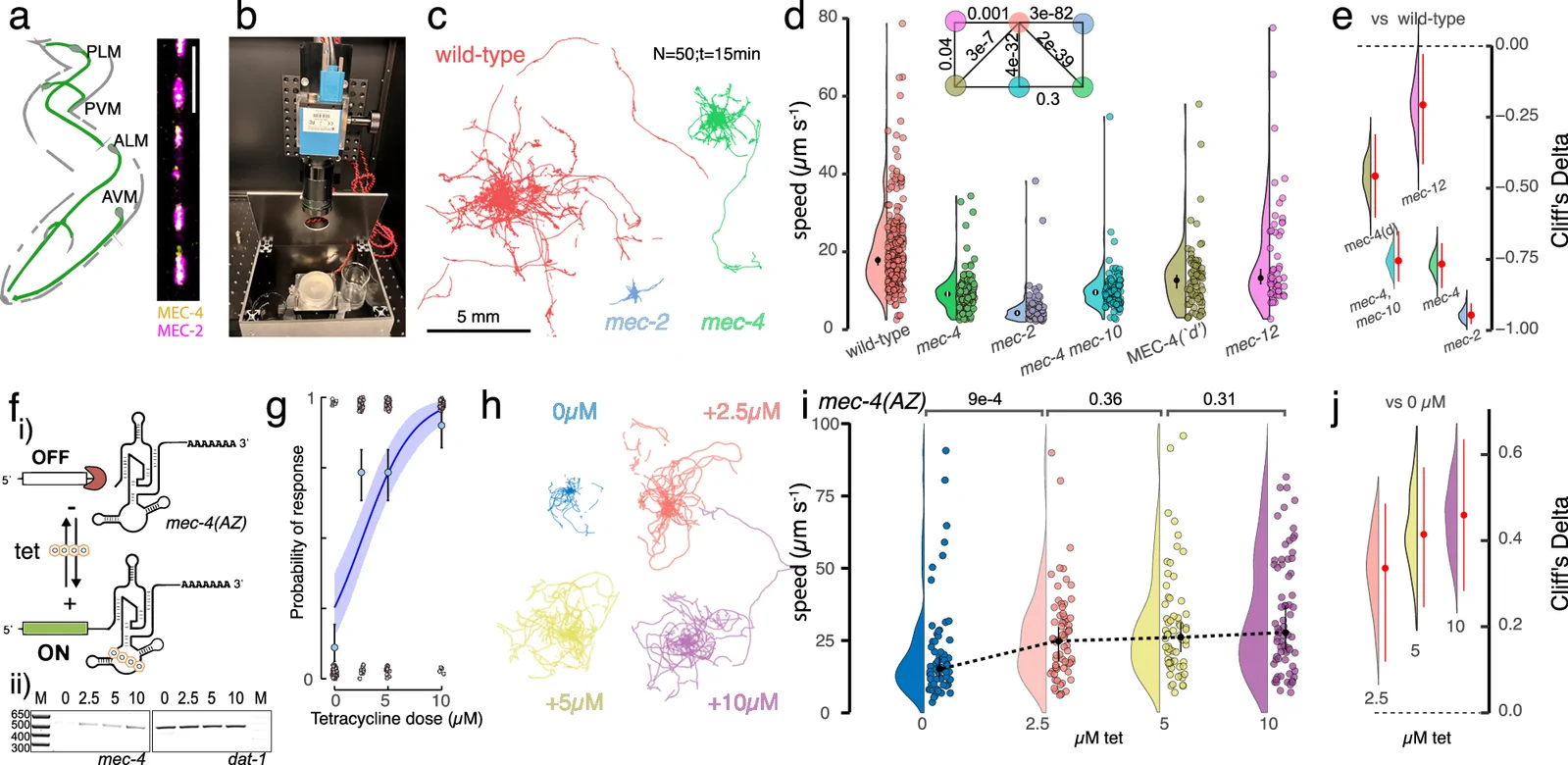
Body-wall mechanoreceptor function promotes locomotion in *C. elegans.* **a** Schematic of the worm with the location of the body wall mechanoreceptors shown in green (left) and a representative micrograph of MEC-2/MEC-4 colocalization in the ALM neurite (right). Scale bar = 5μm, representative for *N* = 20 investigated animals. **b** Photograph of the light and vibration-isolated small animal behavior tracking platform. **c** Trajectories of 15 min locomotion behavior for wild-type animals, *mec-2*, and *mec-4* mutants. *N* = 50 animals per condition on NGM plates supplemented with food. **d** Distribution of the crawling speed from wild-type and various *mec* mutant genes visualized as a violin plot. *MEC-4(‘d’)* indicates the degenerin mutation leading to loss of TRNs in adult animals (*e1611*, A713V,^86^). Black solid circles show medians, range indicates 95% confidence interval. For the exact number of measurements and replicates, consult Supplementary Table 3. Inset: Two-sided Kolmogorov–Smirnov (KS) test *p*-values comparing each pair of distributions (numbers inside the matrix). Colors correspond to genotype groups consistently across violins, estimation plots, and the KS matrix. **e** Estimation plots of bootstrapped Cliff’s Delta (red points indicate median, red bars show 95% bias-corrected confidence intervals) for each comparison relative to the reference genotype. A dashed horizontal line at zero indicates no effect. **f** (i) Schematic of the aptazyme (AZ), and the acute rescue experiment using tetracycline to block it. Adapted from ref. ^87^, under a Creative Commons Attribution 4.0 International (CC BY 4.0) license (https://creativecommons.org/licenses/by/4.0/). Colors and labels were modified. (ii) Representative RT-PCR result from *mec-4(AZ)* animals exposed to increasing tet concentration, detecting *mec-4* and *dat-1* mRNA as a control. Representative for *N* = 4 independent experiments. **g** Representative results of a touch assay in animals expressing *mec-4(AZ)* at increasing tetracycline concentrations. The probability of responding in the 10-touch assay increased with dose (log-odds = 0.416  ± 0.066 per μM, *z* = 6.34, *p* = 2.3 × 10^−10^), with notable animal-to-animal variability (random intercept SD = 0.89). Points show individual trial outcomes (0 = no response, 1 = response). The solid line represents the predicted probability from a mixed-effects binary logistic regression with tetracycline dose as a fixed effect and animals as random intercepts, and the shaded area indicates the 95% confidence interval. Blue circle and range indicate mean ± SD. **h** Trajectories acquired for 15 min from 30 animals under each condition analyzed for *mec-4(AZ)* and the indicated concentrations of tetracycline. **i** Violin plot of crawling speed in *mec-4(AZ)* animals under the stated tetracycline concentrations. Black circles show medians, range indicates 95% confidence interval. *P*-values comparing each pair of distributions calculated from a two-sided KS-test. See Supplementary Table 3 number of replicas and data points. **j** Estimation plots of bootstrapped Cliff’s Delta (red points indicate median, red bars show 95% bias-corrected confidence intervals) for each comparison relative to the reference genotype. A dashed horizontal line at zero indicates no effect.

*Caenorhabditis (C.) elegans* senses mechanical forces delivered to its body wall with a set of different mechanoreceptors^1,7^. Depending on the stimulus magnitude, these mechanoreceptors have been coined harsh and gentle touch receptors. The six neurons responsible for sensing gentle mechanical stimuli are called PVM, PLM(L/R), ALM(L/R), AVM, and tile the body in anterior and posterior receptive fields (Fig. 1a)^7^. All of them express mechanosensitive ion channel MEC-4, whose association with other mec genes, such as *mec-2*, is critical for proper mechanosensitivity (Fig. 1a). However, and despite being mechanosensitive^8^, laser ablation of PVM resulted in a negligible effect on the behavioral response to touch^9^, questioning its role in mechanosensation. What other stimuli, beyond touch, might TRNs have evolved to sense? Importantly, aversive mechanical stimuli lead to enhanced sensory arousal^10^ and mutations in genes that cause loss of sensory function cause a striking lethargic phenotype^9,11,12^. Likewise, loss of the pore-forming subunit of the mechanoelectrical transduction channel MEC-4^13,14^, a member of the Degenerin/Epithelial sodium channel/acid-sensing ion channel (DEG/ENaC/ASiC) family, leads to changes in the generation of locomotor patterns, suggesting that TRNs induce a behavioral response to a changing physical environment^15,16^. Lastly, it was also shown that TRNs, through MEC-4 ion channels, sense mechanical shear stresses^17^, exhibiting higher activity when stimuli are delivered at a higher rate^8,18^, similar to human mechanoreceptors involved in touch^19^. Together, this may hint towards a role of these neurons in locomotion, through sensing dynamic mechanical properties and body-substrate interaction forces.

Here, we synthesize this large body of previous work with new evidence that gentle body wall mechanoreceptors sense friction with the surrounding environment and use MEC-4 as a velocity-dependent force sensor. In particular, we define a role for PVM in sensing substrate compliance, a neuron that specifically activates in response to navigation in a soft environment. Using traction force microscopy, we map the forces that are exerted by the animal on the substrate and show that the activity after direct stimulation of PVM is reflected in different proprioceptive circuits, in a *mec-4*-dependent manner. Based on these data and mathematical simulations, we propose that the gentle body wall mechanoreceptors provide essential information about substrate mechanics, crosstalk with proprioceptive neurons, akin to mammalian plantar mechanoreceptors.

## Results

### Loss of body-wall mechanoreception leads to lethargic animals

In addition to their failure to respond to external touch to the body wall, *mec-4* mutants are known to be lethargic^9^. To corroborate and extend these observations, we first recorded 15 min long videos in an isolated chamber, protecting animals from external light, vibrations, and variations of temperature and humidity (Fig. 1b, see the “Methods” section) to understand whether the loss of mechanosensitivity to touch is correlated to a loss of locomotor activity. We tracked individual animals lacking TRNs (MEC-4(‘d’)) and various mutant alleles with loss-of-function in *mec-4*, *mec-10*, *mec-2* and *mec-12*. Although some of these genes are expressed in other neurons than TRNs^20^, they all functionally intersect in TRNs and share a common role in mechanotransduction. Their loss of function causes a strong, almost complete absence of touch sensitivity and touch-induced currents^14^. We found that hermaphrodite mutants of all alleles are significantly less mobile than wild-type animals of similar age on food (Fig. 1c–e, Supplementary Movie 1), affecting both forward and backward locomotion (Supplementary Fig. 1a). As previously described in ref. ^9^, this difference is strongly reduced off food, whereas *mec-4* and *mec-2* mutant males retain their mobile activity on food (Supplementary Fig. 1b and ref. ^9^). Moreover, locomotor defects can be induced either by ectopic expression of a miniSOG transgene^21^ to ablate individual TRNs upon blue-light illumination (Supplementary Fig. 1c) or by blocking synaptic transmission through TRN-specific expression of tetanus toxin (TeTx, Supplementary Fig. 1d), suggesting that vesicular neurotransmission underlies TRN-mediated locomotion phenotypes. Because animals expressing miniSOG in the dark displayed defects already independent of light, we sought to determine whether the effect arises from the chronic absence of mechanoreception or can be induced acutely. To that end, we expressed the anion channelrhodopsin ACR1^22^ in TRNs using the UAS/Gal4 system^23^. Upon 519nm, but not 630nm, illumination, ACR1 activity transiently hyperpolarized TRNs and reliably slowed locomotion and produced shorter trajectories (Supplementary Fig. 1e), demonstrating that acute reversible silencing of mechanoreceptor neurons is sufficient to elicit a lethargus-like state (Supplementary Fig. 1f). Controls, however, which were not supplemented with ATR or did not express ACR1 in absence of TRN:Gal4 driver did not show a significant difference between green and red light stimulation (Supplementary Fig. 1f). As described previously^24–26^, the lethargic *mec* mutant phenotypes are not caused by defects in muscle function or general paralysis because all mutants retain normal locomotion on standard NGM agar, with no gross differences from wild-type when stimulated with cues other than gentle touch. Thus, our data suggest that mechanosensitivity in TRNs is linked to spontaneous locomotor behavior.

We then asked if MEC-4-induced neuronal activity patterns can induce changes in synaptic plasticity and whether their absence causes lethargic phenotypes. Previously, a repetitive optogenetic stimulus administered to channelrhodopsin 2-expressing (ChR2) motor neurons in the midbody was shown to induce the bending motion of the head to a new frequency^27,28^. TRNs do not display rhythmic activity patterns but occasional stochastic calcium spikes in their sensory dendrite in immobilized wild-type animals (Supplementary Fig. 2a). Thus, we sought to emulate a stochastic activity pattern in animals expressing the blue light-gated ion channel ChR2 in TRNs^26^. When applying a random optogenetic stimulus to *mec-4* mutant animals for 48 h (Supplementary Fig. 2b), we were unable to overcome the lethargic phenotype associated with it (Supplementary Fig. 2c) suggesting that the entrainment protocols used here are insufficient to explain plasticity-related phenotypes in the absence of MEC-4. However, we cannot rule out the possibility that MEC-4-dependent plasticity requires longer or developmentally timed stimulation protocols.

To provide direct evidence that MEC-4 function enhances agile locomotion, we engineered an aptamer ribozyme^29^ in the 3’UTR of the endogenous *mec-4* locus that leads to steady and efficient degradation of its mRNA (Fig. 1f). Indeed, the introduction of the ribozyme led to the disruption of MEC-4 protein, as visualized by the absence of fluorescent signal of a mScarlet::MEC-4 fusion (see Methods and below). Consequently, these animals do not respond to external touch (Fig. 1g) and are lethargic (Fig. 1h–j). Importantly, the ribozyme’s function to cleave itself can be disabled upon the addition of low concentrations of tetracycline, and the addition of these concentrations of tetracycline itself had little effect on locomotion velocity in wild-type animals (Supplementary Fig. 3a), supporting the specificity of the aptazyme-mediated rescue. Using RT-PCR, we verified that the addition of tetracycline rescued *mec-4* mRNA expression (Fig. 1f, Supplementary Fig. 3b–d) and assayed these animals in a classical touch assay. Indeed, animals responded to external touch after tetracycline addition (Fig. 1g), and their increase in mechanosensitivity was accompanied by an increase of MEC-4 protein expression (Supplementary Fig. 3d), coinciding with robust rescue of the lethargic locomotion phenotype (Fig. 1h–j). Thus, this acute rescue effect from its endogenous locus strongly supports the causal role of MEC-4 mechanosensitivity in locomotion. Consequently, we hypothesize that TRNs are mechanosensors on the body wall involved in detecting the mechanical load of the environment to induce animal locomotion, similar to plantar mechanoreceptors in mammals.

### External mechanoreception adjusts proprioceptive feedback

**Fig. 2:**
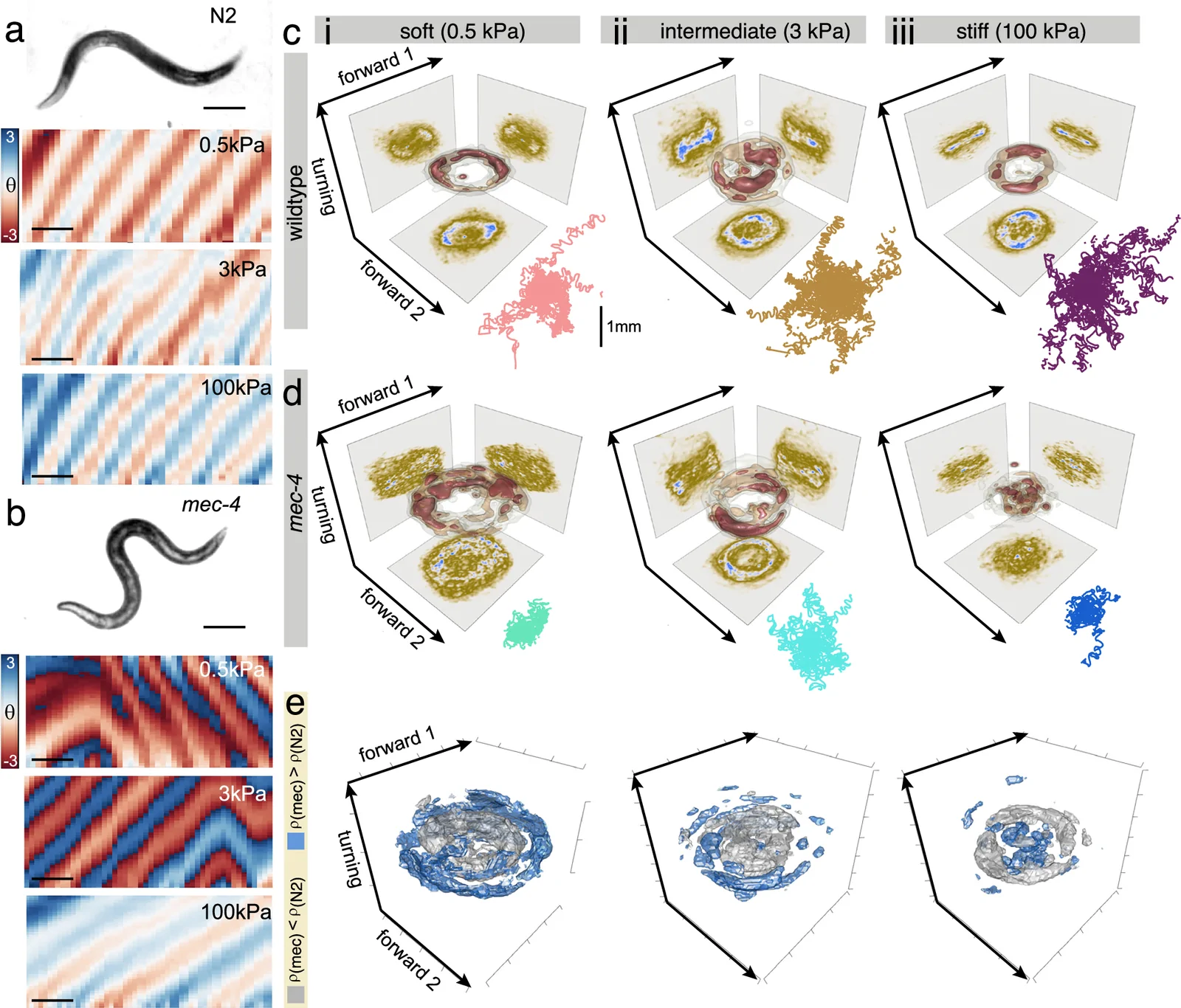
Animals adjust their gait on soft substrates in a MEC-4-dependent manner. Representative photograph of a wild-type (**a**) and *mec-4* mutant (**b**) animal crawling on soft substrates (0.5 kPa, scale bar = 100 μm). Representative curvature kymographs for the three different elastic matrices tested are shown below. Scale bar = 2 s. Locomotion of **c** wild-type and **d** *mec-4* mutant animals on (i) 0.5 kPa, (ii) 3 kPa, and (iii) 100 kPa stiff polyacrylamide hydrogels. The 3D plot shows the joint probability distribution (equivalent to a discrete 3D histogram) of the two forward and turning modes in the Eigenworm subspace, indicating the distribution of postural states. Isosurfaces (*c* = 10 (red), 25 (gold), 50(gray)) indicate the highest-density regions enclosing increasing fractions of the behavioral probability distribution, highlighting frequently occupied locomotor states and transitions between them. Projection of the 3D cloud on the planes indicates the 2D probability densities along the corresponding Eigenworms. Trajectories below the 3D histogram indicate the track length of all animals recorded over a 2 min period. **e** 3D plot of the statistically significant differences comparing the joint probability distribution shown in (**c**) and (**d**), comparing control and *mec-4* mutant animals crawling on soft (0.5 kPa), intermediate (3 kPa), and stiff (100 kPa) substrates. Silver voxel indicates higher density for N2 control animal, blue voxels indicate higher density for *mec-4* mutants with *p* < 0.01. Number of analyzed animals can be consulted in Supplementary Table 3.

Previous studies have shown that animals adapt their locomotion frequency and gait in response to mechanical changes in their environment^15,30^. To understand whether MEC-4, through epression in body wall mechanosensors, contribute to the regulation of posture, we compared the crawling behavior of wild-type and *mec-4* mutant young adult animals across different polyacrylamide (PAA) hydrogels ranging in nominal stiffness from 0.5 to 100 kPa (Fig. 2a, see the “Methods” section). Because the animals did not move on PAA gels with abundant food, and the bacterial lawn interfered with high-resolution microscopy, we carried out these experiments in the absence of a food source (OP50 bacteria). We did not find a significant difference in the locomotion gait of wild-type control animals crawling on the different stiffness substrates, suggesting that wild-type animals are able to adapt to these environments (Fig. 2a, c). However, *mec-4* mutant animals crawled with significantly higher body curvature on softer gels (Fig. 2b–e, compare Supplementary Movies 2 and 3), while on substrates with a higher stiffness, animals lacking MEC-4 crawled with significantly lower curvature (Fig. 2e) and, in general, displace less compared to wild-type controls over the course of the experiment (tracks in Fig. 2). This suggests that MEC-4, using TRNs as a neuronal substrate, may sense the mechanical properties of the environment and provide input to proprioceptive neurons for adaptation of their body posture in different environments during locomotion.

**Fig. 3:**
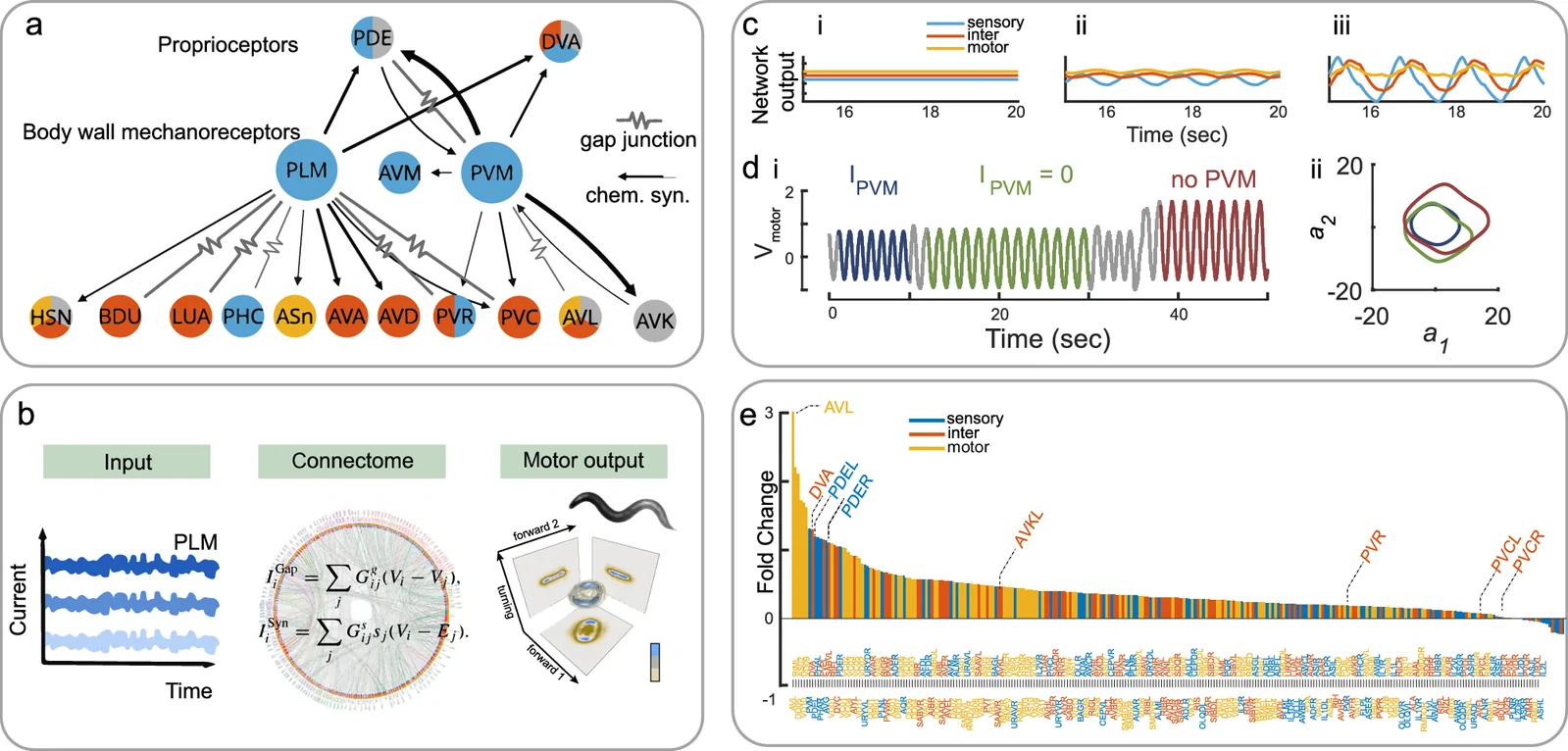
Whole neural network dynamics in silico suggest crosstalk between the mechanosensory circuit and proprioceptive neurons. **a** Wiring diagram of PVM and PLM. Color of the nodes indicates type of neuron (blue = sensory; orange = inter; yellow = motor; gray = modulatory neurons). Thickness of the edges indicates the absolute number of electrical or chemical synapses between the connected neurons. Adapted from ref. ^31^ and nemanode.org. **b** Computational pipeline to estimate the functional connectomics after constant current input (low, medium, high) into PLM. The output of the connectome is quantified as a single value decomposition of the combined motor neuron activity. **c** Average neuronal network activity in each neuron class after applying constant stimulation current with different amplitudes to PLML/R. (i) low, (ii) intermediate and (iii) high current input. Different colors indicate sensory (blue), inter (orange), and motor neurons (yellow). **d** (i) Average motor neuron voltage oscillations of the network receiving both PLM driving current plus a `mechanosensitive' PVM current (1% of the PLM driving current, dark blue), after acute removal of PVM current, resembling the *mec-4* mutation (olive green), and after PVM ablation (see Methods, dark red). (ii) Projection of the combined motor neuron output into singular vectors for all three conditions. *Note*: The pronounced increase in the limit cycle after removal of the current from PVM and PVM ablation represents the increase in the body curvature. **e** Fold-change in the amplitude of oscillations in voltage activity for all neurons following the removal of PVM input (*I*_PVM_ = 0) (as in **d** (i) olive green). Proprioceptors DVA and PDER/L are among the most severely affected.

Intriguingly, the structural connectome reveals numerous synaptic connections between the posterior touch receptor neurons PLM/PVM and the proprioceptive neurons PDE and DVA (Fig. 3a)^31^, whereas such connectivity is largely absent for the anterior touch receptors ALM and AVM (Supplementary Fig. 4a). This anatomical organization suggests a potential functional coupling between posterior TRNs and proprioceptive circuits. To obtain a systems-preview on the functional connections between the touch circuit and the proprioceptive feedback circuit, we adapted a computational model incorporating the full synaptic and gap junctions connectome (Fig. 3b). This model has previously been used to understand the influence of individual neurons on forward locomotion, using motor neuron activity to predict muscle contraction and thus behavior^32,33^. Here, we specifically asked whether loss of TRN function alters motor neuron population dynamics in silico when projected into a low-dimensional neural manifold, analogous to the Eigenworm representation of body posture^34,35^. In this model, a computationally ‘injected’ constant current to PLM is sufficient to generate stable periodic activity in motor neurons, indicating an ability to generate sustained rhythmic dynamics as an emergent property of the neural network (Fig. 3c).

We then asked how the network behaves when the activity of ALM, AVM, and PVM is silenced, mimicking the loss of *mec-4*. Removing the input from the anterior touch neurons (ALM/AVM) collapses the motor neuron oscillations, driving the system into a fixed point in the 2D subspace spanned by singular vectors (Supplementary Fig. 4b, Methods). Also, when all input connections into PLM are silenced, we observe a drastic reduction in the motor neuron population dynamics. This is consistent with the lethargic phenotype observed for the mutations affecting TRN function (Fig. 1c–e). The simulation output was markedly different when we removed the current from PVM: the amplitude of the oscillations in many neurons was increased (Fig. 3d (i)), leading to a larger limit cycle in the low-dimensional phase space (Fig. 3d (ii)). This suggests a surprising role for PVM in the regulation of body curvature.

In order to investigate the influence of the PVM neuron on the motor circuit in our simulation, we quantified the change in activity upon removal of the mechanosensitive current (emulated as 1% of the PLM driving current) from the PVM. Unexpectedly, removal of the PVM input current led to a global increase in neuronal activity. Although most neurons exhibited elevated activity levels, the largest changes were observed in the proprioceptive neurons DVA and PDE (Fig. 3e). Together, these results point toward a functional role of posterior body wall mechanoreceptors in regulating the activity of DVA and PDE proprioceptors through network-level interactions rather than purely excitatory drive. This outcome is counterintuitive given the presumed modulatory role of PVM and suggests that, despite the structural connectome indicating direct coupling, the network may operate in a regime where PVM input normally exerts a net suppressive or stabilizing influence on downstream circuits.

### Touch receptors functionally couple to mechanical proprioceptors

**Fig. 4:**
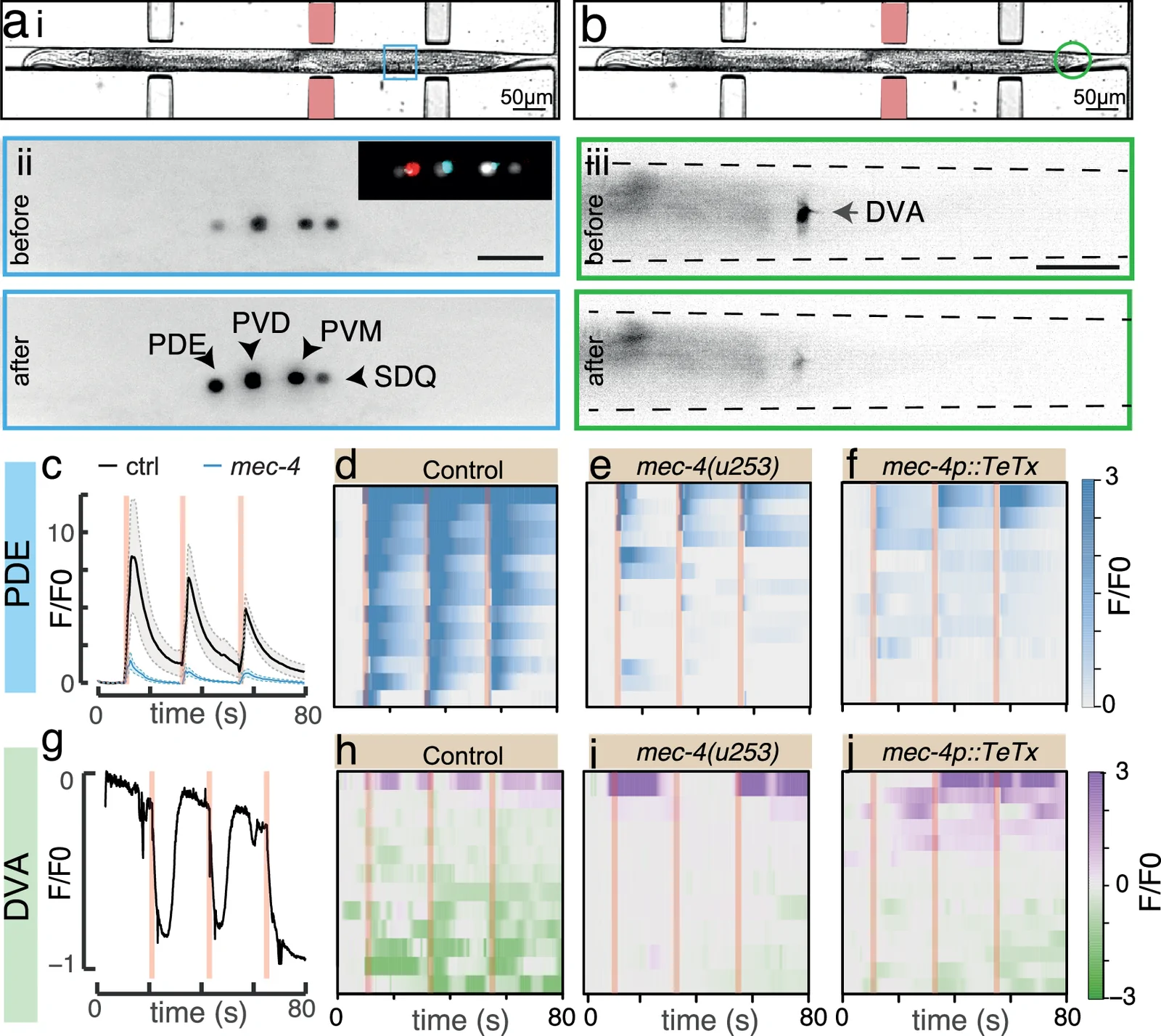
Stimulation of bodywall mechanosensors activates proprioceptive circuits. (i) Schematic of a single animal acquired at 10x magnification in the microfluidic device, highlighting the actuator and the location of **a** PDE (blue square) and **b** DVA (green circle). The actuator used for each neuron is highlighted by a red rectangle. (ii) Fluorescent micrograph with **a** PDE and **b** DVA before and after stimulation at the posterior body part. Scale bar =  10 μm. The inset in (ii) shows a snapshot of the mNeptune2.5 and mTagBFP2 fluorescent tags of the Neuropal transgene taken on the same animal to unequivocally identify the four posterior midbody neurons on the left side. Dashed lines in **b** indicate the walls of the trapping channel. Images in **a** are representative of *N* = 13, in **b** for *N* = 8 animals. **c** Average calcium activity of PDE in control (gray) and *mec-4* (cyan) mutant animals. Vertical lines indicate the onset and duration of the pressure pulse. Solid line indicates mean, shaded area indicates standard deviation. Stacked kymographs of the Ca^2+^ activity in **d** wild-type animals (*N* = 13), **e** *mec-4* mutant (*N* = 14) and **f** animals expressing tetanus toxin (TeTx) in touch receptor neurons (*mec-4*p:TeTx, *N* = 10). Red vertical lines indicate the onset and duration of the pressure pulse. **g** Representative recording of GCaMP6s signal in DVA after mechanical stimulation of the posterior body wall at times indicated by the red vertical bars. Stacked kymographs of the Ca^2+^ recording in DVA for **h** wild-type (*N* = 12, representative for 27 animals), **i** *mec-4* mutants (*N* = 9 animals) and **j** animals expressing TeTx in TRNs (*N* = 14 animals). Red vertical bars indicate time and duration of the mechanical stimulus.

We tested this prediction directly and investigated PDE and DVA activity after stimulating TRNs mechanically within a microfluidic pressure trap (Fig. 4a, b)^8^. To do so, we applied a high-frequency ‘buzz’ to the cuticle within the receptive field of PVM^36^ and recorded DVA and PDE activity using a GCaMP6s calcium reporter^35^ (Supplementary Movies 4 and 5). We used a panneuronal calcium reporter in conjunction with the Neuropal landmark transgenes^37^ to identify and visualize PDE activity (inset in Fig. 4a panel ii), together with PVM, PVD, and SDQ on the left side of the worm body (or just PVD and PDE on the right side). Intriguingly, we could also clearly distinguish mechanoreceptor activity in PVD and PVM, but not SDQ (Fig. 4a panel ii, Supplementary Movie 4), while PDE peak currents lagged behind that of other sensory neurons with a temporal delay of ≈200 ms. PDE responded consistently and strongly to mechanical stimuli (Fig. 4c, d) in a *mec-4* dependent manner (Fig. 4c, e). To understand if PDE receives postsynaptic input from posterior touch receptor neurons, we repeated these experiments in a transgenic animal expressing TeTx in TRNs. Consistent with the hypothesis that TRNs provide synaptic input to PDE, we observed a marked reduction in mechanically evoked PDE calcium responses upon TeTx-mediated silencing of synaptic transmission in TRNs (Fig. 4f, Supplementary Fig. 5a, b). In contrast, PVM remained mechanically responsive (Supplementary Fig. 5c), indicating that the defect is selective for downstream neurons rather than a general impairment of mechanotransduction. The residual PDE activity may therefore arise from intrinsic mechanosensitivity of PDE itself or from synaptic or extrasynaptic inputs originating from neurons other than TRNs. Together, our data show that PDE is postsynaptic and functionally connected to posterior TRNs. DVA also showed a multifaceted, context-dependent response to mechanical input, which required functional MEC-4 and vesicular release from TRNs (Fig. 4g–j). In many cases, the DVA neuron responded with a decrease in activity that recovered during the interstimulus interval (Fig. 4g, h and Supplementary Movie 5). Mutating *mec-4* and silencing TRN synaptic output with TeTx dampened DVA’s deactivation, yet paradoxically seemed to increase both the number and magnitude of secondary activation responses following mechanical stimulation (Fig. 4i, j). This effect of TeTx expression on PDE and DVA was different from the avoidance behavior to touch, as animals expressing TeTx in TRNs remained largely sensitive to mechanical stimuli (TRN:TeTx = 7.9 ± 1.5; ctrl = 8.8 ± 1.2; mean ± SD, *p* = 0.11; two-sided *t*-test). This indicates that increased TRN output causes a transient activation of PDE and a suppression of DVA activity. Because the loss of DVA activity is related to increases in body curvature^35^, our results suggest that body wall mechanosensation finetunes proprioceptive gain within the circuit. Together, these data show that TRNs are functionally connected to proprioceptive neurons, and therefore, a lack of input from TRNs to proprioceptors may lead to altered locomotion behavior.

### Touch receptor neurons sense substrate compliance

**Fig. 5:**
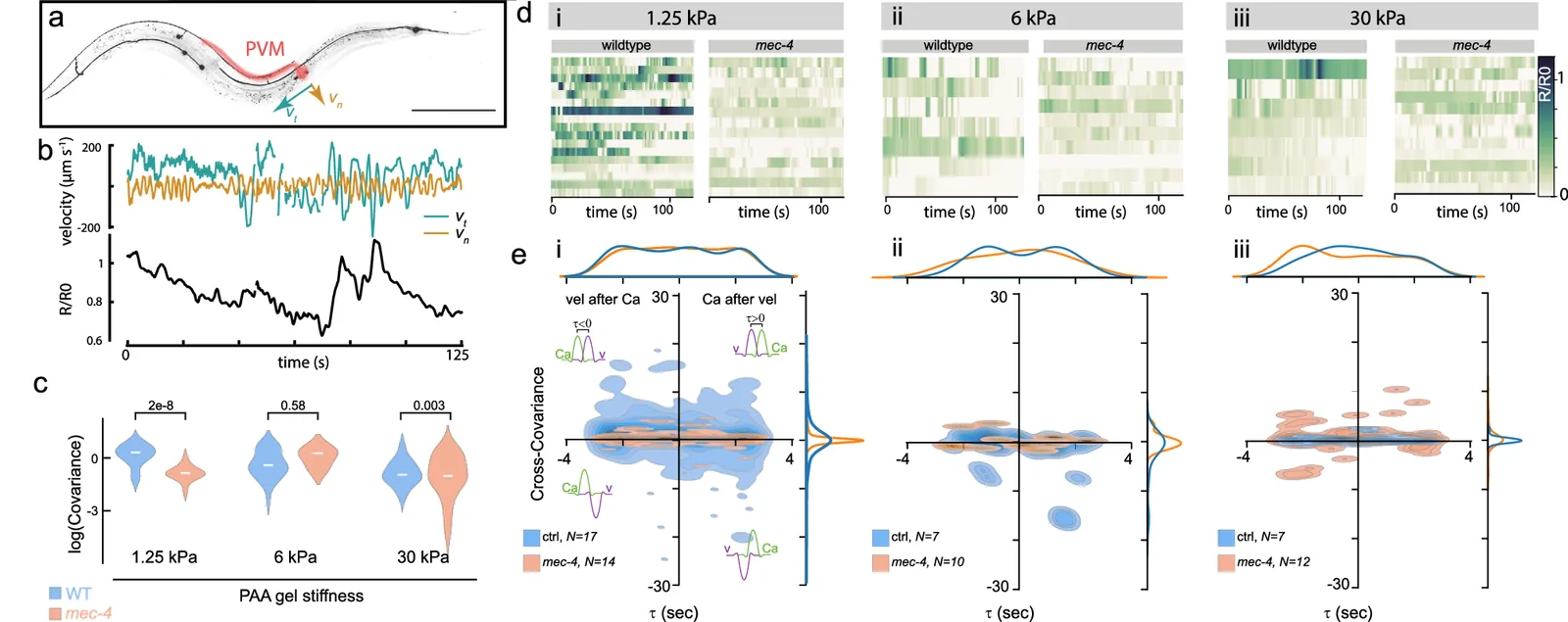
PVM senses substrate compliance. **a** Schematic of a worm and the location of the PVM neuron. Normal and tangential velocity vectors (*v*_n_ and *v*_t_, respectively) at the location of the PVM neuron are indicated as arrows. Scale bar = 100 μm. **b** Representative instantaneous tangential (*v*_t_) and normal (*v*_n_) components of the velocity on substrates of soft stiffness (upper) and recording of normalized, ratiometric calcium activity (*R*/*R*0, see the “Methods” section) in PVM (lower). **c** Covariance of PVM calcium activity and forward velocity on all substrates for control and *mec-4* mutant animals (see also Supplementary Fig. 6 for comparison with ALM and PLM). *p*-values derived from a two-sided Wilcoxon rank-sum test. **d** Kymograph of the ratiometric calcium activity extracted from PVM expressing GCaMP6s and tagRFP-T in freely moving animals (ctrl vs. *mec-4*) on different substrates. (i) 1.25 kPa; (ii) 6 kPa; and (iii) 30 kPa. **e** Cross-covariance between PVM calcium activity and the tangential component of the forward crawling velocity on (i) 1.25 kPa; (ii) 6 kPa; and (iii) 30 kPa substrates of ctrl and *mec-4* mutant animals, suggesting a plausible causal relation between neural activity and velocity. Negative timelags indicate PVM calcium transients that precede increases in velocity (e.g., due to self-inflicted body touch), whereas a positive timelag indicates that calcium transients follow bouts in velocity. Inset inside each quadrant indicates the temporal relation between the velocity and the calcium signals (see also Supplementary Fig. 8). *N* = number of animals analyzed. The top and right axes indicate the projected densities, to indicate the distribution of the measurements of each variable.

Our results from experiments (Figs. 1, 2 and 4) paired with connectome-level simulations (Fig. 3) suggest that the body wall mechanoreceptors directly sense the mechanical properties of the environment to influence proprioception during locomotion. To further test this idea, we next investigated whether TRN activity changes in animals crawling in different mechanical environments. To this end, we used animals expressing calcium-sensitive (GCaMP6s) and insensitive (tagRFP) fluorophores in TRNs of control and *mec-4* mutant animals, and recorded calcium fluctuations in PVM (Fig. 5a), PLM, and ALM (Supplementary Fig. 6) during free behavior on different PAA gels with an automated closed-loop image acquisition pipeline (see the “Methods” section). We occasionally observed increases in calcium activity that occurred secondary to self-inflicted contacts (e.g., nose-body contact), but also seemed uncorrelated with any behavioral syllable. As part of infrequent spontaneous calcium activity confined to the neurites of TRNs, we did not observe calcium transients in the cell body of completely immobilized animals (Supplementary Fig. 7). We hypothesized that activity increases in response to the velocity of the animals. We then quantified the independent velocity components, with the aim to relate the calcium dynamics to tangential and normal crawling velocities (Fig. 5b). Even though we observed larger and more frequent calcium transients that covaried with velocity in moving wild-type compared to *mec-4* animals in all body wall mechanoreceptor neurons (Fig. 5a, d and Supplementary Fig. 6, Supplementary Movies 6 and 7), the transients were larger on the softer gels (1.25 kPa), especially for the posterior neuron PVM (Fig. 5c). Indeed, time series of calcium activity and the instantaneous velocity co-varied (Fig. 5c), but only on softer substrates, and this difference between wild-type and *mec-4* mutant was largely dimished when animals crawled on 6 and 30 kPa gels (Fig. 5d). To determine the causal and temporal relationships between calcium activity in PVM, PLM and ALM, and the animal’s tangential velocity on different substrates, we computed the cross-covariance between these signals (see Methods) in both wild-type and *mec-4* mutant animals. We observed two dominant peaks in the cross-covariance between calcium signals and tangential velocity, suggesting the presence of both leading and lagging calcium dynamics relative to locomotion (Fig. 5e, see also Supplementary Fig. 8).

We first observed a pronounced neuronal activity pattern in PLM, but not in ALM, preceding bursts in locomotor velocity (negative lags in the covariance analysis), indicating that TRN activation is associated with subsequent increases in speed due to self-inflicted touches (Supplementary Fig. 6f). This is consistent with their established role in the gentle-touch escape response and, intriguingly, was also observed for PVM (Fig. 5e), despite ongoing debate regarding its contribution to forward escape behavior)^38,39^.

In addition, the cross-covariance analysis revealed a significant peak at positive lags, most prominently for PVM (Fig. 5e), but not for ALM or PLM (Supplementary Fig. 6f), indicating neuronal activity following increases in velocity (see the “Methods” section). To determine whether the observed temporal coupling between PVM calcium dynamics and forward velocity, rather than an effect of inner properties of individual time series of calcium and velocity, we performed surrogate testing by calculating the cross-covariance between calcium traces from one animal and the velocity of another (Supplementary Fig. 9, see the “Methods” section). We separately analyzed the cross-covariance between calcium activity and two orthogonal velocity components corresponding to the tangential (*v*_t_) and normal (*v*_n_) components of locomotion. While the experimentally measured data exhibited clear temporal organization in the tangential velocity component, surrogate datasets produced substantially weaker and less structured distributions (Supplementary Fig. 9). In contrast, the normal velocity component showed comparatively little organization and closely resembled the surrogate condition. These results suggest that TRN calcium activity is caused by tangential (forward/backward) locomotion, whereas the normal velocity component shows substantially weaker temporal organization.

Together, these findings suggest that PVM senses compliance of the physical environment and encodes the animal’s velocity relative to its substrate. These covariance signals were completely absent in *mec-4* mutant animals, suggesting that MEC-4 acts as a velocity-dependent sensor of substrate mechanics (Fig. 5e). Thus, our data show that body wall mechanoreceptors, especially PVM, exhibit calcium signals that co-vary with tangential velocity on soft substrates, consistent with velocity-modulated mechanosensitivity, leading to a nested, context-dependent neuronal activity.

**Fig. 6:**
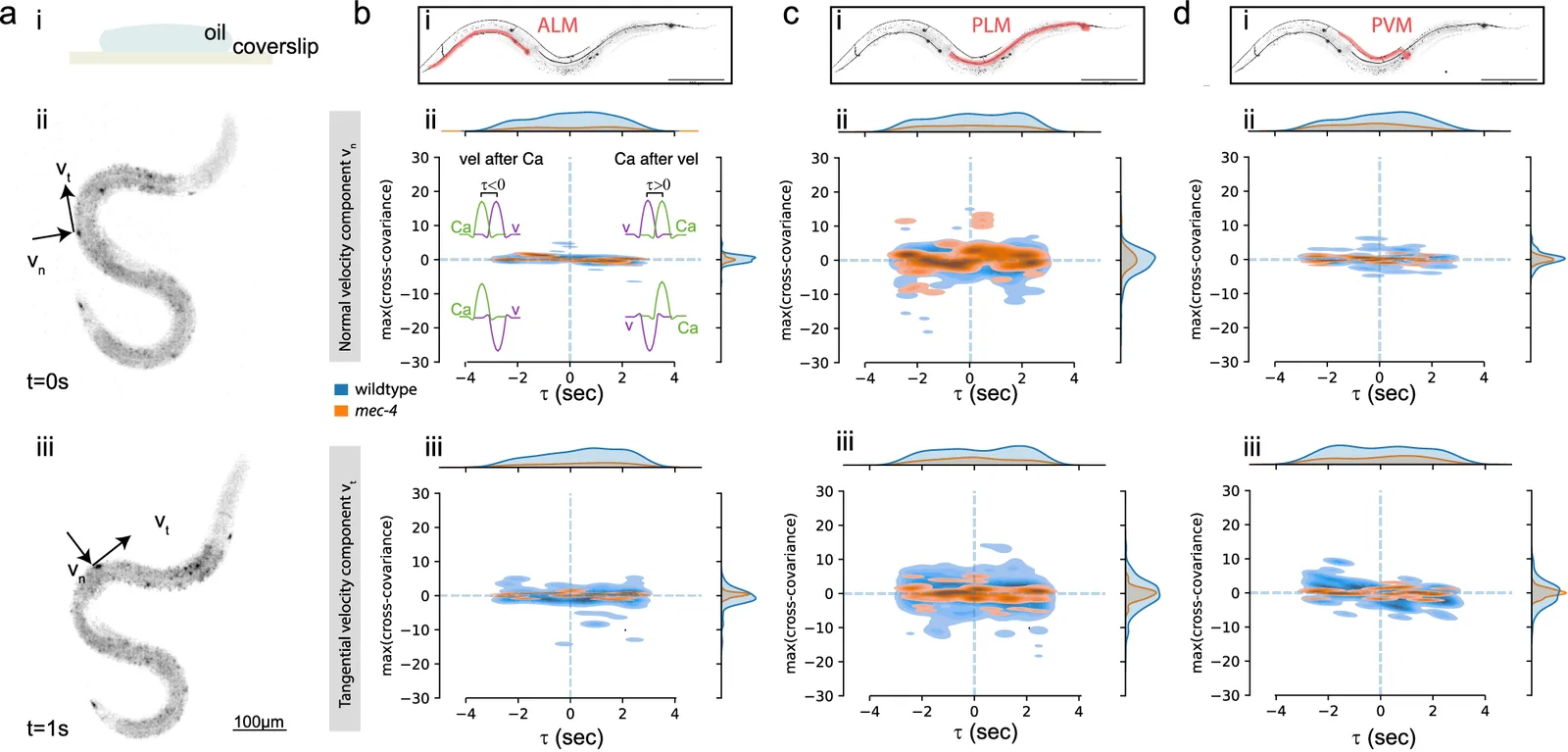
Viscous drag does not activate body wall mechanosensors. **a** (i) Schematic of the experiment of an animal immersed inside a flattened drop of Halocarbon oil. (ii) and (iii) Representative snapshots of an animal expressing GCaMP6s in TRNs crawling inside the oil droplet at two different time-points 1 s apart. The net velocity was decomposed into the normal and tangential components to visualize causal dependence between viscous drag and calcium activity. (i) Schematic representing the **b** ALM, **c** PLM, and **d** PVM neuron. (ii) and (iii) Plots showing the maximum cross-covariance of the calcium signal with the (ii) normal and the (iii) tangential velocity component. Blue, control; orange, *mec-4*. Negative timelags indicate calcium transients that precede bouts in velocity (e.g., due to self-inflicted body touch), whereas a positive timelag indicates that calcium transients follow bouts in velocity. Inset inside each quadrant of panel (**b** (ii)) indicates the temporal relation between the velocity and the calcium signals.

A key open question was whether the gentle body wall mechanoreceptors (TRNs) are activated primarily by shear stresses generated during crawling against a substrate, or whether viscous load on the body wall in a fluid is sufficient to elicit activation. To disentangle these possibilities, we immersed individual animals in biologically inert fluids of defined viscosity (Halocarbon oil, 190 mPa s). These animals maintained rhythmic crawling movements, albeit with increased body curvature (Fig. 6a), allowing us to compare neuronal responses in the presence of viscous forces but in the absence of substrate friction.

We imaged calcium activity in anterior and posterior TRNs during locomotion in oil and compared this to activity on soft hydrogels. While occasional transients were detected, especially in PLM, the overall TRN activity was strongly reduced and co-varied significantly less with locomotor kinematics (Fig. 6b–d) compared to crawling on soft PAA gels (Fig. 5).

But how much drag force does the animal experience moving through the viscous medium? Using resistive force theory for a slender cylinder of length *L* = 1mm and radius *a* ≈ 40 μm, moving laterally at velocity *v* = 50 μm/s in a fluid of viscosity *η* = 0.19 Pa s, we obtain a transverse drag per unit length of ${f}_{\perp }\approx \frac{4\pi \eta v}{\ln (L/a)+1/2}\approx 1.2\times 1{0}^{-4}\,{{{\rm{N}}}}/{{{\rm{m}}}}$.

Over a TRN-relevant length scale of Δ*x* = 50 μm this corresponds to a local load of only *F*_viscous_ = *f*_⊥_Δ*x* ≈ 6 nN. This value is at least an order of magnitude below the ≈100–200 nN indentation forces reported to reliably activate TRNs with a glass probe^14^, supporting the conclusion that viscous load is insufficient to robustly engage TRNs.

However, during crawling on agar, frictional forces can be estimated as *F*_friction_ = *μN*, where *μ* is the effective coefficient of friction and *N* is the normal load. Traction force studies report effective frictional forces in the range of 10−100 μN per animal^40^. On the scale of a 50 μm TRN segment, this corresponds to hundreds of nN of local load - well above the activation threshold for MEC-4-dependent mechanotransduction.

Together, both the experimental recordings and the force estimates indicate that viscous drag alone is not sufficient to strongly activate TRNs. Instead, TRNs are robustly engaged by shear stresses that arise from frictional coupling to a soft, viscoelastic substrate during crawling.

### Disruption of MEC-4 in PVM elicits a lethargic locomotion phenotype

**Fig. 7:**
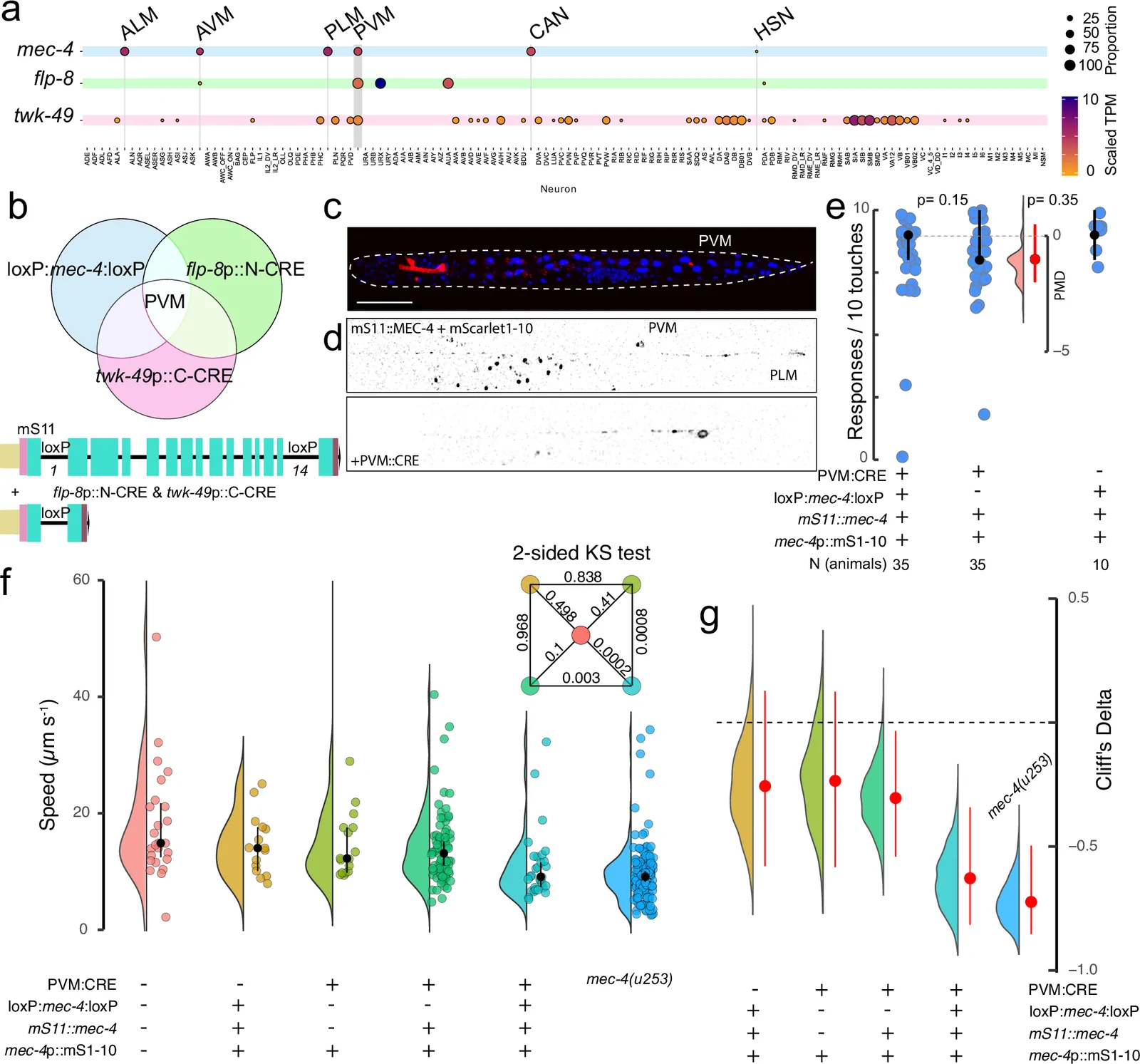
MEC-4 disruption in PVM causes a lethargic locomotion phenotype. **a** Single-cell RNA expression profile of *mec-4*, *flp-8*, and *twk-49* in all neurons of adult hermaphrodites, indicating their overlap exclusively in PVM. TPM transcripts per million. Data taken from ref. ^20^. **b** Design of the strategy. A split CRE recombinase expressed under the control of *flp-8p* (N-terminal domain) and *twk-49* (C-terminal domain) promoters, together with the loxP-flanked *mec-4* locus. LoxP sites were introduced into introns 1 and 14. The 5' end was fused in-frame to mScarlet barrel 11 (mS11) for visualization upon complementation with mScarlet1-10. **c** Representative image for *N* = 21 CRE reporter animals, indicating CRE activity through a blue-to-red color switch. Scale bar = 100 μm. **d** Representative images of control and PVM::CRE animal with intact and disrupted MEC-4 expression in PVM visualized by the presence or absence of mScarlet (*N* = 14). **e** Average touch-evoked escape responses to a sequence of 10 consecutive touches in control animals versus animals in which *mec-4* is disrupted specifically in PVM. Black circle indicates the median ± 95% confidence interval of the median. *p*-value indicated derived from a two-sided Mann–Whitney *U*-test. Number of tested animals is indicated above each category. Floating axis shows the bootstrapped effect size as paired mean difference (PMD). **f** Violin plot of the distributions of worm locomotion speeds with the conditional *mec-4* ablation exclusively restricted to the PVM neuron across different genotypes. Individual data points represent the average speed of each animal, and black dots indicating medians ± 95% confidence interval. PVM::CRE = split CRE; mS11::*mec-4 *= barrel 11 of mScarlet fused to the N-terminus of MEC-4; *mec-4*p:mS1-10 = barrels 1–10 of mScarlet expressed under the *mec-4*. Inset: Two-sided Kolmogorov–Smirnov test *p*-values comparing each pair of distributions (numbers inside the matrix), highlighting the degree of distributional overlap between genotypes. Colors correspond to genotype groups consistently across violins, estimation plots, and the KS matrix. **g** Estimation plots of bootstrapped Cliff’s Delta (red points indicate median, red bars show 95% bias-corrected confidence intervals) for each comparison relative to the reference genotype. A dashed horizontal line at zero indicates no effect.

Up to here, our results support a model in which PVM detects substrate-dependent mechanical cues during crawling. We next asked if the PVM neuron has a specific role and modulates locomotion through the expression of MEC-4 ion channels. To address this question, we generated a transgenic strain in which a split CRE recombinase^41^ was co-expressed under the control of two promoters that intersect uniquely with *mec-4* expression in PVM neurons (Fig. 7a, b). This construct was introduced into a genetic background in which the endogenous *mec-4* locus was flanked by loxP sites (Fig. 7b). After assessing the specific recombination of the two loxP sites specifically in PVM^35,42^ (Fig. 7c) we confirmed the loss of MEC-4 expression in PVM using a functional split-mScarlet-tagged MEC-4 allele^43^, which exhibits wild-type punctate localization (Fig. 7d) and touch sensitivity (responding to 8.85 ± 1.23 out of 10 touches, mean ± SEM, *N* = 20 animals), comparable to control animals with either split-CRE or floxed *mec-4* locus alone (Fig. 7e). Finally, we asked whether selective disruption of *mec-4* in PVM induces a lethargic locomotion phenotype. Compared to animals that expressed the split CRE alone or the loxP-flanked *mec-4* allele alone, double-transgenic animals lacking *mec-4* specifically in PVM exhibited significantly reduced locomotor speed and increased lethargy (Fig. 7f, g). Together with our calcium-imaging data, these results indicate that MEC-4 expression in PVM has a role in substrate mechanosensation important for animal locomotion.

### MEC-4-dependent mechanosensation of traction forces shapes locomotion

**Fig. 8:**
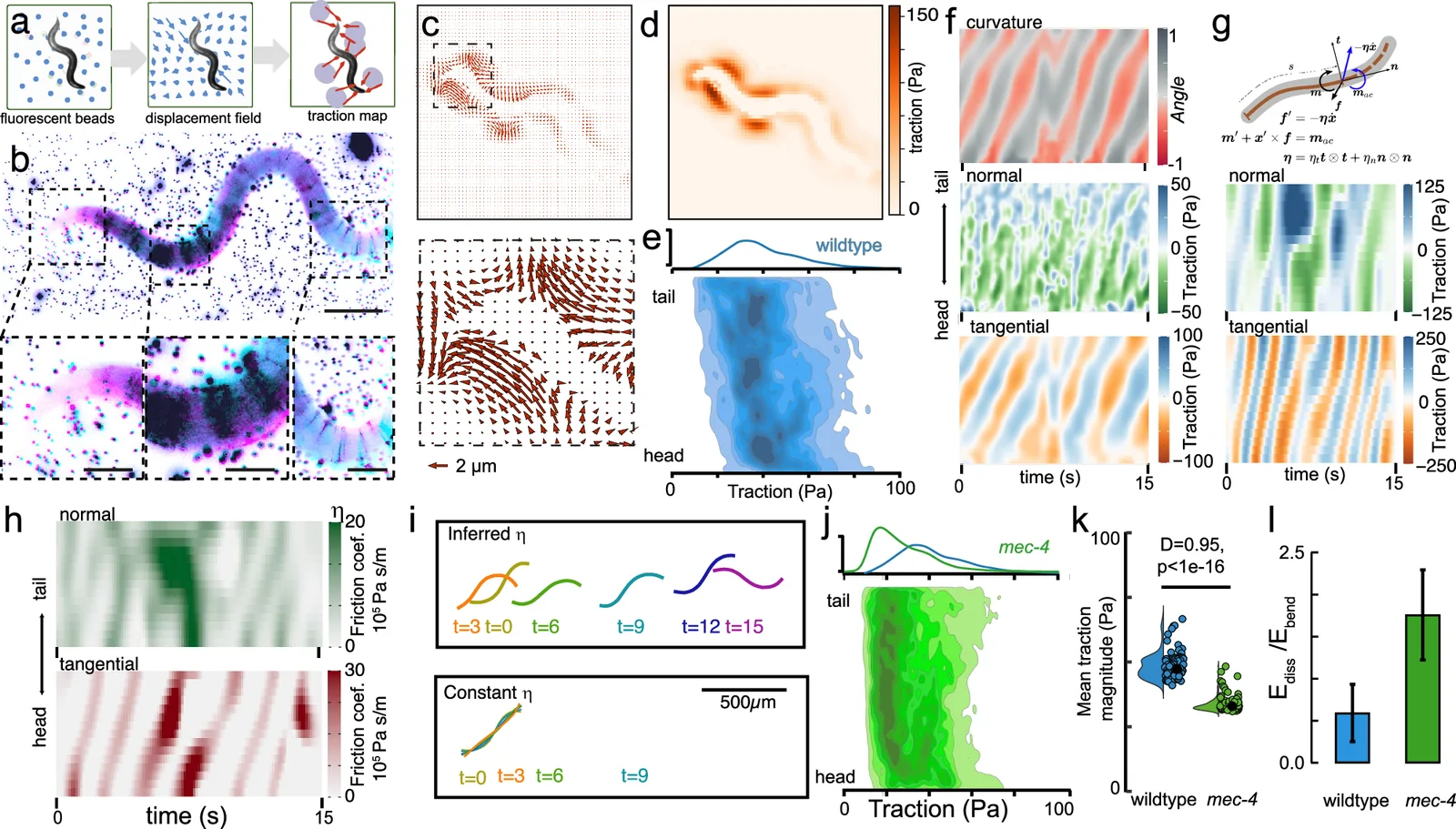
MEC-4 is required for optimal substrate traction forces. **a** Schematic of the traction force experiment, the local displacements of the PAA gel-embedded microspheres in the vicinity of the worm body are transformed into a traction field. **b** Representative image sequence for two successive frames, false-colored from a video of a worm crawling on an elastic substrate. Scale bar = 100 μm. Insets show the displacement of the gel-embedded microspheres as the animal is crawling on the substrate. Pink, frame *#n*; Cyan, frame *#n* + 1 **c**, Representative displacement field of a worm crawling on the gel for *N* = 8 experiments. Length of the arrows corresponds to bead displacements. Inset shows magnified regions in the upper map. Scale bar = 40 μm. **d** Color map showing the uneven distribution of tractions exerted on the substrate. **e** Quantification of the mean traction force vs body coordinate for wild-type animals (*N* = 8) crawling on 1.25 kPa PAA hydrogels. For 6 and 30 kPa, see Supplementary Fig. 10. **f** Curvature kymograph along the body coordinate and the corresponding normal and tangential traction map as a function of time. **g** Explanation and formulation of the mechanical model and optimal control problem. Kymographs represent the time course of the normal and tangential tractions inferred from the worm bending/locomotion data. **h** Representative kymographs of the distributed normal and tangential friction coefficient between the animal body and the substrate, inferred from the worm bending/locomotion data using the model shown in **g**. It converts the sliding velocity of the body relative to the substrate into a shear stress acting on the body. **i** Simulations of an animal with the inferred inhomogeneous friction coefficient and an animal with a constant friction coefficient, using the same inferred muscle torques. See also Supplementary Movie 9. **j** Quantification of the mean traction force vs body coordinate for *mec-4* mutant animals (*N* = 9). **k** Comparison of the average spatial traction magnitude for ctrl and *mec-4*. Each point indicates the average of each body coordinate for wild-type (*N* = 8 animals) and *mec-4* (*N* = 9 animals). Black dot indicates the median ± 95% confidence interval of the median. *p*-value above the plot derived from a 2-sided KS test with the test statistics D indicated. **l** Ratio of dissipated and bending energy for wild-type and *mec-4* mutant animals (*N* = 360 points from three independent biological replicates). Mean  ± standard deviation.

Effective crawling locomotion on a substrate requires sufficient friction or traction to generate forward movement. Traction in a normal direction in one part of the body is needed to generate the propulsive force required to overcome the resistive friction force acting in other regions of the body^44^. Thus, optimization becomes a paradoxical process—minimizing traction at some parts and directions of the system while maximizing it in other regions and orientations. How is this achieved in moving animals? Our data up to here suggest that TRNs serve as the neuronal substrate to sense the friction force in a velocity-dependent manner. To visualize and quantify the worm’s interaction with its substrate while crawling, we performed a traction force microscopy experiment and imaged the displacement of fluorescent microspheres on the surface of PAA hydrogels (Fig. 8a, b; Supplementary Movie 8). Here, bead displacements are proportional to the interaction forces between the animal and the surface it crawls on. Assuming a linear resistive force model, these forces are proportional to the friction coefficients and the velocity of the body part in contact. Intriguingly, we observed that the tractions produced by the crawling animals are not homogeneous, but display greater amplitudes near the head (Fig. 8c) and higher bead displacement; thus, different tractions are associated with different parts of the body (Fig. 8d, e). To interpret this spatial anisotropy in traction forces, we next turned to resistive force theory^45^, which provides a mechanical framework to relate body motion with substrate interactions. According to resistive force theory, the normal (perpendicular) drag force experienced by *C. elegans* during movement in a viscous environment is greater than the parallel (tangential) drag force. To check the validity of this assumption, we decomposed the traction field into its components normal and tangential to the worm’s centerline. In fact, we found that the force components are well correlated with the movement of the worm (Fig. 8f) and also that the vectors associated with the normal motion are twice as large as those with tangential motion. This suggests that worm locomotion can be described by applying linear resistive force theory^45^. To find the profile of the friction forces and coefficients along the worm’s body, we set up a mechanical model that includes different friction coefficients along tangential and normal directions of the worm midline (Fig. 8g, see Supplementary Discussion). We used this model and an optimal control formulation^46^ for solving the following inverse problem: infer the friction coefficients and muscle torques at each time and location from the experimental measures of worm positions. Consistent with the experimentally derived tractions (Fig. 8f) and from our model (Fig. 8g), the solution of the inverse problem reveals that the friction coefficients are spatially heterogeneous along the body of the worm (Fig. 8h), suggesting that this inhomogeneity contributes to optimized locomotion. This distributed friction coefficient suggests that different regions of the worm body mechanically couple to the substrate during locomotion. The relatively high magnitude means that even small sliding velocities generate substantial traction forces, and the worm does not “slip” easily. Indeed, when the same inferred internal torques were applied to a mechanical model with homogeneous and isotropic friction coefficients, the simulated animals were unable to generate substantial forward propulsion (Fig. 8i; Supplementary Movie 9). This raises the question: do TRNs encode information about local substrate coupling, allowing the worm to regulate its effective traction through changes in posture and velocity?

We thus repeated the traction force experiment in the *mec-4* mutant. Interestingly, we found that the traction forces of the *mec-4* mutants were generally lower and more homogeneous than those of wild-type animals, especially on soft substrates (Fig. 8j and k; Supplementary Fig. 10). Similar to wild-type animals, *mec-4* produced higher tractions on stiffer substrates (Supplementary Fig. 10b, c). However, the magnitude and distribution of traction forces exhibited substantial overlap with those measured in wild-type animals on stiffer substrates (Supplementary Fig. 10). Because *mec-4* mutants exhibit defects in posture and traction force generation, these findings suggest that MEC-4 is required to optimize locomotion, particularly on soft substrates. To further support this interpretation, we calculated both the bending energy and the energy dissipated during locomotion. We found that energy dissipation is substantially increased in *mec-4* animals (Fig. 8l and see the “Methods” section). The elevated energy dissipation in *mec-4* mutants, combined with the higher and more spatially organized tractions in wild-type animals, suggests that mechanosensory feedback through MEC-4 improves the mechanical efficiency of locomotion.

**Fig. 9:**
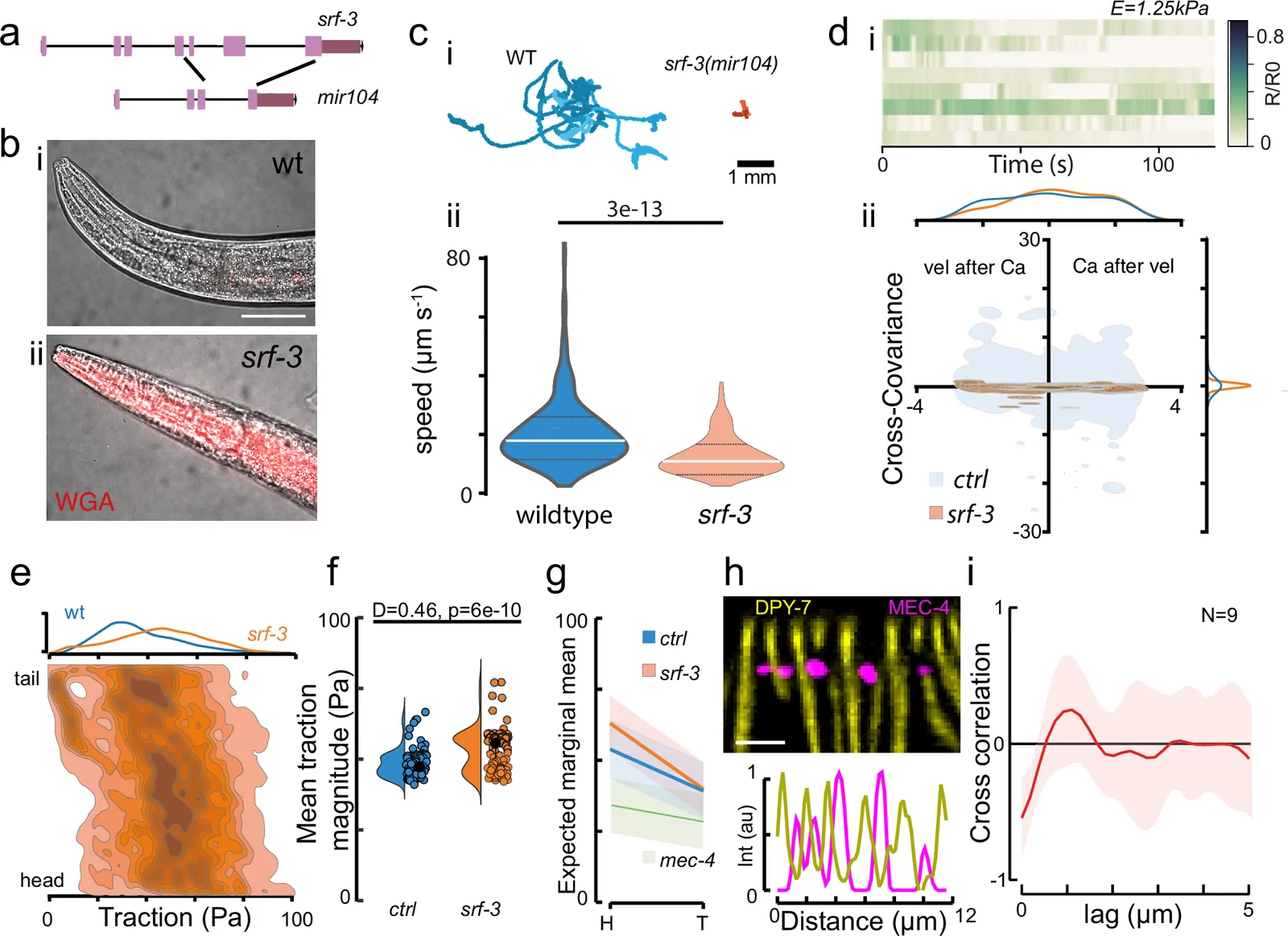
The cuticle surface influences traction forces and locomotion. **a** Genomic organization of the *srf-3* locus and the schematics of the *mir104* allele. **b** Staining of (i) wild-type and (ii) *srf-3(mir104)* animals with wheat germ agglutinin (WGA). Note, the absence of the *srf-3* - dependent glycocalyx leads to strong WGA staining of the collagen cuticle. Scale bar = 40 μm. Representative results for *N* = 16 imaged animals per condition. **c** (i) Displacement of the *srf-3* and wild-type animals on *E* = 1.25 kPa elastic substrates in the 5 min experimental window. (ii) Comparison of the crawling speed between wild-type (*N* = 266) and *srf-3* mutant (*N* = 112) animals on normal NGM agar containing OP50 bacteria. White lines show the median; dotted lines represent the 25th and 75th percentiles. p-value derived from a two-sided KS test. **d** Calcium dynamics in PVM of *srf-3* mutant are absent in moving worms. (i) Kymographs of PVM activity during free locomotion on 1.25kPa PAA gels. (ii) Cross-covariance plot between calcium signals and velocity. **e** Quantification of the mean traction force vs. body coordinate for *srf-3* mutant animals (*N* = 9) on 1.25 kPa PAA. **f** Comparison of the average spatial traction magnitude for control and *srf-3* mutant animals on 1.25 kPa PAA gels. Each point indicates the average of each body coordinate for each strain. Black dot indicates median ± 95% confidence interval of the median. *p*-value above the plot derived from a 2-sided KS test with the test statistics *D* indicated. **g** Model-derived traction force profiles along the body axis (Head–Tail) for each strain obtained from a linear mixed-effects model. Solid lines represent expected marginal means (EMMs) of traction force as a function of normalized body position, while shaded regions indicate 95% confidence intervals. Estimates were derived from a mixed-effects model including strain, body position, and their interaction as fixed effects, with worm identity included as a random intercept. The model revealed significant effects of strain (*F*_2,⋅_ = 5.21), body position along the worm (*F*_1,⋅_ = 1419.26), and their interaction (*F*_2,⋅_ = 179.94) on measured traction forces. **h** Representative confocal image and intensity distribution of a worm labeled with DPY-7::GFP and mScarlet::MEC-4 to visualize colocalization of the cuticle furrows and mechanoreceptor channels, respectively. Scale bar = 5 μm. Images representative of *N* = 16 animals per condition. **i** Average cross-correlation between the two fluorescent markers obtained from *N* = 9 animals. Mean ± standard deviation.

Lastly, we sought to identify the molecular components that mediate frictional coupling between the worm and its substrate, providing a physical basis for the generation of traction forces. The cuticle of *C. elegans* is coated with an elaborate glycocalyx^47^. SRF-3, a nucleotide sugar transporter in the Golgi apparatus, is expressed in hypodermal seam cells, leading to altered cuticle glycoconjugates on the animal’s surface. While some *srf* mutants show severe locomotion defects and fragile cuticles, *srf-3* mutants appear wild-type despite altered surface antigenicity^48^. Using CRISPR/Cas9, we created a *srf-3* loss-of-function allele (Fig. 9a), which led to strong cuticle staining with wheat germ agglutinin (Fig. 9b)^48^, but retained wild-type touch sensitivity (8.4 ± 1, mean ± SD, *N* = 30 animals). Although the crawl speed on agar was slightly lower (Fig. 9c), neither body shape nor cuticle structure was affected by *srf-3* loss of function, as determined by quantifying the distribution of DPY-7, a label for cuticle furrows (Supplementary Fig. 11). We first performed our calcium imaging in PVM in unconstrained animals on soft PAA gels. In particular, *srf-3* mutants struggled to move forward on soft PAA gels, and, unlike wild-type animals, we did not observe substantial calcium fluctuation that was correlated with their forward velocity component (Fig. 9d). We then tested how the altered glycocalyx, due to *srf-3* loss-of-function, changed surface interaction of the worm using traction force microscopy. To our surprise, *srf-3* mutants generated higher traction across all substrates independent of the gel’s elastic modulus, suggesting that wild-type worms are better lubricated and a lack of the correct glycocalyx increased friction forces with the substrate (Fig. 9e, Supplementary Fig. 10).

Importantly, the traction between the different strains varied significantly along the body of individual animals (linear mixed effects model, see the “Methods” section; body position: *F* = 1419.26, *p* < 0.001) and differed between strains (strain: *F* = 5.21, *p* < 0.01). Here, the interaction between strain and body position was statistically significant (strain × body position, *F* = 179.94, *p* < 0.001), indicating that the strains differed significantly in spatial traction distribution along the body, not merely a global shift in traction magnitude (Fig. 9f). Visual inspection of the estimated marginal means showed that the strains diverged mainly in the head and midbody regions, while converging near the tail (Fig. 9g). These results suggest that the mechanical interaction of the body wall is modulated by *srf-3* and *mec-4* sensory dependent, but differences are localized rather than uniform across the body for all gels tested.

Lastly, in an effort to understand how animals use their TRNs to sense surface mechanics, using fluorescent marker strains, we quantified the relative distribution of MEC-4 ion channels and DPY-7 cuticular collagens, a marker for circumferential furrows^49^. We found MEC-4 localized in discrete puncta that, on average, did not colocalize with the furrow marker DPY-7 (Fig. 9h, top image), suggesting that MEC-4 colocalizes with the cuticle ridges. This observation evokes an intriguing idea: given that MEC-4 ion channel complexes are aligned beneath circumferential ridges, it is plausible that cuticle ridges act as sites of frictional force transfer from the substrate, thereby channeling mechanical stresses directly to MEC-4.

## Discussion

Our findings reveal that *C. elegans* use their gentle mechanoreceptor neurons to sense mechanical stress rates during crawling locomotion. We demonstrated that these neurons actively detect forces between the body and the substrate, fine-tuning postural gait through proprioceptive feedback. Active sensing involves organisms or systems that generate movements to obtain sensory information about their environment, rather than passively receiving input^50^. In active sensing, the organism or system takes control of how it explores and interacts with its surroundings to optimize information gathering. Animals such as rodents move their whiskers to sense textures^51^, while human fingertip ridges enhance high-frequency vibrations during contact, improving tactile perception^52^. Similarly, we here propose that *C. elegans* touch receptor neurons are active sensors to facilitate substrate sensing, during which forward motion helps to gather detailed information about the mechanics of the environment. Friction forces are complex and depend on the contact area (thus load), asperities of the interface, and surface chemistry^53^. The structured cuticle of *C. elegans* may therefore act similarly to the ridges on human fingertips to increase friction forces between the skin and the surfaces, providing dynamic frequency-dependent sensory input. In ventral mechanosensors of wild-type animals such as PVM and AVM, MEC-4 aligns with the cuticle annuli (ridges between furrows, Fig. 9h (i)). Because these cuticle structures are absent in the lateral neurons (which we found to be less sensitive to substrate mechanics), we propose that mechanical forces are conveyed through the cortical fibrous organelles, cable-like structures of the epidermis that are aligned with the cuticular annuli, and thereby coupled to mechanosensitive ion channels in the touch receptor neurons^54^.

TRNs respond rate-dependently, making them largely unresponsive to slow or stationary stimuli^8,18^. They only activate during a change in stimulus or movement. When an organism moves across a surface, it generates traction forces that vary with velocity. This traction force is due to the relative velocity of the substrate and changes in space and time. We propose that it is this change in traction, caused by movement, that generates a dynamic force on neurons. Mechanically, our simulations demonstrate two necessary conditions for effective crawling: the tangential friction coefficient (and tractions) must be low enough to overcome drag, and normal friction must be high enough to provide propulsion. Our calculated tangential friction coefficient of ≈3 × 10^6^ Pa s/m (Fig. 8h) indicates strong mechanical coupling between the worm and the substrate, suggesting that small body movements generate substantial mechanical stresses on the cuticle that could be sensed by the underlying MEC-4 ion channels. For local slip velocities of 10–100 µm s⁻¹, this coefficient corresponds to tangential stresses of approximately 30–300 Pa and, assuming an effective local contact area of 10⁻⁹ m², interaction forces of roughly 30–300 nN, the same order of magnitude of contact forces known to activate TRNs^14^ (see Supplementary Discussion). Wild-type worms seem to modulate these two conditions efficiently by sensing and adapting their traction force. Some partial explanation for this modulation may be found by resorting to more sophisticated friction laws, such as lubrication theory^55^, where frictional coefficients depend on the curvature of the groove and the worm cross-section. Consequently, by changing the curvature of the cross-section while bending, the worm may be able to modify the contact area and the drag coefficient ratio between normal and tangential friction coefficients. Indeed, *mec-4* animals show proprioceptive defects and thus are not able to properly synchronize their muscle activity with their curvature profile.

While our experiments were conducted on uniform agar substrates, *C. elegans* naturally inhabits highly heterogeneous environments such as decomposing plant matter, biofilms, and soil micropores^56^. These settings present spatially variable friction, compliance, and adhesion. Friction sensing may therefore not only modulate gait on smooth surfaces but also enable the worm to detect and adapt to local terrain properties in three-dimensional microenvironments. For example, increased friction could facilitate anchoring during high-force turns or burrowing, whereas low-friction regions might trigger adjustments in posture or direction to maintain propulsion. Although agar assays do not capture the full complexity of these environments, our findings provide a mechanistic basis for how frictional feedback could shape locomotion in natural substrates^57^.

We have shown that *C. elegans* actively produces inhomogeneous traction on elastic substrates and thus shares the characteristic with other limbless animals, such as snakes^58^. Because the highest traction is exerted in the anterior part, animals can use the muscles in the head to actively steer toward the substrate to maximize sensory information. In the absence of this information the *mec* mutants ‘lose orientation’, do not actively adjust friction forces and cease crawling, producing the lethargic phenotype. Intriguingly, lab-cultivated strains seem to have lost the higher effective drag anisotropy, which means the wild isolates crawl with less surface slip^45^.

In the absence of food on soft viscoelastic substrates, body wall mechanoreceptors, especially PVMs, exhibit velocity-dependent activity consistent with sensing substrate mechanics; the magnitude and expression of these phenotypes are modulated by feeding state, sex, and substrate. Why are TRNs, and PVM in particular, exquisitely sensitive to soft substrates but not to stiffer substrates? TRNs are not the only body-wall mechanosensors, and PVD neurons are known as high-threshold mechanosensors. This lower sensitivity may, therefore, enable mechanosensation on stiffer substrates. Together, a degenerate circuit system^59^ and molecules with increasing sensitivity may therefore ensure optimal locomotion in varying and dynamic mechanical environments.

## Methods

### Animal maintenance

Animals were grown and maintained according to standard protocols^60^. Synchronization was performed as stated previously^61^, and animals were left in the 20^∘^C incubator until they reached the young adult stage (approximately 52 hours, subjected to strain variability).

### Molecular biology, transgenesis and genome editing

All plasmids listed in Supplementary Table 1 were generated using the Gibson assembly method. All coding sequences were verified by sequencing. Transgenic strains were generated by microinjection into the worm gonad^62^. When multicopy extrachromosomal arrays were integrated, the UV/TMP strategy was used^26^. CRISPR edits were performed according to standard protocols^63^. MSB952 was generated using FLint as described^64^. Unless otherwise specified, crRNAs, tracrRNAs, ssODN, gBlocks, and Cas9 were purchased from IDT Technologies. All strains were outcrossed and on-target edits were verified by sequencing.

#### Conditional knockout of *mec-4* in PVM

50 ng/μL of each pNMSB184 and pNMSB185 (see Supplementary Table 1) were coinjected with 25 ng/μL of pCFJ68 (*unc-122*p::GFP) into SV2049 worms giving rise to MSB1492. Integration of this array produced strain MSB1501.

#### Expression of tetanus toxin in TRNs

Background strains MSB273 (for DVA, ref. ^35^) or MSB661 (for PDE) were injected with 20 ng/μL pNMSB186 (*mec-4*p::mScarlet::SL2::TeTx), 1 ng/μL pNM50 (*myo-2*p::GFP) and 80 ng/μL of 1 kb Plus DNA ladder (Invitrogen) to generate MSB1505 and MSB1542, respectively. MSB1524 was the result of integrating the array in MSB1505.

#### Split mScarlet::MEC-4

To visualize endogenous MEC-4 expression, we used a split fluorescent protein approach^43^ in which we introduced the 11th *β*-barrel of mScarlet before the first exon of the gene, with a GGSGG flexible linker between them. We used two crRNAs (#51: 5’ CGGATGGGTCCCGAAGGTGT 3’ and #52: 5’ TGTACTCGGATGGGTCCCGA 3’) targeting a few bases upstream of the first exon of *mec-4* and a ssODN (tgctattttttttatcgctatcaagttatagaatgTACACCGTCGTCGAGCAATACGAGAAGTCCGTCGCCCGTCACTGCACCGGAGGAggtggatctggaggtatgtcatggatgcaaaacctgaaaaactaccaGcaTctCcgAgaTccatccgagtacatgtcccaggtttatggagaccc) (barrel 11 UPPERCASE) with 35 bp homology region upstream and downstream of the insertion and 5 silent mutations (indicated in UPPERCASE) to prevent targeting of the crRNAs to the ssODN. The resulting strain (MSB797) was outcrossed once to the parental KG1180 (*lite-1*) to remove the *dpy* phenotype, leading to MSB931. It was then injected with *mec-4*p::mScarlet 1–10 (pNS50, 10 ng/μL) as an extrachromosomal array to restrict expression selectively to TRNs, together with a co-injection marker for green coelomocytes (pCFJ68, *unc-122*p::GFP, 30 ng/μL), leading to MSB932 *mir50*(mScarlet(11)::*mec-4*); *mirEx384*(*mec-4*p::mScarlet1-10; *unc-122*p::GFP). The extrachromosomal array was UV integrated leading to MSB943 *(mir50*(mScarlet(11)::*mec-4*); mirIs93(*mec-4*p::mScarlet1-10; *unc-122*p::GFP). Both strains were sensitive to gentle touch to the body wall: MSB932 9.35 ± 1.42; MSB943: 8.85 ± 1.23 (mean ± SEM, *N* = 20 number of individual animals).

#### *mec-4*::active aptazyme (AZ)

We introduced an active aptazyme^29^ 16 bp downstream of the *mec-4* stop codon by using a crRNA (#78: 5’ tttaagacacaacattgcaa 3’) annealing at the 3’ UTR of the gene to generate *mec-4(mir85)*. A 35 bp homology arm, double-stranded DNA amplified from a gBlock with KOD Hot Start DNA Polymerase (Novagen, ref. 71086) was used as repair template (5’ tgcgtctggatctttctgaatttgttttttcttgtCAAACAAACAAAGGCGCGTCCTGGATTCGTACAAAACATACCAGATTTCGATCTGGAGAGGTGAAGAATACGACCACCTGTACATCCAGCTGATGAGTCCCAAATAGGACGAAACGCGCTCAAACAAACAAAtttaaagttaccattgcaatgttgtgtcttaaaataaaaatttacatgagaataatt 3’, aptazyme in UPPERCASE). With this approach, we generated two strains, MSB1137 on a wild-type background, and MSB1549, with the mScarlet::*mec-4* background (MSB943). Functionality of the aptazyme to reduce RNA levels was tested in RT-PCR on MSB1137 (Supplementary Fig. 3b, c). Briefly, worms incubated for 5 hours with the stated Tetracycline concentration were pelleted and washed twice with M9. Seven volumes of TRIReagent (TR-118 MRC) were added to the packed pellets and stored at −80 °C. Next, 5 cycles of freeze/thawing/vortexing were performed, and samples were allowed to stand at room temperature (RT) for 5 min. Addition of 0.2 ml of chloroform per ml of Trizol used was carried out before a 2–3 min RT incubation followed by a 15 min centrifugation at 4 °C and 12,000x*g*. The aqueous phase was transferred to a new tube, and 0.5 ml of isopropanol per 1 ml of Trizol was added. After a 10 min RT incubation, samples were centrifuged for 15 min at 12,000×*g* and 4 °C. Supernatant was then removed and pellet washed with 1 ml 75% ethanol, centrifuged for 5 min, 4 °C, and 7500×*g*. Supernatant was removed, and the pellet was allowed to air-dry before using RNAse-free water to resuspend. Quality and quantity were assessed on a NanoDrop with serial dilutions. RT-PCR was performed using Superscript IV One-step RT-PCR System (Invitrogen, ref. 12595100). Primers for *mec-4* amplification were FW 5’-TCTGACTCATATTGACCCTGCGTTTGG-3’ and RV 5’-TGGTTGTTGGCATCTGCAACGG-3’, giving rise to a  ~ 540 bp fragment. For *dat-1* amplification, primers were FW 5’-AGAACAGTGGTCTGGAAAGCTGGAC-3’ and RV 5’-GCCCATGGCAGGTTAAAACTGAATGAAG-3’ and the resulting amplicon was  ~ 350 bp long. The recovery of the mScarlet fluorescence signal (MSB1549, Supplementary Fig. 3d) was performed as previously reported^43^. Briefly, adult worms were allowed to lay eggs on 10 μM tetracycline plates, and the F1 mounted, once young adults (YA, 20 °C), on 2% agarose pads in 3 mM levamisole^61^. Fluorescence images were taken using an inverted confocal microscope (Nikon Ti2 Eclipse) with a 60X 1.4 NA oil immersion lens. mScarlet was excited using the 561 nm laser at maximum laser power and transmitted through a 594 nm emission filter. Exposure time was 100–200 ms, depending on the strain to image.

#### Deletion allele *srf-3(mir104)*

We generated a deletion mutant for *srf-3* in the wild-type background, covering from exon 5 until the end of the gene. We used two crRNAs, one targeting intron four (#87:5’ gtttgtgtgtaatgatgacg 3’) and the other at the end of the last exon (7, #88: 5’ CGAGATTCCGTCTACAAAAG 3’). We also used a repair template ssODN (5’ taatatacagacgagaatttacagacaaaacgcctTtAtCacacTcaaaccatgaaagttgcggcgtctaaatgtgaagctg 3’) with 35 bp homology regions and four mutations (UPPERCASE) to generate a premature stop codon. However, the sequencing results reported a slightly different repair, still generating a premature stop codon: 5’ taatatacagacgagaatTTACAGACAAAACGCCTcTTAGACGTATCTCATTACGATCATCACGATCATCGATCATTACGATCATCATTACACACaaacCATGAAAGTTGCGGCGTCTAAATGTGAAGCTG 3’

#### Deletion allele *mec-10(mir110)*

We proceeded to generate a deletion mutant for *mec-10* in the *mec-4(u253)* background (TU253). We used two crRNAs, one targeting the first exon (#81:5’ GGTTGAAATTTTGACATTCG 3’) and another overlapping the last exon and the 3’ UTR of the gene (#82: 5’ GAgagccatgagaaaaaagg 3’). We also used a repair template ssODN (5’-tccgttgattctgttatttgtacaaaattcaaaaaagccatgagaaaaaaggaggtgcaactcattttat-3’) with 35 bp homology regions upstream and downstream of the gene, completely knocking out the ORF of the gene. The sequence of the ssODN allowed us to use no mutations to prevent accidental targeting by crRNAs.

#### Floxed *mec-4*

On the MSB943 strain (mScarlet(11)::*mec-4*), we first introduced a loxP site in intron 1 using crRNA #104 (5’ acgtaattttcaaattgcaa 3’) and ssODN agaagtacaagtgagtttacgtaattttcaaattgATAACTTCGTATAGCATACATTATACGAAGTTATcaacggtaaattttatttttgtatgcaattgtatt as homology repair template (loxP site in UPPERCASE). Once verified by sequencing (MSB1509[*mec-4(mir50mir117)*]), we introduced the second loxP site with crRNA #105 (5’ CACTGAATCAGAGGCTTATG 3’) and repair template 5’ gttgaattttgaaatgctcactgaatcagaggcttaCggagtgagtATAACTTCGTATAGCATACATTATACGAAGTTATtgaaaaatttaggcaaagaacgtgtatcaaagaaa 3’ (loxP site in UPPERCASE) with 1 point mutation (UPPERCASE C). We named the final allele *mec-4(mir50mir117mir118)* and generated strain MSB1514.

### Locomotion experiments

#### Cohort tracking

##### Imaging setup

Locomotion experiments were carried out in a home-built setup (Fig. 1 b), with a large-format sCMOS camera (Imaging Source, DMK38UX255) using Imaging Source acquisition software and the objective with adjustable magnification (Fujifilm Fujinon, CF25Za-1S). Before the experiment, the plates were transferred to the imaging chamber and left to settle for 30 min on NGM plates and 10 min on PAA gels. The recordings were carried out for 15 min on NGM plates and 10 min on PAA gels, unless otherwise stated, sampled at 20 Hz. The PAA gel recording was performed inside an additional isolated chamber with a water reservoir to avoid drying of the PAA gels. Brightfield illumination with white-light LED of intensity 3.7 μW/mm^2^ was used (measured in the worm plane) unless otherwise stated. In the tetracycline conditional rescue experiment, the dark-field red LED ring-shaped illumination has an intensity of 1.85 μW/mm^2^. In the optogenetic experiments with ACR1, dark-field illumination light with the intensity of 0.15 μW/mm^2^ for green, 519 nm, and 0.11 μW/mm^2^ for red, 629 nm was used. The power of the light was measured with a Thorlabs microscope slide power meter head (S170C) attached to the PM101A power meter console.

##### PAA gels

PAA substrates were prepared in different acrylamide/bisacrylamide ratios (as tabulated below), resulting in different stiffness moduli^65^. The PAA gels were obtained by mixing the solutions of PBS, acrylamide (40%), bisacrylamide (2%), ammonium persulfate (APS), and tetramethylethylenediamine (TEMED), as listed below. 500 μL of the mix was placed in the glass well plates, immediately covered with a coverslip, and left to polymerize for about an hour. The plates were stored at 4 °C dipped in PBS solution until use and used within a week. For the spontaneous locomotion assay, plates were seeded with 50 μL concentrated OP50 and left in the hood until dry.

**Table:** 

	Acrylamide (40%) (μL)	Bisacrylamide (2%) (μL)	PBS (μL)	APS (μL)	TEMED (μL)
0.5 kPa	50	7.5	437	2.5	0.25
1.25 kPa	68.75	12.5	416	2.5	0.25
6 kPa	93.75	15	388.5	2.5	0.25
30 kPa	93.75	80	323.5	2.5	0.25
100 kPa	150	110	227.25	2.5	0.25

##### Locomotion on tetracycline NGM plates

To block the self-cleavage of the tetracycline aptazyme, NGM agar containing different concentrations of tetracycline was prepared fresh before each experiment. The stock solution of tetracycline was distributed on the surface of the plates to reach the final concentrations of 2.5, 5, and 10 μM prior to OP50 seeding. The animals were synchronized, seeded, and left in a 20 °C incubator until they reached the late L4 stage. Then they were transferred to tetracycline plates and left for about 4.5–5 h. The locomotion experiments were performed as described earlier.

##### Data processing

The videos were processed with Tierpsy Tracker (1.5.1)^66^. The parameters were adjusted for each video; an example parameters file can be found in the supplementary material. The trajectories of the crossed worms were linked manually. The tracks were further processed with customized Python scripts, and then the violin plots with the average for each individual were plotted using the Seaborn package.

#### Single animal imaging on PAA substrates

Single animal imaging and tracking were performed as previously described^35^. Briefly, 3–5 min long videos of young adult synchronized animals transferred to non-seeded polyacrylamide gels (without bacteria as a food source) were recorded at 25 FPS using a home-built tracking platform. Imaging processing was performed using eigenworms with custom MATLAB scripts. To compare genotypes, the behavior of the locomotion of the animal was decomposed using the Eigenworm analysis^34^. To construct and visualize the 3D distributions of the modes (*a*_1_, *a*_2_, *a*_3_), the 3D kernel density estimate of the first three modes was calculated in R using the ks package^67^ with an unconstrained plug-in selector bandwidth. We choose to indicate the 10%, 25%, and 50% contours of the highest-density regions in the manifold and the 2D projections of the floating data cloud along the corresponding planes. To compare two different data sets and test for the null hypothesis that the two kernel density functions are similar, the local density between two distributions was tested using a binned kernel density estimator. For this, we used the kde.local.test function from the ks package in R^67^ to (a) convert all n data points of each distribution to counts on a 100 × 100 × 100 binning grid and embed into a matrix *C*, (b) evaluate the kernel function at these grid points to embed them into a matrix *K*, and (c) then obtain the binned density estimator *f* from a sequence of discrete convolutions of *C* and *K*. The exact formulation of that procedure and a detailed presentation of the algorithm can be found in ref. ^68^. For all grid points in which *p* > 0.01, we accept the null hypothesis that the two densities at this grid point for the two distributions are the same. For all other grid points, a polarity is assigned and plotted as two different colors in a 3D voxelgram (e.g., Fig. 2), indicating that *x*_1_ > *x*_2_ or *x*_1_ < *x*_2_.

### Calcium imaging in immobilized animals

#### Animal preparation

Animals were synchronized and grown in a 20 °C incubator until the YA stage. 6% agarose pads were prepared using physiological buffer (145 mM of NaCl, 2 mM of Ca_2_Cl, 1 mM of MgCl_2_, 5 mM of KCl, 644 10 mM Hepes, and 25 mM of Glucose, with a pH 7.4) in a glass slide. To each agar pad, 0.4 μL of OP50 and 1 μL of latex beads (Polybeads, 0.2 μm; Polysciences) were added to help to immobilize worms.

#### Imaging setup

Imaging the spontaneous activity of the TRNs in immobilized worms was done on a Leica DMi8 microscope with a ×40/1.1 water immersion lens, Lumencor Spectra X LED light source, fluorescence cube with beam splitter (Semrock Quadband FF409/493/573/652), a Hamamatsu Orca Flash 4 V4 sCMOS camera, and HCImage software (version 4.4.2.7). Cyan—488 nm and green—555 nm (both 8% of LED power) illuminations were used to excite the green and red fluorescence of TRN::GCaMP6s and tagRFP. Emission was split with a Hamamatsu Gemini W-View with a 538 nm edge dichroic (Semrock, FF528-FDi1-25-36) and collected through two single-band emission filters, 512/25 nm for GCaMP6s (Semrock, FF01-512/23-25) and 620/60 for mKate (Chroma, ET620/60m). Exposure times were set to 200 ms, with 2.5 FPS, and the recordings were carried out for 400 s (subjected to photobleaching).

#### Analysis

The location of the PLM neurons was manually indicated with a customized Python & OpenCV program. An area with a 20 pixel radius around the point was selected as the neuron body and averaged for further analysis, *F*_ROI_. Background fluorescence was defined for each frame as the average of all pixels below the 20th percentile *F*_bckg_, while worm fluorescence was defined as the average of all pixels between the 50th and 70th percentiles *F*_worm_. The neural activity was then approximated as

1 $$\Delta F/F=\frac{{F}_{{\rm {ROI}}}-{F}_{{\rm {bckg}}}}{{F}_{{\rm {worm}}}-{F}_{{\rm {bckg}}}}.$$

### Calcium imaging in freely moving animals

#### Imaging setup

Imaging was performed using an in-home built dual-camera upright epifluorescent microscope, using a Nikon CFI S Plan Fluor LWD 20XC 0.7NA objective lens, with two Hamamatsu ORCA-Fusion cameras C14440-20UP, Lumencore LED Light source Spectra X, with cyan LED, 475/28 nm, at a final light intensity of up to 47.5 mW at the sample plane, and green LED, 555/28 nm, at the final light intensity of up to 83 mW at the sample plane, measured with a Thorlabs microscope slide power meterhead (S170C) attached to PM101A power meter console. The hardware was configured in master–slave configuration, with one camera functioning as a master, sending a TTL pulse to the second camera and the LED light source. This helped minimize the phototoxicity. In the epifluorescence path, the dichroic filter AHF FF493/574-Di01-25 × 36 was used. Emitted fluorescence was then split into two cameras using a dichroic beamsplitter AHF T 560 LPXR and filters ET510/20m and ET620/60nm (both Chroma, commercially purchased from AHF) for GCaMP6s and TagRFP, respectively. The stage Märzhäuser Wetzlar SCANplus IM 130 × 85 S/No. 17120519 was used together with the TANGO 2 Desktop controller Art. No.: 31-76-150-1802. Z-stage controller PI C-863.12 C-863 Mercury Servo Controller was used for vertical adjustments.

#### Automatic worm tracking, Napari-based plugin Track-live

We implemented Track-live plugin in Napari^69^ using Napari multithreading to (1) manage the communication between two cameras, (2) reading and manipulating the *XY* stage position to keep the worm within the field of view, (3) live display of the videos that were being acquired, and (4) allowing a user to interact with the interface (e.g. terminate the acquisition earlier, switch to manual *XY* tracking). The LED and *XY* stage were controlled through Micromanager^70^ and the former also through the LumencorCIA plugin, and were further passed through the Pycromanager^71^ communication bridge to Track-live. The cameras were controlled through Python Hamamatsu SDK. After read-out from the camera, the images were binned twice for further processing and analysis. The live image analysis, enabling XY stage adjustments to follow the worm, relied on finding the centroid position of the binary mask obtained by thresholding the blurred auto-fluorescence image from a ‘red-filter camera’. The threshold was set to the 90th percentile value from the image when the user pressed the tracking button, ensuring that the worm is within the field of view. *Z* adjustments were done manually. Exposure time was set to 20 ms, ensuring a reasonable trade-off between image quality and blurring as the worm moves. The videos were acquired for 2 min, at a sampling frequency of 8 FPS. The code is available at https://gitlab.icfo.net/NMSB/track-live-close-loop.

#### Video processing

##### Neural tracking and neural activity

The cell bodies of the TRNs were identified within each frame, and their trajectories were linked using a simple LAP tracker implemented in the TrackMate Fiji plugin^72^. The trajectories were then manually curated and cleaned. Each neuron was then manually assigned to one of the classes AVM, ALM, PVM, or PLM based on the location. In order to approximate neural activity, circular areas around the cell body (with radius 10 pix, which corresponds to 32 μm) were cropped in each frame, averaged over the ROIs for the green and red channels separately, *F*_ROI_. For each channel, background fluorescence was defined for each frame as the average of all pixels below the 20th percentile *F*_bckg_, while worm fluorescence was defined as the average of all pixels between the 50th and 70th percentiles *F*_worm_. The fluorescence intensity was estimated as

2 $${F}_{i}=\frac{{F}_{{\rm {ROI}}}-{F}_{{\rm {bckg}}}}{{F}_{{\rm {worm}}}-{F}_{{\rm {bckg}}}},$$

for both channels, *i* ∈ {GCam*p*, TagRFP}. The neural activity was then approximated as the ratio of the ‘green’ and ‘red’ fluorescence values *r* = *F*_GCamp_/*F*_TagRFP_.

##### Skeletonisation

In the first step, each frame from the red channel was denoised using the fastNlMeansDenoising function from the Open-cv Python package, and then thresholded using cv2.threshold and a manually selected optimal threshold. The binary masks obtained were then dilated using the cv2.dilate function to fill in the holes in the binary mask. Then all contours found with cv2.findContours function, with the area that reached 10.000 pixels or more, were kept as potential worm candidates and otherwise ignored. The edges of the masks were smoothed using the sequence of functions cv2.GaussianBlur, skimage.exposure.rescale_intensity, and then cv2.threshold. The binary masks were then skeletonized cv2.ximgproc.thinning, fil_finder pipeline, which included preprocess_image, create_mask, medskel, analyze_skeletons functions. The skeleton was then identified as the longest path found and interpolated to 50 points using cubic spline interpolation. Frames, where the skeletons were shorter than 750 pixels (~490 μm) or longer than 130% of the average, were considered unreliable and removed from further analysis. Skeleton finder is not sensitive to worm orientation, which can lead to systematic flips in the head-tail orientations. In order to account for that, the skeletons were then oriented from head to tail by minimizing the Cartesian distances head-head and tail-tail between the consecutive frames, and by using the neuron positions within the body, e.g., PLM is located in the tail, while ALM/AVMs are located in the anterior body.

#### Time-series analysis

For each worm, each frame, and for each neuron, tangential and normal directions were found using the skeleton point closest to the cell body. The velocity was then decomposed into tangential and normal components, *v*_t_ and *v*_n_, respectively. In further analysis, 5-s windows were considered. For each of them, the cross-covariance and cross-covariance coefficient between the ratio *r* and the tangential velocity *v*_t_ were estimated using scipy.signal.correlate, mean-subtracted signals, and ‘valid’ modality of the cross-covariance function scipy.signals.correlate (*r*, *v*, mode = 'valid'), where *r* (1st argument) is shorter, 5 s and *v* (2nd argument) is longer 10 s, and their middle points correspond to the same point in time. The maxima of the cross-covariance, including both the peak value and its corresponding time lag *τ*, were grouped and smoothed using a kernel density estimator (seaborn.kdeplot). Per convention, we assigned positive time lags when the velocity precedes TRN activity, and negative lags when the TRN activity precedes reversals or increases in velocity (see also Supplementary Fig. 8). Next, we statistically verified whether the peaks originate from concurrent activity in both time series of calcium and velocity, or occur rather as inner properties of each time series. For details, see the section “Surrogate testing” and Supplementary Fig. 9. The codes for the sections “Video processing” and “Time-series analysis” are available under the link: https://gitlab.icfo.net/NMSB/cal-free-m-worm.

### Optogenetics

#### TRNs killing using PH-miniSOG

In order to obtain acute killing of the touch cells, a strain expressing mini Singlet Oxygen Generator in the TRNs was used, CZ23281^73^. MiniSOG generates the reactive oxygen species (ROS), singlet oxygen, after illumination with blue light. Illumination of neurons expressing miniSOG targeted to the membrane (PH-miniSOG) causes neuronal death. The extrachromosomal worms were exposed to blue light 470 nm of the intensity 1.7 mW/mm^2^ (Mightex, Series Light Source Control Module) for 4 min around 18 h before the locomotion experiment. The light was mounted on a specially designed box, 10 cm above the surface of the plate, which ensured the uniform illumination of the whole plate. After the illumination, the worms were kept in the darkness until the locomotion experiment.

#### Stimulation of the touch receptor neurons with ChR2

In order to investigate if the lethargic phenotype can be rescued by chronic stimulation of the Touch Receptor Neurons, we crossed strains AQ2334 and GN693 to generate MSB990, expressing channelrhodopsin (ChR2(H134R)) under the control of the* mec-4p* promoter in the *mec-4(u253)* mutant background. L1 worms were seeded on plates with 100 μL of OP50 mixed with ATR to have a final concentration on the plate of 0.1 mM prepared as described in ref. ^74^. The animals were then stimulated with 455 nm LED (Osram Oslon power cluster; ILR-ON16-DEBL-SC211-WIR200) light powered by R&S NGE100B Power supply series. The power supply was controlled by customized Python (v3) code, to generate the Poisson-like light pattern with an average interval between pulses of 5 min, each pulse lasted 3 s. The worms were stimulated for about 50 h, transferred to plates without ATR, and assayed in the locomotion setup using dark field ring-shaped red light illumination (as described earlier) about 2 h later.

#### Silencing of the touch receptor neurons with ACR1

MSB1244 worms expressing *mec-4*p::cGAL4 and UAS::ACR1 were obtained by crossing the previously described MSB952 strain^64^ to MSB927 (*mirEx238*[*mec-4*p::cGAL4+*myo-2*p:mCherry]). Plates with ATR were prepared as specified in the previous section, and MSB1244 L4 worms were seeded on them. After 16h incubation at 20 °C, animal were recorded for 15 min and analyzed as described.

### Calcium imaging in the microfluidic body wall touch device

#### Preparation of the mold using dry etching

The mold was fabricated by Reactive Ion Etching (RIE) on a silicon master wafer. Deep RIE using the Bosch process is a microfabrication technique that allows etching silicon substrates and obtaining high aspect ratio features, such as the one used in this work, where microchannels of around 50 μm height were separated by actuators of 10 µm, which required narrow deep trenches with an aspect ratio of 5:1 in the mold. This method consisted of alternating steps of etching and passivation that provided highly selective etching and resulted in well-defined and vertical walls.

Before etching, the desired design was patterned on 4-inch silicon wafer (525  ± 25 μm, MicroChemicals, GmbH) as a resist mask by conventional photolithography. Briefly, a 22 μm layer of negative resin AZ-125NXT (AZ Electronic Materials GmbH) was spun on the wafer at 3000 rpm for 20 s (SPIN150x Spin coater, Polos-SPS Europe B.V.) and baked for 15 min at 125 ^∘^C. Then, the body-touch design was exposed using a maskless aligner (MLA150, Heidelberg Instruments) with a UV-light (375 nm wavelength) dose of 900 mJ/cm^2^, developed by stirring in AZ 726 MIF developer (AZ Electronic Materials GmbH) for 8 min, and rinsed in DI water. Using this mask to protect the silicon below, the Bosch process was performed in a RIE system with Inductively Coupled Plasma (SI 500 ICP-RIE Plasma System, Sentech Instruments GmbH). For the etching steps, SF6 was used at an ICP power of 950 W, an etching RF power of 25 W, and a gas flow of 129.9 sccm, with an etching time of 3 s. For the passivation steps, C4F8 was used at an ICP power of 800 W, a passivation RF power of 5 W and a gas flow of 80 sccm, with a passivation time of 2 s. The substrate was kept at a constant temperature of 10 ^∘^C. These steps were repeated for 200 cycles to achieve a total depth (resin and etched silicon) of around 50 μm.

#### Device preparation

The devices were replica-molded as previously described^8^. In short, the devices were prepared by casting a mix of PDMS base and curing agent (Sylgard™ 184, Dow) in a 10:1 proportion on the mold and baking it for 2 h at 70 ^∘^C. To facilitate peeling the PDMS, the wafer was previously silanized with chlorotrimethylsilane (Merck KGaA) for 30 min. After carefully peeling the PDMS, the individual devices were cut, and 0.75 mm inlet and outlet perforations were created by a pneumatic puncher. Finally, the patterned PDMS and glass coverslides (#1.5, VWR) were irreversibly bonded by oxygen plasma activation. Diaphragm deflection versus pressure was previously calibrated^75^.

#### Animal loading into a microfluidic trap

Loading of the animals in the body wall chip was performed as described in detail elsewhere^76^. For imaging PDE in control and *mec-4* conditions, we used MSB661 and MSB636, respectively. For imaging DVA neuronal activity secondary to direct mechanical stimulation of the body wall, we used MSB273 and MSB1410 in the control and *mec-4* mutant background, respectively. Briefly, 2–3 young adult animals were transferred to an M9-filtered droplet. The worms were then aspirated through an SC23/8 gauge metal tube (Phymep) connected to a 3 ml syringe (Henke Sass Wolf) with a PE tube (McMaster-Carr). Once the tube was inserted into the chip inlet, the animals were loaded into the waiting chamber by applying gentle pressure with the syringe.

#### Calcium imaging and mechanical stimulation

In vivo calcium imaging of TRNs was performed by positioning the worm-loaded microfluidic device in a Leica DMi8 microscope with a 40x/1.1 water immersion lens, Lumencor Spectra X LED light source, fluorescence cube with beam splitter (Semrock Quadband FF409/493/573/652), a Hamamatsu Orca Flash 4 V3 sCMOS camera, and HCImage software (version 4.4.2.7). Cyan-488 nm (≈6.9 mW) and yellow-575  nm ( ≈ 12.6 mW) illuminations were used to excite the green and red fluorescence of TRN::GCaMP6s and mtagRFP, which was used to correct possible artifacts of animal movement and identification of TRN. The incident power of the excitation light was measured with a Thorlabs microscope slide power meter head (S170C) attached to the PM101A power meter console. Emission was split with a Hamamatsu Gemini W-View with a 538 nm edge dichroic (Semrock, FF528-FDi1-25-36) and collected through two single-band emission filters, 512/525 nm for GCaMP6s (Semrock, FF01-512/23-25) and 620/60 for mKate (Chroma, ET620/60m). The emission spectra were split by the image splitter, allowing different exposure times for each signal. For the application of mechanical loads to the animal’s body wall, the stimulation channel was connected to a piezoelectric pressure controller (OB1-MK3, Elveflow) as described^74^. To follow calcium transients, videos were taken at 10 frames-per-second with 80 ms exposure time, using the master pulse of the camera. The camera SMA trigger out was used to synchronize the stimulation protocol in the Elveflow sequencer, which consisted of three stimulations, 10 or 20 s pre-stimulation, 2 s stimulation (2500 mbar buzz). Each stimulus was separated by 20 s.

#### Calcium analysis

Images were processed using ImageJ, and intensity signals were extracted as described^74^. First, the neuron of interest was manually labeled on the basis of the calcium-insensitive channel tagRFP. The position was automatically tracked in the following frames and used to extract the GCaMP intensity. A smooth filter (moving average filter) was applied. The calcium-sensitive signal was background subtracted and then normalized to the first 100 frames pre-stimulation ((*F*−*F*_0_)/*F*_0_). After arithmetic averaging all recordings, the *t*-test statistics for each timepoint in the timeseries was calculated according to $t=\frac{{\mu }_{1}-{\mu }_{0}}{{s}_{1}\sqrt{{n}_{1}}+{s}_{0}\sqrt{{n}_{0}}}$ where *μ*_1_ and *μ*_0_ are the mean values of each group at time *t*, *s*_1_ and *s*_0_ are the standard deviations for each group at time *t* and *n* equals the number of recording for each group. The *t*-test statistic was then used to calculate the *p*-value considering the degrees of freedom *n*_1_ + *n*_0_−2, and the null hypothesis was rejected if the absolute value of *t* was larger than its critical value at the level of significance *α *= 0.01. All operations were performed in R (version 4.2.2, 2022-10-31) using custom routines.

### Traction force microscopy

#### Polyacrylamide gels preparation

In order to simplify the imaging, reduce the potential artifacts, and ensure the estimation of the tractions on the surface of the gels, we assured placement of the fluorescent beads on the surface of polyacrylamide (PAA) gels. The gels were prepared following the protocol from ref. ^77^, unless stated otherwise. In short, the cover-slip glasses were functionalized with poly-L-lysine, air-dried, coated with fluoSpheres 0.2 μm carboxylated beads (Fisher, F8809, Ex: 540 nm. Em: 560 nm) diluted with miliQ H_2_O in a ratio of 1:2000, and sonicated for 30 min. Polyacrylamide gels were prepared in three different concentration ratios of bisacrylamide and acrylamide, as described above. The spectral properties of the beads were chosen in order to minimize the effect of the excitation light on the worm’s behavior.

#### Experimental setup

The image processing pipeline has been developed in a way that allows the use of various setups, without the need for a dual-view camera, as long as segmentation of the worm silhouette is possible from the videos. The simultaneous stage position acquisition is not necessary, which is a caveat when using most of the commercially available image acquisition software, and making a manual worm is a feasible option. The stage position movement is estimated from the background image matching between the consecutive frames. For the best information content, each frame should include a full image of a worm and a sufficiently dense bead image underneath. Imaging was performed as described above for freely moving worms, using a Nikon 20X 0.7 objective lens and the green 555/28 nm LED of the Lumencor lightsource. Images were acquired at 8Hz, with an exposure time of 20ms, and bin 2.

#### Image preprocessing

Each frame was divided into three parts, (1) background ‘far away’ from the worm, where the beads image is unaffected by traction forces exerted by the worm, which can be used to estimate offset stage movement; (2) worm silhouette that can be used to find the behavioral characteristics of the worm, such as body curvature; and finally, 3) background region in the close vicinity of the worm empirically chosen to be around 1.5 worm width, where the beads image is affected by the traction forces exerted by moving worm.

#### Worm segmentation

The worm videos were first segmented to find the worm silhouette, using the tracker annotator from ‘Segment Anything for Microscopy’ napari plugin^78^ using the ViT-L model. The parameters for each video were chosen individually, based on the movement of the worm, to maximize the automatic segmentation of the worm. The incorrectly segmented frames were further manually curated, and the worm images were kept for further analysis. The worm masks were further processed as described in the section “Video processing”.

#### Offset correction

‘Far-away’ background regions were further used to estimate offset stage movement in pairs of consecutive frames using subpixel maxima in cross-correlation landscapes, implemented in openpiv packages and self-prepared scripts.

#### Particle image velocimetry

The pairs of consecutive frames, containing regions of the image in the close vicinity of the worm, were used to estimate particle image velocimetry, using the openpiv package^79^. The parameters chosen were 42 pixels of window size, with 43 search windows, and 60% overlap, and a pixel size of 0.65 μm. The PIV in the worm and in the background regions was set to zero, potentially irrelevant for the analysis.

#### Traction estimation

The traction forces were estimated using the pyTFM package^80^, which uses the Fourier transform to estimate the traction fields^81^. The parameters used: Poisson ratio of the substrate, *σ* = 0.457, height of the substrate, *h* = 1 mm, and Young’s modulus of the substrate 1.25, 6, or 30 kPa.

#### Traction quantification and behavioral analysis

The profiles of the traction fields along the body of the worm were further estimated, using geometrical image transformations: that is, the images of the traction fields were straightened along the skeletons. The images were processed using self-prepared functions and map coordinates from scipy.ndimage package. The profiles were obtained separately for magnitudes, tangential, and normal components with respect to the skeleton midlines. For each profile, for each body point, the 97.5 percentile of the absolute value of the array was selected, and later was assigned back its sign, in order to preserve the negative values. For the time point and for each midline of the skeleton, the body angle was found and normalized between−*π* and *π*.

All codes can be found under the link: https://gitlab.icfo.net/NMSB/traction-force-worm.

#### Statistical analysis of the traction profiles

To account for repeated measurements along the body of each worm, tractions were modeled using a linear mixed-effects model implemented with the lme4 package in R (version 4.2.2, 2022-10-31). Traction was the dependent variable, with *strain* and *body.position* (normalized from head to tail) included as fixed effects, along with their interaction (*strain*  × *body.position*) to capture region-specific differences between strains. Random intercepts were included for each worm to account for multiple body points measured per individual. The model can be expressed as:

$${{{\rm{tractions}}}}_{ij}=	 {\beta }_{0}+{\beta }_{1}{{{\rm{strain}}}}_{i}+{\beta }_{2}{{{\rm{body.position}}}}_{j} \\ 	+{\beta }_{3}({{{\rm{strain}}}}_{i}\times {{{\rm{body.position}}}}_{j})+{u}_{{{{\rm{worm}}}}}+{\epsilon }_{ij}$$

where ${u}_{{{{\rm{worm}}}}} \sim {{{\mathcal{N}}}}(0,{\sigma }_{u}^{2})$ represents worm-specific random effects and ${\epsilon }_{ij} \sim {{{\mathcal{N}}}}(0,{\sigma }^{2})$ is the residual error. Statistical significance of fixed effects was assessed using Type III ANOVA. Denominator degrees of freedom were estimated using Satterthwaite’s approximation to account for uncertainty in variance component estimation within the mixed-effects framework. Post-hoc comparisons along the body axis were performed using estimated marginal means with multiple-testing correction where appropriate.

### Statistics and reproducibility

Statistical modeling and hypothesis testing were performed in MATLAB (v2019) and R (version 4.2.2, 2022-10-31). No statistical methods were used to predetermine the sample sizes, but our sample sizes are similar to those reported in previous publications. Data distributions were assumed to be normal, unless obviously not, but this was not formally tested. All datasets were acquired in a randomized fashion, and when they were not, the data were not biased by the history. Some data were not collected blind to the genotype and analyzed blind to the experimental condition (Fig. 1).

#### Multilevel analysis

For multilevel hierarchical analysis, data did not meet the assumptions of normality (Fig. 1), as indicated by the results of the Shapiro-Wilk test. Consequently, a mixed-effect linear model^82^ was employed as an alternative to the adjusted t-test, for hierarchical data structures, where indicated. The average from individual worms is at the first level, and different plates as a second level. The computations were performed using the statsmodels Python module. A linear mixed-effects model can be expressed as

3 $$y=X\beta+Zb+\epsilon,$$

where *y* is the observed data. In our case, *y* is the statistic estimated for each individual worm. *Xβ* term accounts for fixed effects, *β*_0_ corresponds to baseline value in the control group, while *β*_1_ accounts for the difference between the groups. *X* contains information about the experimental conditions at the highest level (e.g., group affiliation of each worm). *Zb* term accounts for random effects, *b* = *b*_*x*_, *x* ∈ {plate_1_, . . . , plate_*n*_}, and *Z* contains information about the experimental conditions at the lower level (e.g., plate affiliation of each worm). *ϵ* corresponds to residual error. *β*, *b*, plate-level variability, and individual worm variability are determined by maximizing the restricted likelihood. *β*_1_ stores the information about the difference between group means. *P*-value for the fixed effect is then obtained by comparing *β*_1_ to its standard error and using a *t*-test. The *t*-test assesses whether the group difference, *β*_1_, significantly deviates from zero.

*Touch response to tetracycline dose*: The analysis was performed in R (version 4.2.2, 2022-10-31) using the lme4 package. Binary “yes/no" responses to mechanical touch were analyzed using a mixed-effects logistic regression to account for repeated trials within individual animals, with tetracycline dose as a fixed effect and individual animals as random intercepts to account for repeated touches on the same subject. Let *Y*_*ij*_ denote the binary response (0/1) for trial *j* from animal *i*, with response probability *p*_*ij*_ = Pr(*Y*_*ij*_ = 1). We assumed

$${Y}_{ij} \sim {{{\rm{Bernoulli}}}}({p}_{ij})$$

and modeled the log-odds of response using a logit link function:

$${{{\rm{logit}}}}({p}_{ij})=\log \left(\frac{{p}_{ij}}{1-{p}_{ij}}\right)={\beta }_{0}+{\sum}_{k=1}^{K-1}{\beta }_{k}{X}_{k,ij}+{b}_{i}$$

where *β*_0_ is the fixed intercept, *X*_*k*,*ij*_ are indicator variables encoding the experimental conditions (with one condition serving as the reference level), and *β*_*k*_ are the corresponding fixed-effect coefficients. The term *b*_*i*_ represents a random intercept for animal *i*, accounting for within-animal correlation across repeated trials.

Random effects were assumed to be normally distributed:

$${b}_{i} \sim {{{\mathcal{N}}}}(0,{\sigma }_{{{{\rm{animal}}}}}^{2})$$

where *b*_0*i*_ represents animal-specific baseline variation and *b*_*ki*_ represents animal-specific deviations in the effect of condition. Random effects were assumed to be normally distributed.

#### Surrogate testing

To statistically validate the cross-covariance between neuronal activity and velocity, we applied intersubject surrogate testing alongside a control group^83,84^. Specifically, to differentiate genuine concurrent activity (*H*_1_) from coincidental cross-covariance peaks arising due to the intrinsic properties of each time series (*H*_0_), we computed the cross-covariance between the neuronal activity of one worm and the velocity of another worm within the same group. This process was repeated for all possible pairings. The 2.5th and 97.5th percentiles of the surrogate cross-covariance peak values were used as reference thresholds corresponding to a *p*-value of 0.05. If the observed cross-covariance exceeded the 97.5th percentile, *H*_0_ could be rejected at the *α *= 0.05 significance level, indicating that the peaks likely result from genuine concurrent activity in calcium dynamics and tangential velocity.

#### Calculation of the effect size

To quantify the effect of the genetic and pharmacological perturbation on animal locomotion, we resorted to estimation statistics of non-normally distributed data. Cliff’s delta (*δ*) is a non-parametric measure of effect size that quantifies the degree of difference between two independent distributions. It is defined as the probability that a randomly chosen observation from one group is larger than a randomly chosen observation from the other group, minus the reverse probability:

δ=P(X>Y)−P(Y>X)

where *X* and *Y* are values from the two groups.

For every pair of observations (*x*_*i*_, *y*_*j*_), assign:

$$\,{{\rm{score}}}\,=\left\{\begin{array}{ll}+1\quad &\,{{\rm{if}}}\,{x}_{i} > {y}_{j},\\ -1\quad &\,{{\rm{if}}}\,{x}_{i} < {y}_{j},\\ 0\quad &\,{{\rm{if}}}\,{x}_{i}={y}_{j}.\end{array}\right.$$

All scores were summed across all pairs and divided by the total number of comparisons (*n*_*X*_ × *n*_*Y*_) to obtain *δ*:

$$\delta=\frac{\mathop{\sum }_{i=1}^{{n}_{X}}\mathop{\sum }_{j=1}^{{n}_{Y}}\,{{\rm{sign}}}\,({x}_{i}-{y}_{j})}{{n}_{X}\times {n}_{Y}}.$$

The value of *δ* ranges from −1 (all values in group *Y* are higher than *X*) to +1 (all values in group *X* are higher than *Y*), with 0 indicating no difference. Calculations were done using the dabestr library in R (version 4.2.2, 2022-10-31)^85^.

*Inference*: Bootstrapped 95% bias-corrected and accelerated (BCa) confidence intervals were computed from 5000 resamples of the data to quantify the uncertainty in *δ*.

### Reporting summary

Further information on research design is available in the Nature Portfolio Reporting Summary linked to this article.

## Supplementary information

Supplementary Information

Peer Review file

Description of Additional Supplementary Files

Supplementary Movie 1

Supplementary Movie 2

Supplementary Movie 3

Supplementary Movie 4

Supplementary Movie 5

Supplementary Movie 6

Supplementary Movie 7

Supplementary Movie 8

Supplementary Movie 9

Reporting Summary

## Source data

Source Data

## Data Availability

All numerical source data are presented in the supplementary material of the manuscript and available from the corresponding author. In addition, the curvature matrices (10.5281/zenodo.18153808) and all numerical source data (10.5281/zenodo.20340865) were deposited on zenodo.org. All other raw data is available from the corresponding author on request. Source data are provided with this paper.

## Code availability

The codes are deposited on: https://gitlab.icfo.net/NMSB/traction-force-worm, https://gitlab.icfo.net/NMSB/track-live-close-loop, https://gitlab.icfo.net/NMSB/net-sim-worm, and https://gitlab.icfo.net/NMSB/cal-free-m-worm.
