# Supplementary material for: Mechanosensory encoding of surface mechanics optimizes locomotion
Source: Nat Commun. 2026 Jul 25;17:9083. doi: 10.1038/s41467-026-75352-7 (PMC13518797; doi:10.1038/s41467-026-75352-7)
Supplement: Supplementary file 1 [file 41467_2026_75352_MOESM13_ESM.pdf]

nature portfolio

Corresponding author(s): Michael Krieg

Last updated by author(s): May 20, 2026

Reporting Summary

Nature Portfolio wishes to improve the reproducibility of the work that we publish. This form provides structure for consistency and transparency in reporting. For further information on Nature Portfolio policies, see our Editorial Policies and the Editorial Policy Checklist.

Statistics

For all statistical analyses, confirm that the following items are present in the figure legend, table legend, main text, or Methods section.

n/a	Confirmed	
☐	☒	The exact sample size (*n*) for each experimental group/condition, given as a discrete number and unit of measurement
☐	☒	A statement on whether measurements were taken from distinct samples or whether the same sample was measured repeatedly
☐	☒	The statistical test(s) used AND whether they are one- or two-sided<br>*Only common tests should be described solely by name; describe more complex techniques in the Methods section.*
☒	☐	A description of all covariates tested
☐	☒	A description of any assumptions or corrections, such as tests of normality and adjustment for multiple comparisons
☐	☒	A full description of the statistical parameters including central tendency (e.g. means) or other basic estimates (e.g. regression coefficient) AND variation (e.g. standard deviation) or associated estimates of uncertainty (e.g. confidence intervals)
☐	☒	For null hypothesis testing, the test statistic (e.g. *F*, *t*, *r*) with confidence intervals, effect sizes, degrees of freedom and *P* value noted<br>*Give P values as exact values whenever suitable.*
☒	☐	For Bayesian analysis, information on the choice of priors and Markov chain Monte Carlo settings
☐	☒	For hierarchical and complex designs, identification of the appropriate level for tests and full reporting of outcomes
☐	☒	Estimates of effect sizes (e.g. Cohen's *d*, Pearson's *r*), indicating how they were calculated

Our web collection on statistics for biologists contains articles on many of the points above.

Software and code

Policy information about availability of computer code

Data collection	HCImage (version 4.4.2.7), Micromanager customised Python (version 3) software available on https://gitlab.icfo.net/nmsb/track-live-close-loop,<br>simulation data, matlab (version R2019b) code: https://gitlab.icfo.net/nmsb/net-sim-worm<br>Mechanical Inverse Analysis, matlab code: https://www.dropbox.com/scl/fo/tvc7nwcfso134207ks2zj/APVytHIzGrNNUNANflp4FBc?rlkey=0g0tgad2jv8h1yggo190woq6o&dl=0
Data analysis	Tierpsy Tracker (1.5.1), Napari MicroSAM (v 1.1.1), TrackMate Fiji plugin, own Python (version 3) software: https://gitlab.icfo.net/nmsb/cal-free-m-worm, https://gitlab.icfo.net/nmsb/traction-force-worm<br>R scripts (version 4.2.2, 2022-10-31)

For manuscripts utilizing custom algorithms or software that are central to the research but not yet described in published literature, software must be made available to editors and reviewers. We strongly encourage code deposition in a community repository (e.g. GitHub). See the Nature Portfolio guidelines for submitting code & software for further information.

Data

Policy information about availability of data

All manuscripts must include a data availability statement. This statement should provide the following information, where applicable:

- Accession codes, unique identifiers, or web links for publicly available datasets
- A description of any restrictions on data availability
- For clinical datasets or third party data, please ensure that the statement adheres to our policy

All numerical source data is presented in the supplementary material of the manuscript and available from the corresponding author. The curvature matrices were deposited on zenodo.org (doi: 10.5281/zenodo.18153808). All other raw data is available from the corresponding author on request.

\section*{Code availability:} The codes are deposited on: \\
\url{https://gitlab.icfo.net/NMSB/traction-force-worm}\\
\url{https://gitlab.icfo.net/NMSB/track-live-close-loop}\\
\url{https://gitlab.icfo.net/NMSB/net-sim-worm}\\
\url{https://gitlab.icfo.net/NMSB/cal-free-m-worm}

Research involving human participants, their data, or biological material

Policy information about studies with human participants or human data. See also policy information about sex, gender (identity/presentation), and sexual orientation and race, ethnicity and racism.

Reporting on sex and gender	does not apply
Reporting on race, ethnicity, or other socially relevant groupings	does not apply
Population characteristics	does not apply
Recruitment	does not apply
Ethics oversight	does not apply

Note that full information on the approval of the study protocol must also be provided in the manuscript.

Field-specific reporting

Please select the one below that is the best fit for your research. If you are not sure, read the appropriate sections before making your selection.

☒ Life sciences ☐ Behavioural & social sciences ☐ Ecological, evolutionary & environmental sciences

For a reference copy of the document with all sections, see nature.com/documents/nr-reporting-summary-flat.pdf

Life sciences study design

All studies must disclose on these points even when the disclosure is negative.

Sample size	No formal sample size calculation was performed. Sample sizes were chosen based on previous studies in the field, empirical experience with the variability of the assays, and practical considerations related to experimental throughput, analytical burden and experimental cost. The selected sample sizes were sufficient to reproducibly detect biologically meaningful effects and yielded statistically significant differences where reported. All sample size are provided in the figures, captions, or supplementary tables.
Data exclusions	No data was excluded unless otherwise described in the manuscript.
Replication	All measurement are replicable and all attempts are described in the figures, figure captions or supplementary tables.
Randomization	The experimental and control groups were recorded in random order.
Blinding	All the automatic analysis is blind to a phenotype. Behavioral touch tests was peformed blind to a phenotype

Reporting for specific materials, systems and methods

We require information from authors about some types of materials, experimental systems and methods used in many studies. Here, indicate whether each material, system or method listed is relevant to your study. If you are not sure if a list item applies to your research, read the appropriate section before selecting a response.

Materials & experimental systems

n/a	Involved in the study
☒	☐ Antibodies
☒	☐ Eukaryotic cell lines
☒	☐ Palaeontology and archaeology
☐	☒ Animals and other organisms
☒	☐ Clinical data
☒	☐ Dual use research of concern
☒	☐ Plants

Methods

n/a	Involved in the study
☒	☐ ChIP-seq
☒	☐ Flow cytometry
☒	☐ MRI-based neuroimaging

Animals and other research organisms

Policy information about studies involving animals; ARRIVE guidelines recommended for reporting animal research, and Sex and Gender in Research

Laboratory animals	Caenorhabditis elegans in young adult stage N2 and derivatives (listed in Supplementary Table 2)
Wild animals	does not apply
Reporting on sex	Only hermaphrodites were used in the study unless otherwise stated (Extended Data Fig 1)
Field-collected samples	does not apply
Ethics oversight	C. elegans is a nematode and thus exempt from ethical approval and regulations.

Note that full information on the approval of the study protocol must also be provided in the manuscript.

Plants

Seed stocks	does not apply
Novel plant genotypes	does not apply
Authentication	does not apply
