# Supplementary material for: Mechanosensory encoding of surface mechanics optimizes locomotion
Source: Nat Commun. 2026 Jul 25;17:9083. doi: 10.1038/s41467-026-75352-7 (PMC13518797; doi:10.1038/s41467-026-75352-7)
Supplement: Supplementary file 2 [file 41467_2026_75352_MOESM1_ESM.pdf]

Supplementary Material for: Mechanosensory encoding of surface mechanics optimizes locomotion

Authors:

Aleksandra Pidde1, Montserrat Porta-de-la-Riva1, Costanza Agazzi1, Carmen Martínez-Fernández1, Alice Lorrach1, Ashutosh Bijalwan3, Neus Sanfeliu-Cerdán1, Alba Calatayud-Sanchez1, Eric Torralba-Sales2, Ravi Das1, José J. Muñoz2,3,4, Michael Krieg1,5

Affiliations:

(1) ICFO - Institut de Ciències Fotòniques, Castelldefels, The Barcelona Institute of Science and Technology, Barcelona, Spain;
(2) Universitat Politècnica de Catalunya (UPC), Laboratori de Càlcul Numèric (LaCàN), Dept. Mathematics, Esc. Eng. Barcelona Est (EEBE), Barcelona, Spain;
(3) Centre Internacional de Mètodes Numèrics en Enginyeria (CIMNE), Barcelona, Spain;
(4) Inst. de Matemàtiques de la UPC- BarcelonaTech (IMTech), Barcelona, Spain
(5) correspondence to: michael.krieg@icfo.eu

Contents

1 Supplementary Figures **2**

2 Supplementary Discussion **15**
- 2.1 Mathematical modeling of neural network and behavioral proxy 15
- 2.2 Details of the worm locomotion model 16
- 2.3 Inverse analysis through Optimal Control Theory 18
- 2.4 Notes on the time-series analysis of calcium activity and tangential locomotion 20
- 2.5 Additional discussion about parameter estimation from traction force microscopy 21

3 Supplementary Tables **23**

1 Supplementary Figures

Supplementary Figure 1

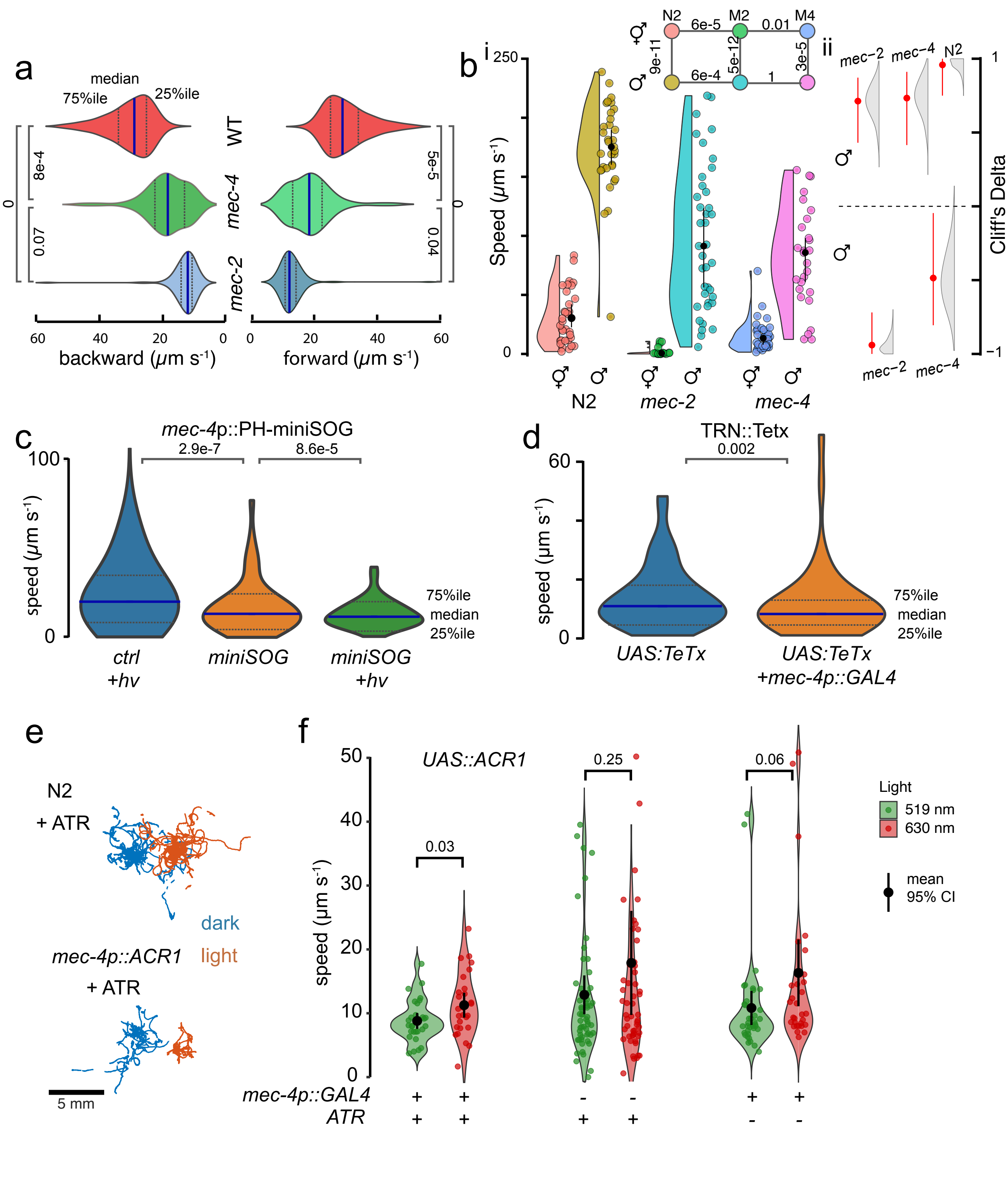

a
median
75%ile
25%ile
WT
mec-4
mec-2
8e-4
0.07
0
5e-5
0.04
0
backward (μm s-1)
forward (μm s-1)
b
i
ii
N2
M2
M4
6e-5
0.01
9e-11
5e-12
3e-5
6e-4
1
Speed (μm s-1)
250
0
N2
mec-2
mec-4
Cliff's Delta
1
−1
c
mec-4p::PH-miniSOG
2.9e-7
8.6e-5
speed (μm s-1)
100
0
ctrl +hv
miniSOG
miniSOG +hv
75%ile
median
25%ile
d
TRN::Tetx
0.002
60
0
UAS:TeTx
UAS:TeTx +mec-4p::GAL4
e
N2 + ATR
dark
light
mec-4p::ACR1 + ATR
5 mm
f
UAS::ACR1
0.03
0.25
0.06
speed (μm s-1)
Light
519 nm
630 nm
mean
95% CI
mec-4p::GAL4
ATR

Supplementary Fig. 1 *(preceding page).* Disruption of gentle 'touch' reception causes lethargic animals

a, Violin plots showing the distribution of the backward and forward velocity. *P*-value derived from two-sided KS-test. **b,** i) Distribution of the crawling speed comparing wild-type hermaphodites and males with males and hermaphrodites from *mec-2* and *mec-4* mutant alleles. *P*-values above the edges connecting the color-coded nodes were derived from a two-sided Dunn test with Bonferroni adjustments for multiple comparison. Exact number of measurements and replicates displayed in Supplementary Table 3. Black circles indicate medians; vertical lines, 95% confidence intervals. ii) Estimation plots of bootstrapped Cliff's Delta (red points indicate median, red bars show 95% bias-corrected confidence intervals) for each comparison relative to the reference genotype. A dashed horizontal line at zero indicates no effect. **c,** Chronic ablation of TRNs using miniSOG and blue light. Blue violin indicates the wild-type animals under 4 min of continuous illumination of blue light. Orange violin indicates miniSOG expressed in TRNs under the *mec-4* promoter but kept in the dark before recording behavior under red light. Green violin indicates miniSOG expressing animals under continuous illumination with blue light. *P*-value derived from two-sided KS-test. **d,** Average crawling speed of animals expressing tetanus toxin light chain (UAS::TeTx) in TRNs using the *mec-4*p::GAL4 driver. Blue violin indicates UAS:TeTx effector alone; orange violin indicates combined expression of TRN:Gal4 driver and TeTx effector. *P*-value derived from two-sided KS-test. **e,** Individual locomotion trajectories for wild-type N2 animals and animals expressing ACR1 in touch receptor neurons, supplemented with ATR. **f,** Average crawling speed of animals expressing Anion channelrhodopsin 1 (ACR1) in TRNs under the indicated combinations of UAS::ACR1, *mec-4*p::GAL4, ATR ($\pm$) and illumination wavelength (hν = 519 nm or 630 nm). Conditions that permit light-activated ACR1 activity (ATR present + appropriate wavelength) show a pronounced decrease in crawling speed versus matched controls (ATR- or non-activating wavelength), demonstrating wavelength- and cofactor-dependent modulation of behavior. *P*-values above the brackets derived from a two-sided *t*-test.

Supplementary Figure 2

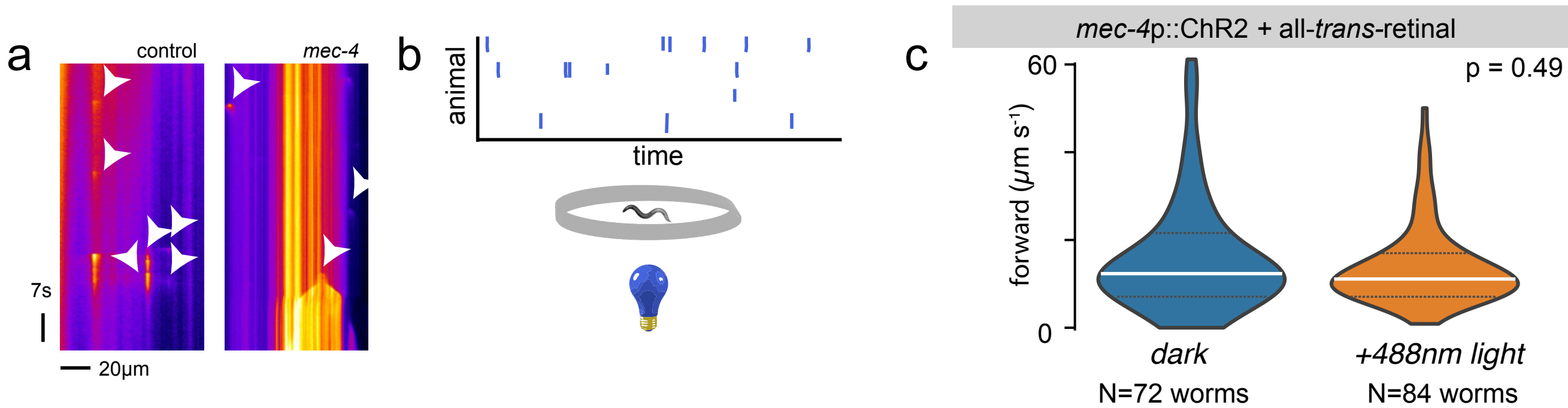

Supplementary Fig. 2. Random, ectopic activation of TRNs does not suppress *mec-4* induced lethargus

a, Spontaneous calcium fluctuations in PLM neurites in immobilized wild-type (left) and *mec-4* (right) mutant animals **b,** Schematics of the experiment, where a cohort of animals is illuminated with a Poisson-distributed stimulus for 48h. **c,** Average crawling speed of *mec-4* animals expressing Channel-rhodopsin2 in TRNs under the indicated stimulation protocol. Note, even though animals immediately respond to blue light, their spontaneous locomotion behavior is unchanged, indicating that occasional neuronal activity throughout larval development is not sufficient to suppress the *mec-4* induced lethargus. *P*-value derived from two-sided KS-test.

Supplementary Figure 3

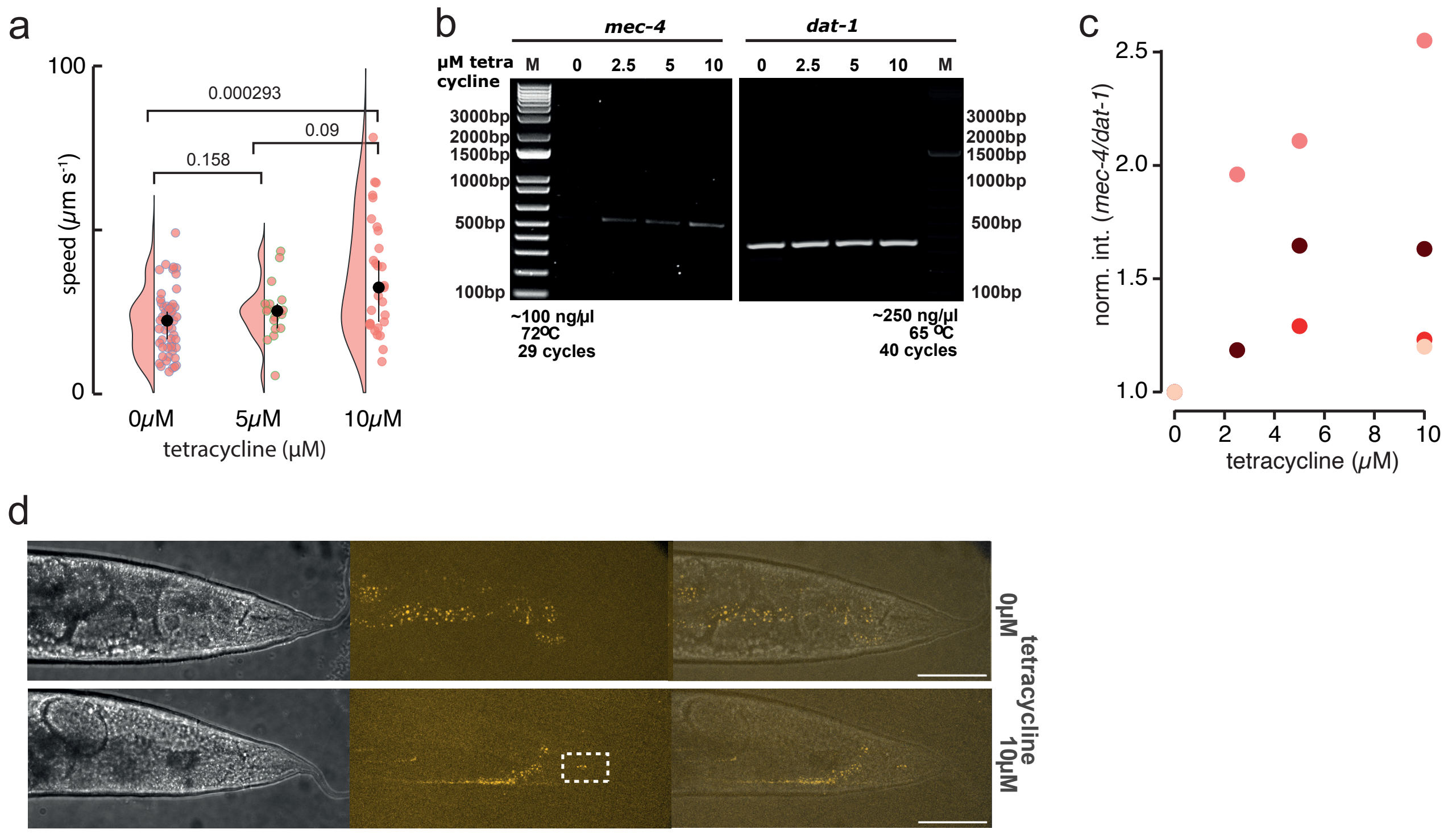

Supplementary Fig. 3. Temporal *mec-4* regulation through a tetracycline-dependent ribozyme **a,** Average crawling speed of wild-type animals exposed to varying concentrations of tetracycline. *P*-values above the bracket show the result of a two-tailed Kolmogoroff-Smirnoff test for a pairwise comparison of the indicated conditions. Black circles indicate the median, vertical lines the 95% confidence interval of the median. **b,** Representative gel image of tetracycline-dependent *mec-4* expression and *dat-1* as a tet-independent control. **c,** Quantification of *mec-4* gene expression. Independent experiments are represented as a different color. N = 4 biological replicates. Each data point was normalized to untreated control. **d,** Brightfield and fluorescent micrographs of an animal with and without tetracycline addition. The PLM cell body is indicated in the dotted box. Image is representative for N=10 animals that have been examined. Scale bar = 40 μm.

Supplementary Figure 4

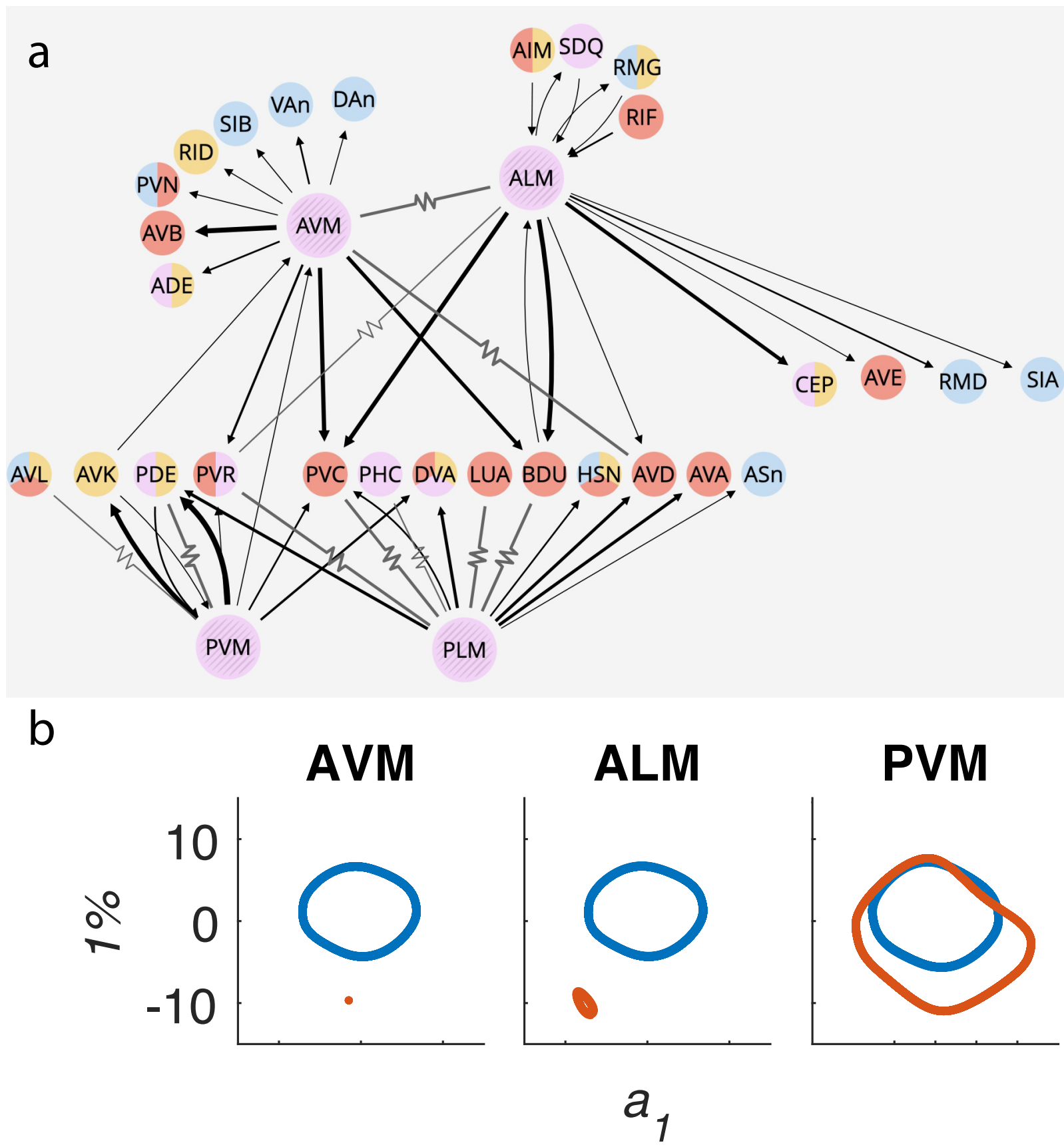

Supplementary Fig. 4. Simulation of the motor output in the *mec-4* mutant
a, Depiction of the TRN connectome including anterior and posterior touch receptors. Only posterior neurons are coupled to proprioceptive PDE and DVA neurons. Adapted from nemanode.org **b,** Representation of the combined motor neuron activity in the low dimensional subspace after driving forward locomotion through current injection into PLM (see also ref. (*1*)). We simulated TRN sensory capacity by injecting a baseline current of 1% of the driving current into AVM, ALM and PVM (blue limit cycle). Disruption of the basal current (e.g. simulating *mec-4* mutant condition) leads to a loss of motor neuron activity, visible as fixed points in the subspace of AVM and ALM, and the described increase in the limit cycle in the PVM subspace.

Supplementary Fig. 5

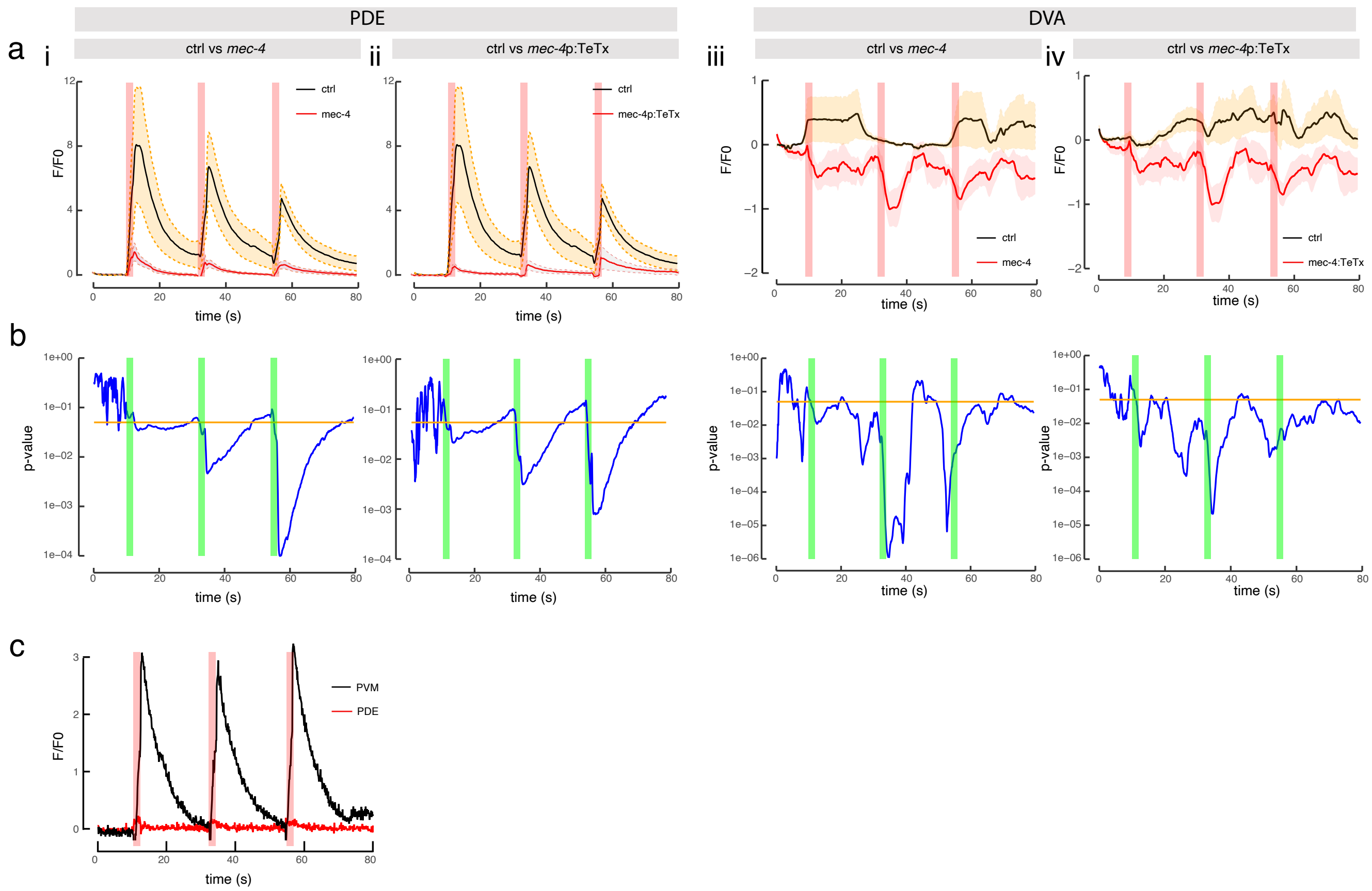

Supplementary Fig. 5. Statistical comparison of the calcium response after direct mechanical stimulations a, Plots showing the average fluorescence intensity of the calcium recordings between the indicated genotypes. Recordings of PDE in (i) ctrl vs *mec-4* and (ii) ctrl vs *mec-4p*:TeTx expressing animals. Recordings of DVA in (iii) ctrl vs *mec-4* and (iv) ctrl vs *mec-4p*:TeTx expressing animals. Solid line indicates mean, shaded area indicates standard deviation. **b,** Graphs showing a one-sided *p*-value (left axis) derived from a *t*-test statistics and the average calcium dynamics (right axis) vs time (bottom) for the indicated comparisons. The running, time-dependent *p*-value is indicated as blue line, the alpha-level of significance indicated as orange line. We consider the recorded time point different to the control when the blue line drops below the orange level of significance. **c,** Representative recording of PVM and PDE in a *mec-4*p::TeTx transgenic animal.

Supplementary Figure 6

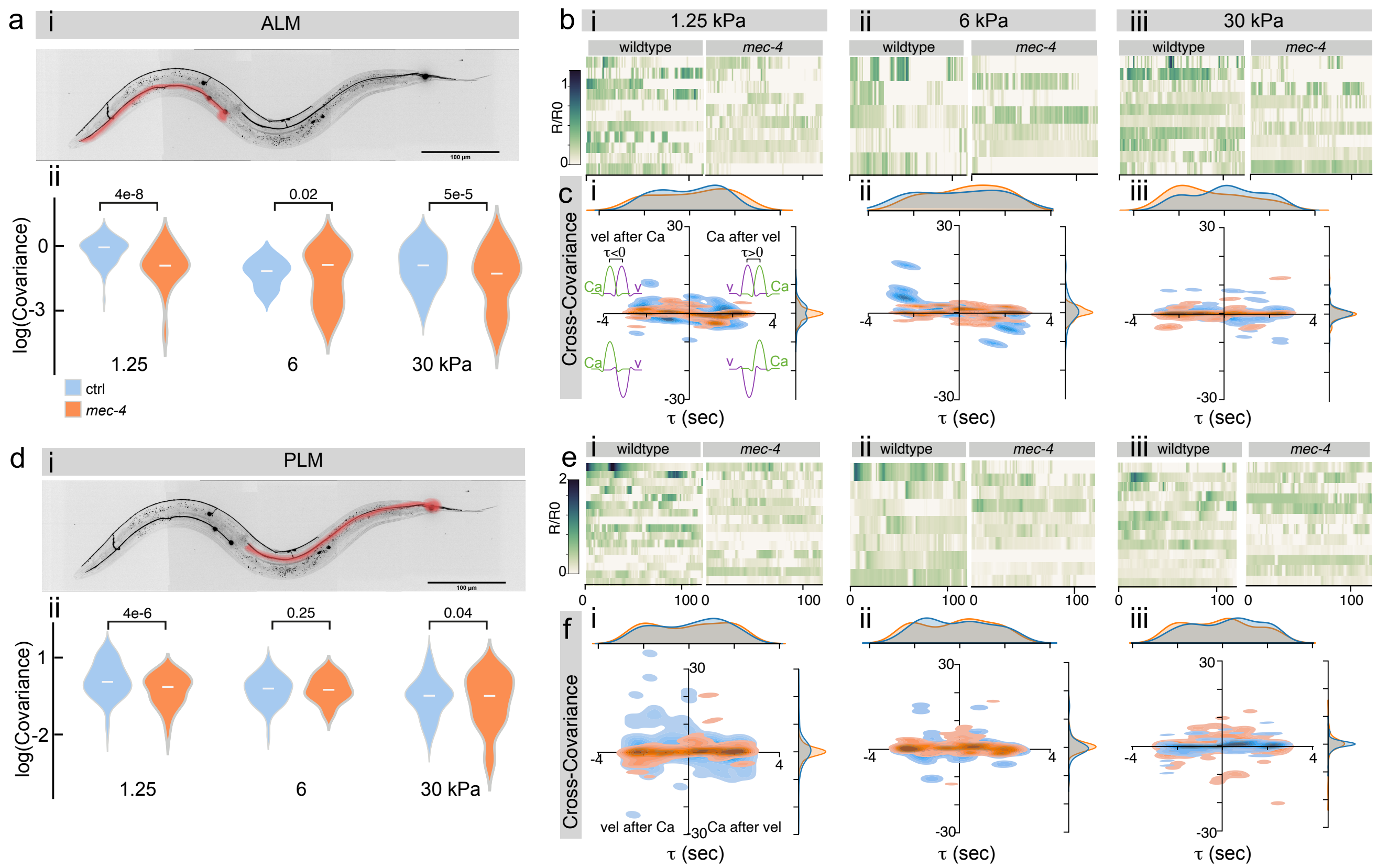

Supplementary Fig. 6. ALM and PLM calcium imaging in freely moving animals on different substrates
a, (i) Sketch of an animal with ALM highlighted in red. (ii) Covariance between forward velocity and calcium activity in ALM. *P*-values derived from a two-sided Wilcoxon rank-sum test. **b,** Kymographs of wild-type and *mec-4* mutant ALM activity during free locomotion on PAA gels with different stiffness. i) 1.25kPa; ii) 6kPa; iii) 30kPa. **c,** Cross-Covariance of ALM activity and forward locomotion velocity crawling on i) 1.25kPa; ii) 6kPa; iii) 30kPa. hydrogels. Inset inside the four quadrants of panel (i) indicates the temporal relation between the velocity and the calcium signals. **d,** i) Sketch of an animal with PLM highlighted in red. (ii) Covariance between forward velocity and calcium activity in PLM. *P*-values derived from a two-sided Wilcoxon rank-sum test. **e,** Kymographs of wild-type and *mec-4* PLM activity during free locomotion on PAA gels with different stiffness. i) 1.25kPa; ii) 6kPa; iii) 30kPa. **f,** Cross-Covariance of PLM activity and forward locomotion velocity crawling on i) 1.25kPa; ii) 6kPa; iii) 30kPa hydrogels.

Supplementary Figure 7

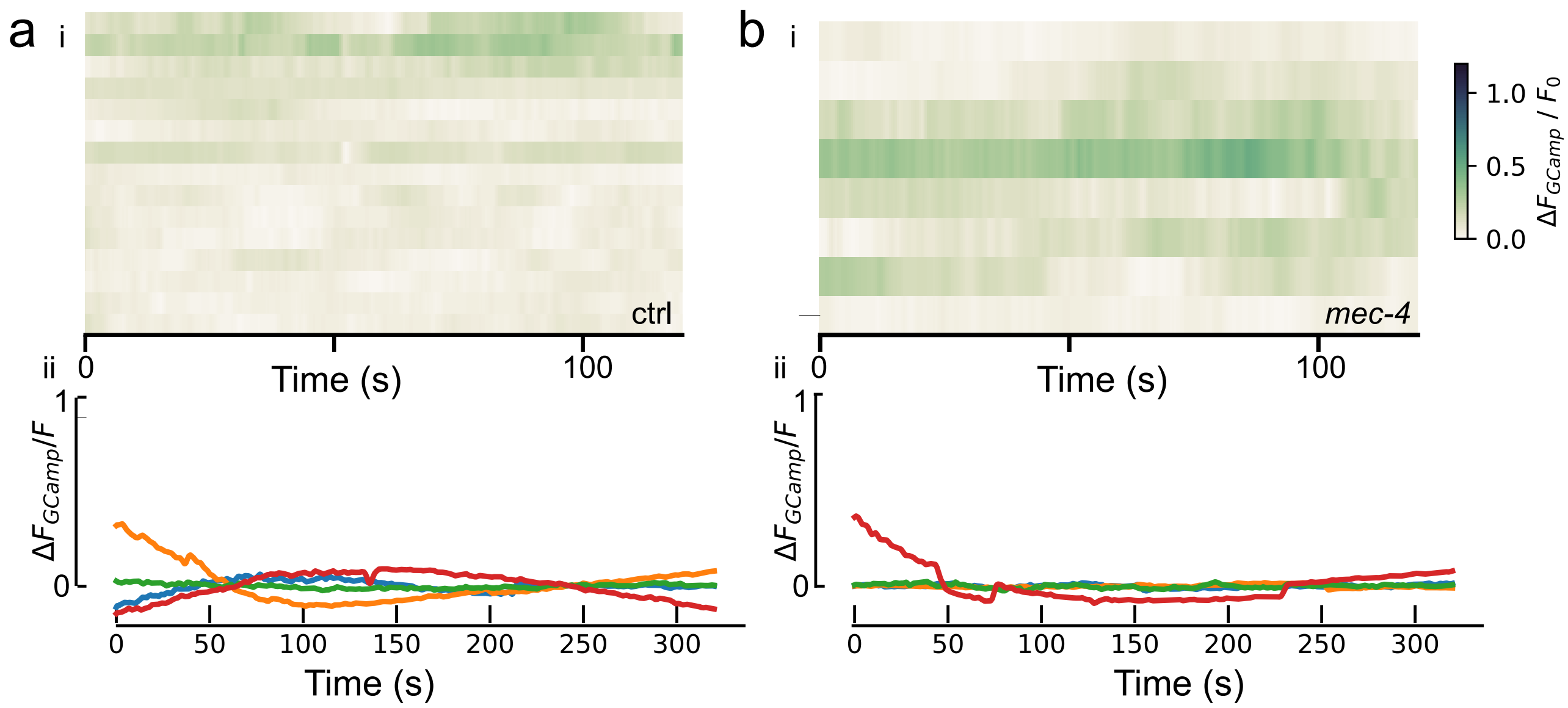

Supplementary Fig. 7. TRNs do not mobilise calcium in immobilized animals
a, b, Kymograph (i) and representative traces (ii) of the calcium activity measured in PLM cell body of immobilized (a) wild-type, and (b) *mec-4* mutant animals, as opposed to spontaneous, locally confined and non-propagating calcium transients observed in TRN neurites (Supplementary Fig. 2a.)

Supplementary Figure 8

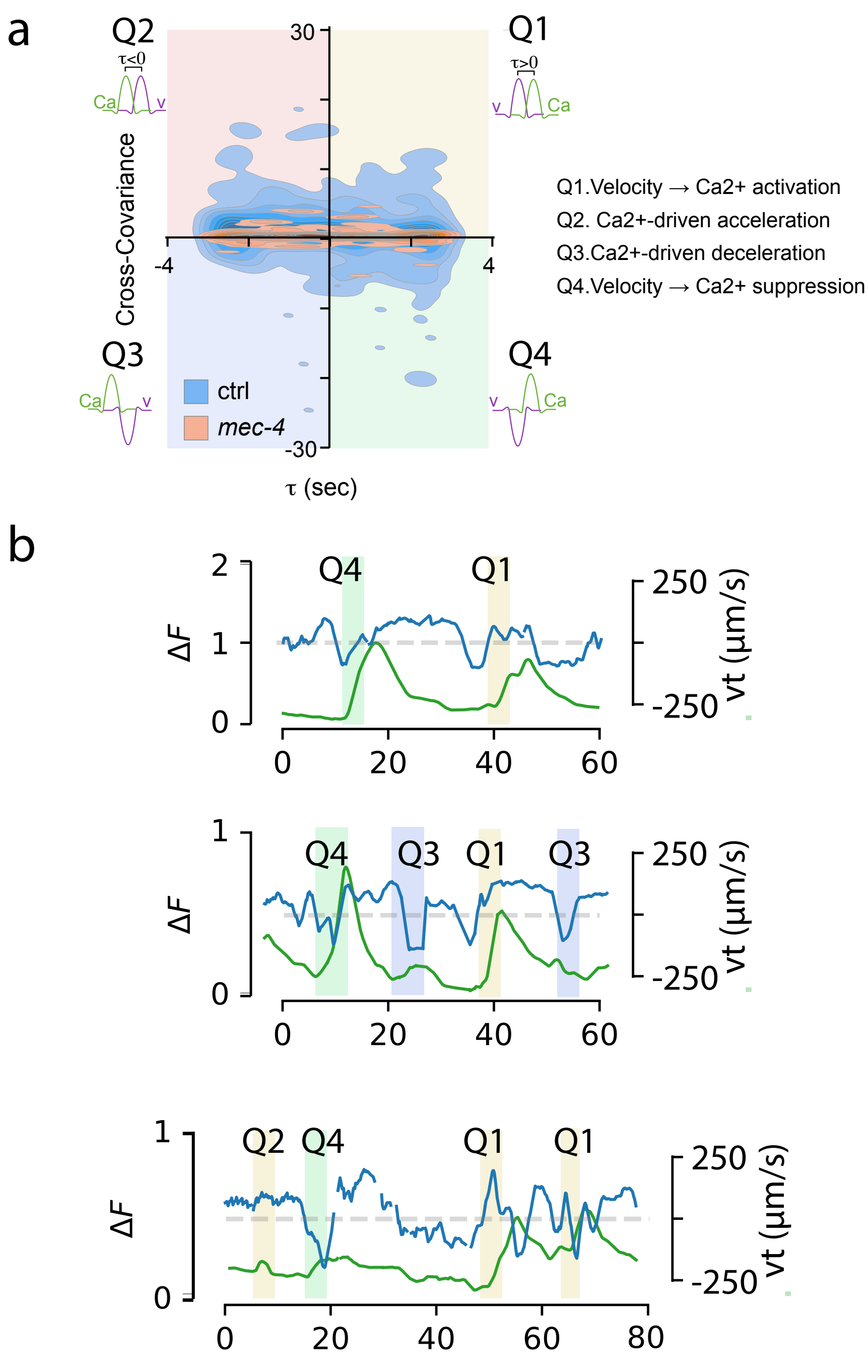

Supplementary Fig. 8. Individual events and how they contribute to the Calcium - velocity cross-covariance.
a, Detailed description and case examples for the cross-covariance computed between PVM:GCaMP intensity and animal crawling velocity. **b,** Highlighted events that map into the cross covariance.

Supplementary Figure 9

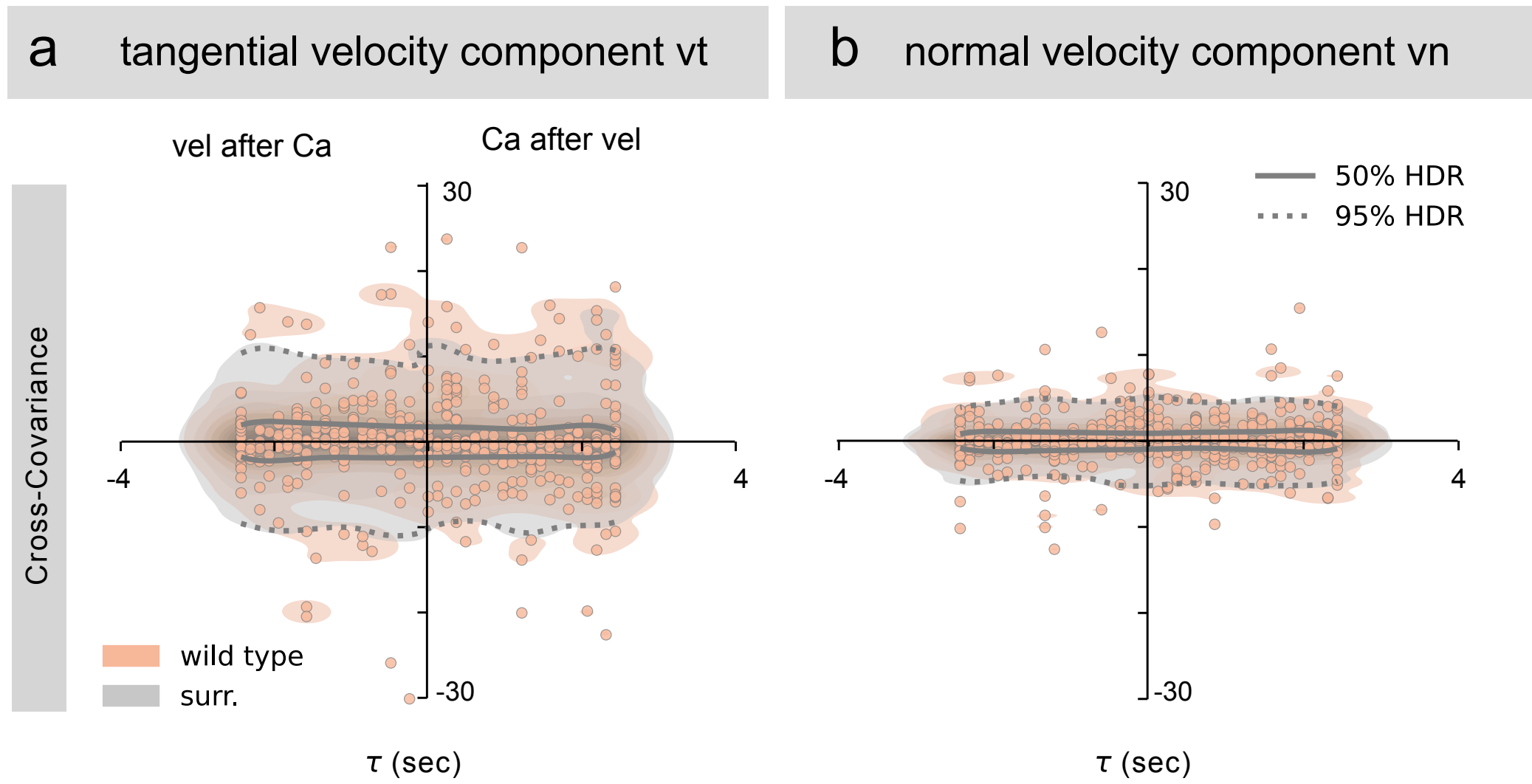

Supplementary Fig. 9. PVM activity co-varies with tangential (and not normal) components of velocity

a, b, Cross-covariance plots between PVM calcium dynamics and velocity components indicating the position of the cross covariance extrema for 5-seconds windows for wild-type worms and inter-subject surrogates for (a) tangential, v_t and (b) normal, v_n velocity components respectively. Surrogate analysis indicates that the local extrema in (a) are due to a real covariance between the time series, rather than due to internal, random fluctuations/properties of the individual time series.

Supplementary Figure 10

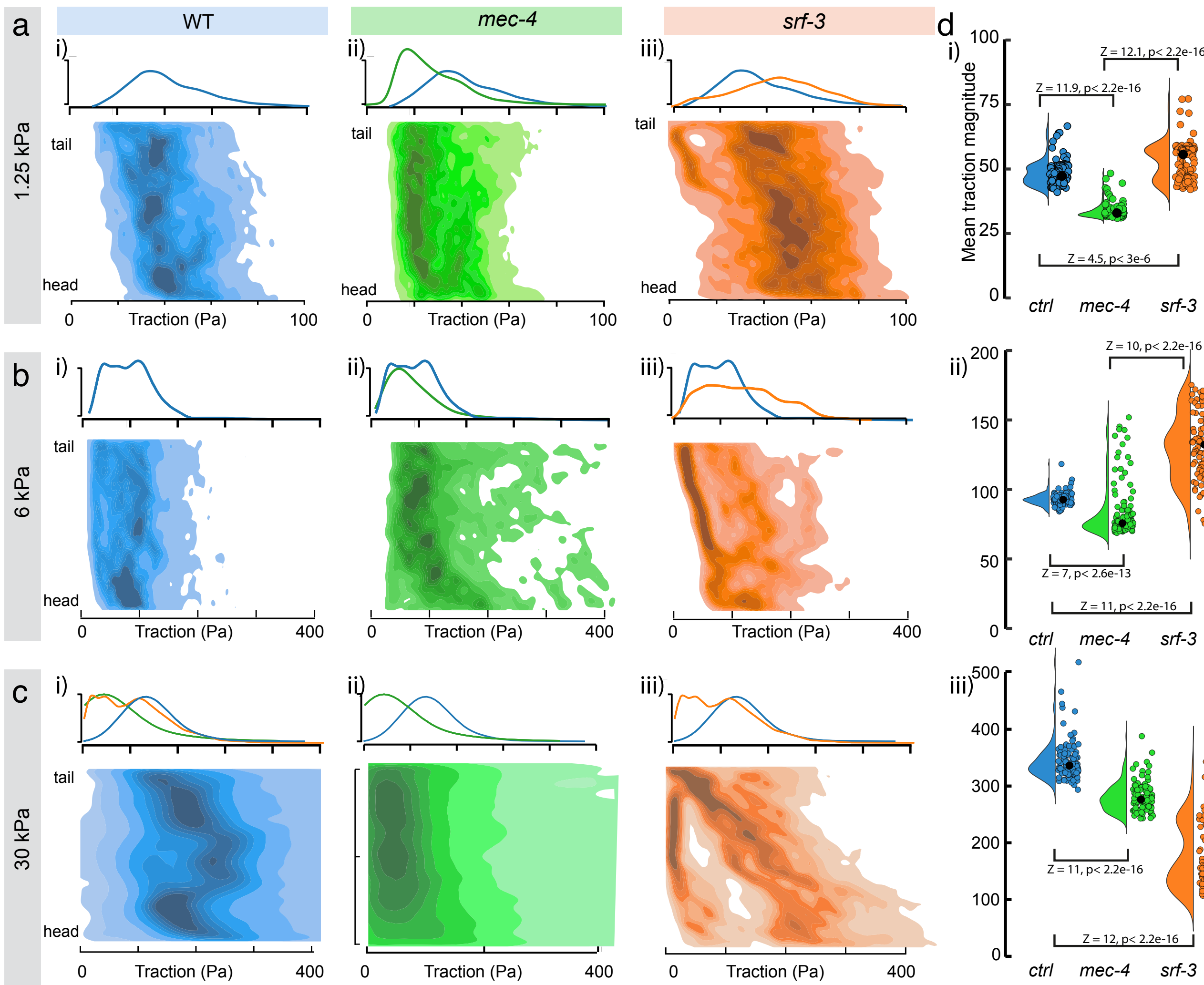

Supplementary Fig. 10. Traction forces of *mec-4* and *srf-3* mutants on different substrates
a-c, Distribution of traction forces along the body coordinate for (i) wild-type, (ii) *mec-4* and (iii) *srf-3* animals crawling on 1.25 kPa (a), 6 kPa (b) and 30 kPa (c) stiff polyacrylamide gels. Note, tractions and gel stiffness have the same units for stress and Young's modulus, respectively. Densities correspond to number of observations in a smoothed KDE. **d,** Statistical comparison of the tractions per strain and along body coordinate using a linear mixed-effects model. Each point indicates the average of each body coordinate for each strain. (i–iii) Analysis revealed a significant effect of body position (all $p < 2\times10^{-16}$) and a significant strain × body position interaction at all substrate stiffnesses (all $p < 2\times10^{-16}$). A main effect of strain was observed only at i) 1.25 kPa ($F_{2,23.4} = 10.3$, $p = 6\times10^{-3}$), but not at ii) 6 kPa ($F_{2,18.1} = 2.98$, $p = 0.076$) or iii) 30 kPa ($F_{2,18} = 0.24$, $p = 0.76$). Body position effects were: i) 1.25 kPa: $F_{1,2571} = 1416.1$; ii) 6 kPa: $F_{1,2076} = 1319.1$; iii) 30 kPa: $F_{1,2076} = 400.2$. Interaction effects were: i) 1.25 kPa: $F_{2,2571} = 179.9$; ii) 6 kPa: $F_{2,2076} = 279.8$; iii) 30 kPa: $F_{2,2076} = 218$, indicating that strain-dependent differences in traction are spatially localized along the body axis. *P*-values indicated in the figure derived from a two-sided Wilcoxon-Mann-Whitney test. Black circles indicate the median, vertical lines the 95% confidence interval of the median.

Supplementary Fig 11

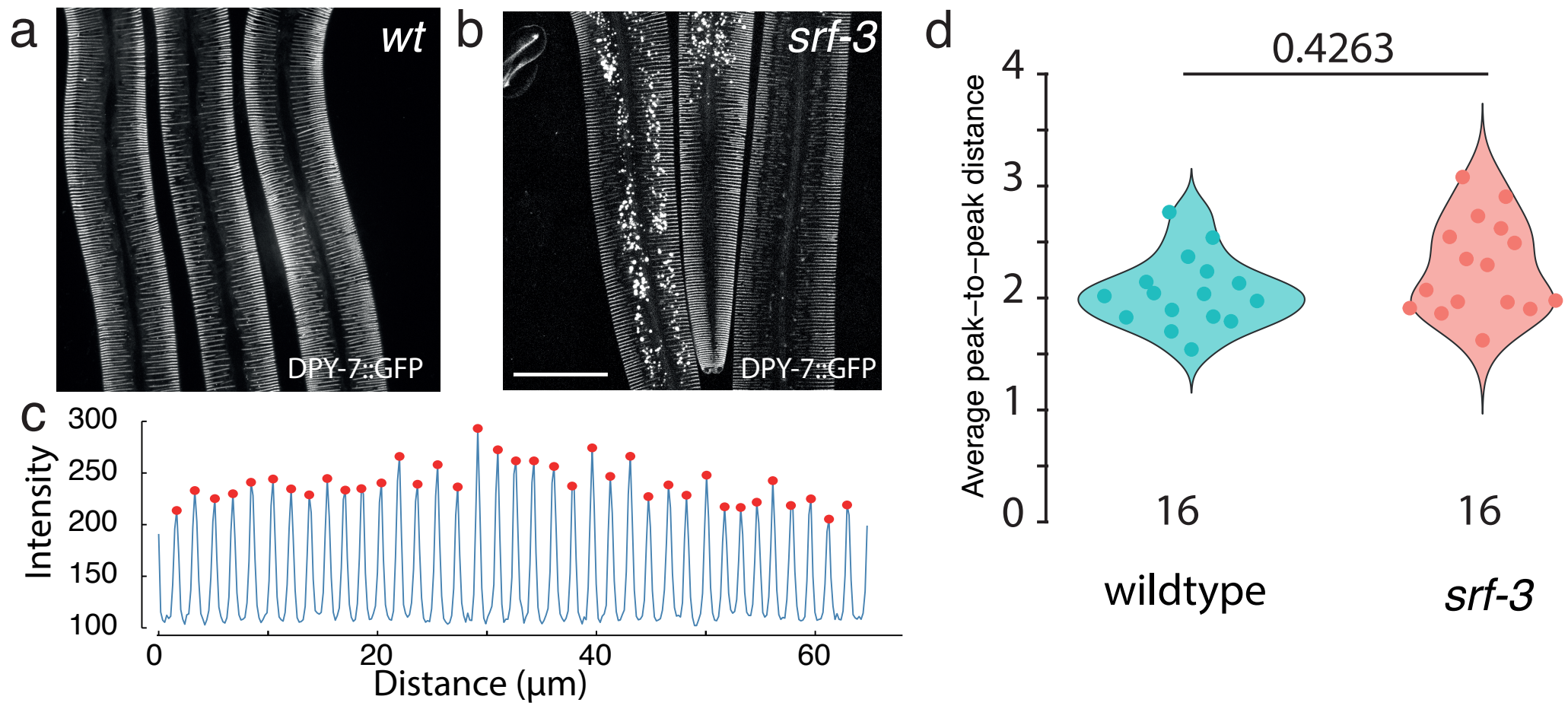

Supplementary Fig. 11. Characterization of *srf-3* mutant a, b Representative images of DPY-7::GFP expressing (a) wild-type and (b) *srf-3* mutant animals. **c,** Intensity profile of the DPY-7::GFP transgenic cuticle, labeling the furrows with the automatically detected peaks shown with a red dot. **d,** Comparison of the DPY-7::GFP ridge interval between wild-type and *srf-3* mutant animals. *P*-value derived from the two-sided KS-test, 16 = number of analyzed animals.

2 Supplementary Discussion

2.1 Mathematical modeling of neural network and behavioral proxy

2.1.1 Formulation

The model is based on an earlier work (*1–3*). The realistic *C. elegans* synaptic and gap junction connectome is used in conjunction with a single compartment neural model to simulate the dynamics of the network. Following (*1–3*), neurons were modeled with an isopotential single compartment membrane model, where the membrane potential of the i-th neuron V_i is given by the equation:

$$CV_i = -G^C(V_i - E_{cell}) - I_i^{Gap}\left(\vec{\mathbf{V}}\right) - I_i^{Syn}\left(\vec{\mathbf{V}}\right) + I_i^{Ext}, \tag{1}$$

where C is the whole-cell membrane capacitance, G_C is the membrane leak conductance and E_{cell} is the leakage potential. The external input current is given by I_i^{Ext}, while neural interactions via gap junctions and synapses are modeled by input currents $I_i^{Gap}\left(\vec{\mathbf{V}}\right)$ (gap) and $I_i^{Syn}\left(\vec{\mathbf{V}}\right)$ (synaptic), described by following equations:

$$I_i^{Gap}\left(\vec{\mathbf{V}}\right) = \sum_j G_{ij}^g \left(V_i - V_j\right) \tag{2}$$

$$I_i^{Syn}\left(\vec{\mathbf{V}}\right) = \sum_j G_{ij}^s s_j \left(V_i - E_j\right) \tag{3}$$

Gap junctions are taken as the ohmic resistances connecting each neuron, where G_{ij}^g is the total conductivity of the gap junctions between i and j. The synaptic current is modeled as a diffusive coupling and depends on the displacement from the reversal potentials E_j. G_{ij}^s is the maximum total conductivity of synapses to i from j, modulated by the synaptic activity variable s_i, whose temporal evolution is governed by:

$$\dot{s_i} = a_r \phi\left(V_i; \beta V_{th}\right)\left(1 - s_i\right) - a_d s_i, \tag{4}$$

where a_r and a_d correspond to synaptic activity rise and decay time, and ϕ is sigmoid function, given by:

$$\phi\left(V_i; \beta, V_{th}\right) = \frac{1}{1 + e^{-\beta(V_i - V_{th})}}. \tag{5}$$

The parameters are selected as in (*1*), unless started otherwise.

2.1.2 Network driver selection

We have tested several neuronal candidates receiving the constant current for drivers of the network, namely: AVB (as it delivers command signals to B-type motor neurons and it is known to drive forward locomotion, (*4*)), PLM (as it mediates the gentle touch response, triggers forward escape reflex and it was originally proposed as a network driver for this model (*1*)), AVF (as it was already reported as a persistent neuronal oscillator (*5*)). To find the feasible input current amplitudes that generate the oscillatory activity in the network Jacobian eigenspectrum was estimated using Matlab for various amplitude ranges. The values of input current amplitudes, for which at least one pair of the real part of eigenvalues was changing its sign, were selected to be used later as drivers. The oscillations in the network were the most stable when PLMR/L were used a network drivers. Therefore the following simulations were performed using PLMR/L neurons pair as a network driver.

2.1.3 Estimating the oscillatory modes representing forward locomotion

The constant input current applied to PLM neurons, $I_{PLM}^{Ext} = 2 \times 10^4$, results in the emergence of the oscillations in the whole neural network, as visualized in main text Fig 3c. Following the procedure originally proposed in (*1*), the singular value decomposition is then applied to B & D type motor neurons activity, which reveals an existence of the dominant oscillatory modes representing forward locomotion (*6*), indicated as a_1 & a_2 on Fig. 3d ii, and allows to find the weights corresponding to the contribution of each motor neurons in the locomotion modes.

2.1.4 Mechanosensitive current implementation

The model was originally used to computationally investigate the effect of injury or ablations on network dynamics. In this research, it was applied to investigate the effect of 'removing' the external input that represents the mechanosensitive current from the TRNs. An important assumption to mention is that the synapses were imposed half open at equilibrium membrane potentials (mean) (where $\phi(V_{eq}) = 0.5$, $\dot{V}_i = 0$, $\dot{s} = 0$), following (*1, 7*). This can be achieved by adjusting V_{th} in Eq. 5. In the first part of the simulation, 0-15 s, representing an intact worm receiving the mechanosensitive current, in addition to applying the PLM driving current, one of the TRNs (AVM, ALM or PVM) received a small constant input current with an amplitude varying between 0.5 and 5% of the driving current amplitude. The average membrane potential and thus the synaptic equilibrium, as well as the neuronal weights to each singular value, were established taking into account both the driving current and an additional 'mechanosensitive' input applied to one of the TRNs. In the second part of the simulation (20 - 30 s) this additional current was removed, which mimicked the loss of the mechanosensitive current. This resulted in an increase in the amplitude of the oscillations in the motor neuron membrane potential, leading to an increase of the body curvature, or a total extinction of these oscillations, resulting in the lack of motion, or in other words lethargus. This suggested that both increase in body curvature and lethargus, seemingly very different phenotypes, can have a common underlying origin in loss of mechanosensation in TRN. In the third part of the simulation (30-25 s), synaptic equilibrium values were adjusted to new values, by modifying V_{th} in Eq. 5 and taking into account driving current only (but no mechanosensitive currents), then the synapses were imposed half-open at new equilibrium values. This part represents adapted *mec* mutants.

2.1.5 Parameters

All the parameters are taken from the original paper (*1*), except where stated otherwise. Individual gap junction and synapse was assumed to have approximately the same conductance, roughly $g = 100pS$. Each cell has a smaller membrane conductance (taken as $10pS$) and a membrane capacitance of about $C_i = 1pF$ (*3*). Leakage potentials are all taken as $E_c = -35mV$ (*7*). Reversal potentials E_j are $0mV$ for excitatory synapses and $-45mV$ for inhibitory synapses. For the synaptic variable, we choose $a_r = 1$, $a_d = 5$ and define the width of the sigmoid by $\beta = 0.125mV^{-1}$. V_{th} is found by imposing that the synaptic activation $\phi = 1/2$ at equilibrium (*7*).

The simulation codes are available: `https://gitlab.icfo.net/NSMB/net-sim-worm`

2.2 Details of the worm locomotion model

Here we resort to the geometrically exact beam formulation in statics (*8*) and two dimensions, extended with active bending torques $m_{ac}(s, t)$ at midline coordinate s and time t, and a wet friction distribution, in agreement with resistive force theory (*9–11*). The latter is modeled as a distributed force on the beam

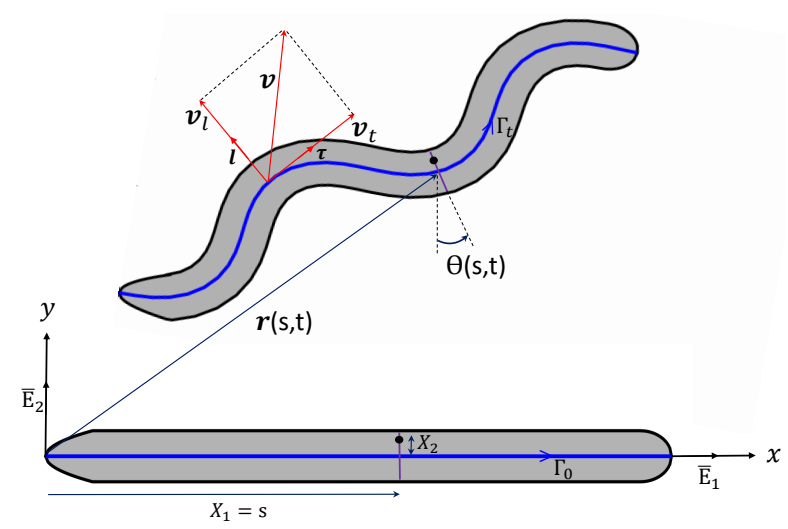

Supplementary Fig. 12. Scheme of slender body kinematics, indicating normal vector $\boldsymbol{v}_n$ and tangential component $\boldsymbol{v}_\tau(s,t)$, with $\boldsymbol{v} = \dot{\boldsymbol{r}}$ the velocity vector.

proportional to velocity $\dot{\boldsymbol{x}}$, and expressed as $-\boldsymbol{\eta}\dot{\boldsymbol{x}}$, with $\dot{\boldsymbol{x}} \equiv \frac{d}{dt}\boldsymbol{x}$ the velocity of the midline position $\boldsymbol{x}$, and $\boldsymbol{\eta}$ a frictional tensor given by (*12*)

$$\boldsymbol{\eta} = \eta_\tau \boldsymbol{\tau} \otimes \boldsymbol{\tau} + \eta_n \boldsymbol{n} \otimes \boldsymbol{n}. \tag{6}$$

Parameters η_τ and η_n correspond to tangential and normal frictional coefficients, and $\boldsymbol{\tau}$ and $\boldsymbol{n}$ the tangential and normal vectors at each point of the worm midline $s \in [0, L]$, with L the length of the worm (see Supplementary Fig. 12). Consequently, the beam equations read

$$\begin{aligned} \boldsymbol{f}' &= -\boldsymbol{\eta}\dot{\boldsymbol{x}} \\ m' + \boldsymbol{x}' \times \boldsymbol{f} &= m_{ac} \end{aligned} \tag{7}$$

where $()' = \frac{d}{ds}$, and vector $\boldsymbol{f} = \mathbf{R}\boldsymbol{F}$ denotes the axial forces at each cross-section, while $m(s,t)$ are the elastic bending moments. Rotation matrix $\mathbf{R}(s,t) = \begin{bmatrix} \cos\theta & \sin\theta \\ -\sin\theta & \cos\theta \end{bmatrix}$ defines the orientation of the cross-section, with $\theta(s,t)$ the angle of the rotation, as shown in Supplementary Fig. 12.

Note that in view of the friction tensor in (6), the external forces at the rhs of the first equation of (7) are equal to $-\boldsymbol{\eta}\dot{\boldsymbol{x}} = -\eta_t \boldsymbol{v}_\tau - \eta_n \boldsymbol{v}_n$, with $\boldsymbol{v}_\tau = (\dot{\boldsymbol{x}} \cdot \boldsymbol{\tau})\boldsymbol{\tau}$ and $\boldsymbol{v}_n = (\dot{\boldsymbol{x}} \cdot \boldsymbol{n})\boldsymbol{n}$ the tangential and normal components of the velocity vector $\boldsymbol{v} \equiv \dot{\boldsymbol{x}}$, respectively.

We assume an active elastic body of the worm, that is, the unrotated axial forces $\boldsymbol{F}$ and moment m are assumed to obey linear elastic constitutive laws, conjugate to strain measures $\mathbf{R}^T\boldsymbol{x}' - \mathbf{E}_1$ and θ',

$$\begin{aligned} \boldsymbol{F} &= \begin{bmatrix} EA & 0 \\ 0 & GA \end{bmatrix} (\mathbf{R}^T\boldsymbol{x}' - \boldsymbol{E}_1) \\ m &= EI\theta' \end{aligned} \tag{8}$$

Constants E, G, A, I are material and geometrical parameters corresponding to Young modulus, shear modulus, cross-section area and moment of inertia of the worm (Supplementary Fig. 12).

The direct problem of the worm consists of, given a time-history of spatial distributions of viscous parameters $\eta_t\tau(s,t)$ and $\eta_n(s,t)$, and a torque distribution $m_{ac}(s,t)$, finding the sequence of positions $\boldsymbol{x}(s,t)$ and angles $\theta(s,t)$, for an initial configuration $\boldsymbol{x}_0(s) = \boldsymbol{x}(s,0)$ and $\theta_0(s) = \theta(s,0)$.

We solve this direct problem resorting to a Finite Element (FE) discretisation of unknowns $\boldsymbol{x}$ and θ from a set of nodal values $\boldsymbol{y}_i = \{\boldsymbol{x}_i, \theta_i\}$, $i = 1, \ldots, N$. The FE method allows us to write the partial differential equations (PDEs) in (7), together with the constitutive law in (8), as a set of ordinary differential equations (ODEs), which may be written in a compact manner as,

$$\dot{\boldsymbol{y}} = \boldsymbol{f}(\boldsymbol{y}, \boldsymbol{u}) \tag{9}$$

where $\boldsymbol{y} = \{\boldsymbol{y}_1, \ldots, \boldsymbol{y}_N\}$ denotes the set of nodal general coordinates, and vector $\boldsymbol{u} = \{\boldsymbol{u}_1, \ldots, \boldsymbol{u}_N\}$ a set of nodal triplets $\boldsymbol{u}_i = \{\eta_\tau, \eta_n, m_{ac}\}_i$ containing the values of friction and torques at node i. We note that for writing the static beam equations in the form (9), we include a small wet friction contribution also to the rotations, that is, we add a term $-\eta_m\dot{\theta}$ to the rhs of the second equation in (7).

2.3 Inverse analysis through Optimal Control Theory

We consider the following inverse problem: given a time-history of worm kinematics (i.e., nodal positions and rotations $\boldsymbol{y}_{exp}(t)$), deduce the set of torques and frictional parameters $\boldsymbol{u}_i$ that best agree with the measured values $\boldsymbol{y}_i$ while satisfying beam ODEs in (9). Since the rotations θ_i cannot be experimentally measured, we assume that each θ_i corresponds to the orientation of the tangent of the midline, that is, we assume no shear deformations.

While direct problems are in general well-posed and have a unique solution, inverse problems may have multiple solutions and be very sensitive to initial data. For this reason, we adapt the formulation of optimal control problems (*13, 14*) to i) compute the optimal deformations for the locomotion of the worm (a new set of $\boldsymbol{y}_i$ that approximates $\boldsymbol{y}_{exp}$), and ii) deduce muscle activity from experimental measurement (best matching values of $\boldsymbol{u}_i$).

This inverse problem is formulated as the solution of the following optimization problem (*15, 16*):

$$\begin{aligned} &\min_{\boldsymbol{y},\mathbf{u}} \mathcal{J}(\boldsymbol{y}, \mathbf{u}) \\ &s.t.\ \dot{\boldsymbol{y}} = \boldsymbol{f}(\boldsymbol{y}, \mathbf{u}) \\ &\quad \boldsymbol{y}(0) = \boldsymbol{y}_0 \end{aligned} \tag{10}$$

with $\boldsymbol{y}_0$ the initial nodal positions and angles of the midline, and $\mathcal{J}(\boldsymbol{y}, \mathbf{u})$ the functional

$$\mathcal{J}(\boldsymbol{y}, \boldsymbol{u}) = \frac{1}{2}\int_0^T \left(||\boldsymbol{y}(t) - \boldsymbol{y}_{exp}||^2 + \alpha||\boldsymbol{u}||^2\right) dt, \tag{11}$$

which aims to minimize the distance from the calculated position to $\boldsymbol{y}_{exp}$, while also minimizing the cost $\boldsymbol{u}$ through the weight parameter α. The second (regularization) term also aims to furnish unique solution to the optimal control problem (OCP) in (10). This OCP can be solved by adding the adjoint variable $\boldsymbol{\lambda}$, which also reveals a symmetric structure and form,

$$\begin{aligned} &\dot{\boldsymbol{y}} = \nabla_\lambda \mathcal{H} \\ &\dot{\boldsymbol{\lambda}} = -\nabla_y \mathcal{H} \\ &\mathbf{0} = \nabla_u \mathcal{H} \\ &\quad \boldsymbol{y}(0) = \boldsymbol{y}_0, \boldsymbol{\lambda}(T) = 0 \end{aligned} \tag{12}$$

with $\mathcal{H}(\boldsymbol{y}, \boldsymbol{\lambda}, \boldsymbol{u}) = \frac{1}{2}\left(||\boldsymbol{y}(t) - \boldsymbol{y}_d||^2 + \alpha||\boldsymbol{u}||^2\right) + \boldsymbol{\lambda}^T(\boldsymbol{f} - \dot{\boldsymbol{y}})$ the Hamiltonian of the OCP. The equations above form a two-point Hamiltonian boundary value problem that we will solve resorting to a Forward-Backward Sweep Method (*13, 17, 18*).

In our optimization problem, the control variable $\mathbf{u}$ includes the active moments m_{ac} in Eq. (7), and the unknown friction coefficients η_t and η_n. We note that the three quantities depend on space and time, and are defined at each node of the FE discretisation, except m_{ac} which is equal to 0 at the end nodes. The resulting inferred values in $\mathbf{u}$, and optimal positions $\mathbf{y}$ that best match the experimental coordinates

$\boldsymbol{y}_{exp}$ allow us to compute the total dissipated and bending energy of the worm along the time span $[0, T]$ as,

$$E_d = \int_0^T \int_0^L \boldsymbol{\eta}\dot{\boldsymbol{x}} \cdot \dot{\boldsymbol{x}} dl\, dt = \int_0^T \int_0^L (\eta_n v_n^2 + \eta_t v_t^2) dl\, dt \tag{13}$$

$$E_b = EI \int_0^T \int_0^L (\theta')^2 dl\, dt \tag{14}$$

where $v_t = ||\boldsymbol{v}_t||$ and $v_n = ||\boldsymbol{v}_n||$ are the norms of the normal and tangential velocity components. We have taken the ratio E_d/E_b as a measure of lost energy during locomotion, which as shown in main text Fig. 8l is higher for the *mec-4* mutant.

Conversion of model output to traction stresses

The mechanical model is implemented in non-dimensional form. The governing equations are normalized by the bending stiffness k and multiplied by a reference length unit of $1\,\mu$m, which arises from the first-order spatial derivative in the formulation. Consequently, the code operates with $k = 1$, and dimensional quantities must be rescaled after simulation.

To recover physical units, model outputs are multiplied by the bending stiffness k and divided by the reference length $L = 1 \times 10^{-6}\,\mathrm{m}\,\mu\mathrm{m}^{-1}$.

Estimates from the literature suggest a nematode body bending stiffness of approximately $k \sim 10^{-16}\,\mathrm{N\,m}^2$ (*19*). For a discretized body segment of length $ds = 20\,\mu$m, this corresponds to an effective bending stiffness per element

$$k_s = \frac{k}{ds}, \tag{15}$$

which is the material parameter used in the numerical implementation. Substituting $k = 10^{-16}\,\mathrm{N\,m}^2$ and $ds = 20\,\mu$m yields

$$k_s = 0.5 \times 10^{-11}\,\mathrm{N\,m}. \tag{16}$$

The model output f corresponds to the net nodal force (N) rather than a traction (Pa). To convert forces into stresses, the force is distributed over the contact area associated with each node. Assuming a worm width of $20\,\mu$m and an element length of $20\,\mu$m, the corresponding area is

$$A = 20\,\mu\mathrm{m} \times 20\,\mu\mathrm{m} = 4 \times 10^{-10}\,\mathrm{m}^2. \tag{17}$$

The traction stress t is obtained from the simulated force f through

$$t = Cf, \tag{18}$$

where the conversion factor is

$$C = \frac{k_s}{LA}. \tag{19}$$

Using $k_s = 0.5 \times 10^{-11}\,\mathrm{N\,m}$, $L = 10^{-6}\,\mathrm{m}\,\mu\mathrm{m}^{-1}$, and $A = 4 \times 10^{-10}\,\mathrm{m}^2$ yields

$$C = 1.25 \times 10^4. \tag{20}$$

Thus, the traction stress in Pascals is obtained by multiplying the simulated nodal force by 1.25×10^4.

2.4 Notes on the time-series analysis of calcium activity and tangential locomotion

Since the seminal contribution of the Chalfie lab in determining the role of individual touch receptor neurons to the mechano-escape reflex (*4, 20*), the consensus in the field is that PVM does not contribute to the behavioral pattern generation, despite being mechanosensitive in itself (*21, 22*). Behavioral experiments carried out on animals with individually laser-ablated neurons have revealed that anterior neurons ALML/R and AVM are mediating a reversal escape response when animals are stimulated on the anterior body segments, PLML/R have been shown to be crucial for acceleration when animals are stimulated on the posterior body part. In contrast, killing PVM has little consequences on touch-evoked behavior, despite PVM being activated by body touch (*21, 22*). In particular, from the work presented in reference (*4*) paper we learned: *"Ablation of PVM has no detectable effect upon touch sensitivity. Killing all the microtubule cells, or all except PVM, results in animals that are touch insensitive over their entire length, except at the very front of the head."* Thus, functional significance for PVM in the touch reflex has yet to be demonstrated, and remains unknown (*23, 24*). In addition, a recent investigation aiming to decipher the role of individual TRNs on behavior suggested that PVM has a modulatory, rather than a driving role (*25*).

In our experiments which address the role of TRNs in locomotion behavior, we observed that the calcium dynamics showed a complex and context dependent behavior.

The plots in Figure 5, 6 and Supplementary Figure 6, 8 and 9, we display the distribution of cross-covariance values between calcium activity in the sensory neuron and locomotion velocity across lags (τ). Cross-covariance was computed for each recording trace, and the distributions for control and *mec-4* animals are shown as density maps (center) with their respective marginal distributions projected onto the top and right axes. Importantly, cross-covariance preserves the absolute magnitude of joint fluctuations and is therefore sensitive to changes in signal amplitude, making it well suited for detecting causal structure and asymmetric coupling. In contrast, cross-correlation normalizes the signals and highlights only relative similarity, which can obscure amplitude-dependent effects. Because our goal was to capture how variations in locomotor dynamics scale with, and potentially activate, changes in TRN activity, cross-covariance provided a more informative measure than correlation.

Lags are defined such that positive τ values indicate velocity leading calcium, and correspond to sensory encoding, where changes in velocity precede and are followed by changes in neuronal calcium. In contrast, negative τ values indicate calcium leading velocity and the left quadrants reflect motor command generation, where neuronal calcium elevations precede/cause behavioral changes in crawling velocity.

The density of the cross-covariance values near τ = 0 in PVM indicates a tight temporal coupling between sensory neuron activity and ongoing locomotion. Whereas control animals show a more pronounced and consistent covariance peak at negative and positive lag, *mec-4* mutants generally exhibit a broader and less coherent distribution, suggesting reduced or more variable coupling when mechanotransduction is impaired.

Whereas the function of TRNs is well studied and they mediate escape reflex to mechanical stimuli, the function of the PVM neuron is currently not known. We investigated the hypothesis that it encodes aspects of the animal's movement (responding to velocity). The covariance structure therefore reflects a combination of sensory encoding (velocity $\rightarrow$ Ca; $\tau >$0) and motor command generation (Ca $\rightarrow$ velocity change; $\tau < 0$).

In control animals, the sharp covariance structure suggests:

1. Reliable sensory encoding: changes in velocity are consistently followed by changes in calcium at

short positive lags.

2. Effective motor output: increases in Ca often precede behavioral change in locomotion at short negative lags.

In *mec-4* mutants, the flat covariance patterns indicate an impaired mechanosensory drive: the neuron's calcium signal tracks velocity less reliably. It also suggests a weaker motor influence: Ca events do not translate into consistent reversal initiation or velocity changes.

The overall reduction in covariance amplitude and temporal precision in *mec-4* animals is consistent with a loss of mechanosensory input through the MEC-4 channel, affecting both how movement is encoded and how sensory activation is converted into behavioral output.

As displayed in the Supplementary Figure 8, the individual quadrants can be explained by the following:

1. Q1: The positive lag of the positive cross-covariance indicates that movement triggers a change in calcium activity. This is consistent with the hypothesis outlined here that PVM is activated by velocity-dependent substrate sensing (e.g. traction-dependent activity).

2. Q2: The negative lag of the positive covariance indicates that calcium activity triggers forward movement. This is consistent with the idea that PVM causes accelerations similar to other posterior touch receptor neurons such as PLM.

3. Q3: The negative lag of the negative covariance indicates that the increase in calcium activity in PVM triggers decrease in velocity. This is neither inconsistent with the idea of the standard touch response nor with our hypothesis, and indeed this is the quadrant with the least observations in our data.

4. Q4: The positive lag of the negative covariance indicates that the decrease in locomotion velocity triggers a PVM calcium transient. This is consistent with the hypothesis that PVM is activated by velocity-dependent substrate sensing (e.g. traction-dependent activity). The notion that PVM is activated by forward and backward locomotion points towards a direction-invariant traction sensing mechanism. Further studies need to be conducted to investigate if traction is larger during forward or backward crawling.

We have highlighted how these quadrants map onto the raw calcium and tangential velocity traces in the Supplementary Figure 8.

Because of the high covariance in quadrant 1, it gives first direct hints that PVM has a role on substrate and velocity sensation as studied in detail in this paper. The high covariance in Q2 also suggests that PVM may drive acceleration and reversals. Together, we put the idea forward that PVM has a dual context dependent role as a substrate mechanosensor to adjust proprioception and motor output.

2.5 Additional discussion about parameter estimation from traction force microscopy

How much drag force does the animal experience moving through the viscous medium? Using resistive force theory for a slender cylinder of length $L = 1$mm and radius $a \approx 40\ \mu$m, moving laterally at velocity $v = 50\mu$m/s in a fluid of viscosity $\eta = 0.19$Pa·s, we obtain a transverse drag per unit length of $f_\perp \approx \frac{4\pi\eta v}{\ln(L/a)+1/2} \approx 1.2 \times 10^{-4}\mathrm{N/m}$.

Over a TRN-relevant length scale of $\Delta x = 50\mu$m this corresponds to a local load of only $F_{viscous} = f_{\perp}\Delta x \approx 6$nN. This value is at least an order of magnitude below the ≈100-200nN indentation forces reported to reliably activate TRNs with a glass probe (*39*) supporting the conclusion that viscous load is insufficient to robustly engage TRNs.

However, during crawling on agar, frictional forces can be estimated as $F_{friction} = \mu N$, where μ is the effective coefficient of friction and N is the normal load. Traction force studies report effective frictional forces in the range of $10 - 100\mu$N per animal (***?***). On the scale of a 50μm TRN segment, this corresponds to hundreds of nN of local load - well above the activation threshold for MEC-4 - dependent mechanotransduction.

We directly estimated the frictional forces using our mathematical model. Our calculated tangential friction coefficient (η_t) of $\approx 3 \cdot 10^6$ Pa·s/m (Fig. 8h) indicates strong mechanical coupling between the worm and the substrate suggesting that small body movements generate substantial mechanical stresses on the cuticle that could be sensed by the underlying MEC-4 ion channels. For local slip velocities of 10–100 μm s^{-1}, this coefficient corresponds to tangential stresses of approximately 30–300 Pa using the relation

$$t = v_t \cdot \eta_t$$

, similar to the measured values (Fig. 8e). Assuming an effective local contact area of 10^{-9} m^2, interaction forces of roughly ≈300 nN, the same order of magnitude of contact forces known to activate TRNs (*39*). This contact area is an estimate from our traction force visualization (Main Text figure 8e), showing that the animal produces uneven tractions with patches of several 100 μm along the whole contour length. This assumption is reasonable, because an undulating worm does not necessarily contact the substrate uniformly along its entire body, and the effective contact width depends on body shape during bending, indentation, fluid film thickness, and substrate compliance.

Together, both the experimental recordings and the force estimates indicate that viscous drag alone is not sufficient to strongly activate TRNs. Instead, TRNs are robustly engaged by shear stresses that arise from frictional coupling to a soft, viscoelastic substrate during crawling.

Although viscous drag forces estimated from resistive force theory are only a few nanoNewtons over the length of a TRN process, comparison with indentation thresholds measured using a glass probe should be interpreted with caution because these represent fundamentally different loading modes. Glass-probe stimulation generates localized normal compression of the cuticle, whereas locomotion in a viscous fluid produces distributed tangential hydrodynamic stresses. During crawling on a compliant substrate, tangential body motion is mechanically coupled to the elastic substrate, producing substrate deformation and reaction forces that may generate substantially larger local strains within the cuticle. Thus, the observed dependence of neuronal activity on tangential velocity on soft substrates is consistent with TRN activation being determined by substrate-mediated tissue deformation rather than tangential velocity or hydrodynamic drag alone.

3 Supplementary Tables

Supplementary Table 1

plasmid name	promoter	ORF	3'UTR
pNM3	*mec-4*	cGal4	*let-858*
pNS50	*mec-4*	mScarlet1-10	*unc-10*
pNMSB184	*flp-8*	N-CRE	*tbb-2*
pNMSB185	*twk-49*	C-CRE	*tbb-2*
pNMSB186	*mec-4*	mScarlet::SL2::TeTx	*let-858*
pNS121	*mec-18*	*mec-4*(cDNA)::SL2::mCherry	*unc-10*

Table 1. Plasmids used in this study.

Supplementary Table 2

Table 2. Strains used in this study

Strains	Genotype	Source	Used in Fig.
N2 Bristol	wild-type	CGC	Fig. 1, 8, 9 & Suppl. Fig. 1, 10, 11
AQ2334	*lite-1(ce314)*; ljIs123[*mec-4*p::ChR2::YFP+*unc-122*p::RFP]	gift from Bill Schaefer (*26*)	used as a parental strain for MSB990
CB75	*mec-2(e75)* X	(*27*)	Fig. 1, Suppl. Fig. 1
CB1611	*mec-4(e1611)* X	(*28*)	Fig. 1
CZ23281	*juEx7105*[*mec-4*p::PH::miniSOG(Q103L) + *mec-4*p::mCherry + *ttx-3*::RFP]	(*29*)	Suppl. Fig. 1
GN692	*ljSi1*[*mec-7*p::GCaMP6s::SL2::tagRFP] II; *lite-1(ce314)X*	(*22*)	Fig. 5, 6; Suppl. Fig. 6-9
GN693	*ljSi1*[*mec-7*p::GCaMP6s::SL2::tagRFP] II; *mec-4*(u253), *lite-1(ce314)* X	(*22*)	Fig. 5,6; Suppl. Fig. 6-9
KG1180	*lite-1(ce314)*	(*30*)	background strain in several cases
MSB26	*mec-12(e1607)* III	this study	Fig. 1
MSB273	*syIs423*[15xUAS::Δ*pes-10*p::GCaMP6s::SL2::mKate2 ::*let-858* 3'UTR + *myo-2*p::NLS::mCherry] V; *mirIs19*[*nlp-12*p::GAL-4; *unc-122*p::mCherry]	(*31*)	Fig. 4
MSB586	*mirEx241*[*mec-4*p::cGAL4; *myo-2*p:mCherry]; *syIs421*[15xUAS::Δ*pes-10*::tetx::*let-858* 3'UTR + *myo-2*p::NLS-GFP + pBlueScript]	this study	used for generating MSB683
MSB636	*otIs669* V; *otIs672*; *mec-4(u253)*, *lite-1(ce314)* X	this work, obtained by OH1555 x GN693	Fig. 4, Suppl. Fig. 5
MSB655	*mec-2*(*mir42*[*mec-2*::GFP])	(*32*)	Fig. 1
MSB661	*otIs669* V; *otIs672*; *lite-1(ce314)* X	this work, obtained by OH15000 x GN693	Fig. 4, Suppl. Fig. 5
MSB683	*mirIs66*[*mec-4*p::cGAL4; *myo-2*p:mCherry]; *syIs421*[15xUAS::Δ*pes-10*p::tetx::*let-858* 3'UTR + *myo-2*p::NLS::GFP + pBlueScript]	this work, UV insertion MSB586	Suppl. Fig. 1
MSB797	*dpy-10(0)II*; *mec-4*(*mir50*[mScarlet(11)::*mec-4*])X	this work	parent strain to build MSB943
MSB927	*mirEX238*[*mec-4*p::cGAL4+*myo-2*p:mCherry]	this work	parent strain to obtain MSB1244
MSB931	*mec-4(mir50*[mScarlet(11)::*mec-4*]), *lite-1(ce314)*X	this work	parent strain to build MSB943
MSB932	*mec-4(mir50*[mScarlet(11)::*mec-4*]), *lite-1(ce314)*X; mirEx384(*mec-4*p::mScarlet1-10; *unc-122*p::GFP)	this work	parent strain to build MSB943

MSB943	*mec-4(mir50*[mScarlet(11)::*mec-4*]), *lite-1(ce314)*X; *mirIs93*(mec-4p::mScarlet1-10; unc-122p::GFP)	this work	used as background strain for several edits
MSB952	*mirIs97* [15XUAS::ACR1::*let-858* 3'UTR **oxTi677* [*eft-3*p::tdTomato::H2B::*unc-54* 3'UTR + *Cbr-unc-119(+)*]] II; *unc-119(ed3)* III	(*33*)	Suppl. Fig. 1
MSB990	*mec-4(u253)*, *lite-1(ce314)* X; *ljIs123*(*mec-4*p::ChR2(H134R)::+*unc-122*p::RFP)	this work, from by AQ2334 x GN693	Suppl. Fig. 2
MSB1137	*mec-4(mir85(mec-4*::Active aptazyme))	this work, (*34*)	Fig. 1, Suppl. Fig. 3
MSB1244	*mirIs97* [15XUAS::ACR1::*let-858* 3'UTR **oxTi677* [*eft-3*p::tdTomato::H2B::*unc-54* 3'UTR + *Cbr-unc-119(+)*]] II; *unc-119(ed3)* III; *mirEx238*[*mec-4*p::cGAL4+*myo-2*p:mCherry]	this work	Suppl. Fig. 1
MSB1390	*srf-3(mir104)* IV	this work	Fig. 9 & Supp. Fig. 10, 11
MSB1410	*syIs423*[15xUAS::Δ*pes-10*p::GCaMP6s::SL2::mKate2::*let-858* 3'UTR + *myo-2*p::NLS::mCherry] V; *mec-4(u253)* X; *mirIs19*[*nlp-12*p::GAL-4; *unc-122*p::mCherry]	This study	Fig. 4, Suppl. Fig. 5
MSB1464	*mec-4(u253), mec-10(mir110)* X	this study	Fig. 1
MSB1487	*qxIs722* [*dpy-7*p::*dpy-7*-SfGFP]; *mec-4(mir50[mScarlet(11)::mec-4]*, *lite-1(ce314)* X; *mirIs93*[*mec-4*p::mScarlet1-10; *unc-122*p::GFP]	this study & (*35*)	Fig. 7, 8
MSB1492	*he317*[*eft-3*p-lox2272NLS::BFP::*let-858* 3' UTRlox2272-NLS::mCherry] IV; *mirEx622*[*flp-8*p::N-CRE::*tbb-2* 3'UTR+*twk-49*p::C-CRE::*tbb-2* 3'UTR+*unc-122*::gfp]	this work	parent strain to get MSB1501
MSB1499	*he317*[*eft-3*p-lox2272NLS::BFP::*let-858* 3' UTRlox2272-NLS::mCherry] IV; *uIs31(mec-17*p::GFP; *mirEx622*[*flp-8*p::N-CRE::*tbb-2* 3'UTR+*twk-49*p::C-CRE::*tbb-2* 3'UTR+*unc-122*::gfp]	this work	parent strain to generate MSB1501
MSB1500	*srf-3(mir104)* IV; *qxIs722* [*dpy-7*p::*dpy-7*-SfGFP]	this work	Fig. 9
MSB1501	*he317*[*eft-3*p-lox2272NLS::BFP::l*et-858* 3' UTRlox2272-NLS::mCherry] IV; *mirIs252*[*flp-8*p::N-CRE::*tbb-2* 3'UTR+*twk-49*p::C-CRE::*tbb-2* 3'UTR+*unc-122*::gfp]	this work	Fig. 7
MSB1505	*syIs423* [15xUAS::Δ*pes-10*::GCaMP6s::SL2::mKate2::*let-858* 3'UTR + *myo-2*p::NLS::mCherry] V; *mirIs19*[*nlp-12*p::GAL-4+*unc-122*p::mCherry] X; *mirEx627*[*mec-4*p::mScarlet::SL2::TeTx::*let-858* 3' UTR; *myo-2*p::GFP]	this work	to generate MSB1524
MSB1509	*mec-4(mir50*[mScarlet(11)::*mec-4*] *mir117*[loxP intron1], *lite-1(ce314)* X; *mirIs93*(*mec-4*p::mScarlet1-10; *unc-122*p::GFP)	this work	parent strain to generate MSB1514
MSB1514	*mec-4(mir50*[mScarlet(11)::*mec-4*] *mir117*[loxP intron1] *mir118*[loxP intron14]), *lite-1(ce314)* X; *mirIs93*(*mec-4*p::mScarlet1-10; *unc-122*p::GFP)	this work	Fig. 7

MSB1524	*syIs423* [15xUAS::Δ*pes-10*::GCaMP6s::SL2::mKate2::*let-858* 3'UTR + *myo-2*p::NLS::mCherry] V; *mirIs19*[*nlp-12*p::GAL-4+*unc-122*p::mCherry] X;*mirIs256*[*mec-4*p::mScarlet::SL2::TeTx::*let-858* 3' UTR; *myo-2*p::GFP]	this work	Fig. 4, Suppl. Fig. 5
MSB1527	*mec-4(mir50*[mScarlet(11)::*mec-4*] *mir117*[loxP intron1] *mir118*[loxP intron14]), *lite-1(ce314)* X; *mirIs93*(*mec-4*p::mScarlet1-10; *unc-122*p::GFP), *mirIs252*[*flp-8*p::N-CRE::*tbb-2* 3'UTR+*twk-49*p::C-CRE::*tbb-2* 3'UTR+*unc-122*::gfp]	this work	Fig. 7
MSB1528	*lite-1(ce314)* X; *mirIs93*(*mec-4*p::mScarlet1-10; *unc-122*p::GFP), *mirIs252*[*flp-8*p::N-CRE::*tbb-2* 3'UTR+*twk-49*p::C-CRE::*tbb-2* 3'UTR+*unc-122*::gfp]	this work	Fig. 7
MSB1538	*mec-4(mir50*[mScarlet(11)::*mec-4*]), *lite-1(ce314)* X; *mirIs93*(*mec-4*p::mScarlet1-10; *unc-122*p::GFP), *mirIs252*[*flp-8*p::N-CRE::*tbb-2* 3'UTR+*twk-49*p::C-CRE::*tbb-2* 3'UTR+*unc-122*::gfp]	this work	Fig.7
MSB1542	*otIs669* V; *lite-1(ce314)* X; *otIs672*; *mirEx638*[*mec-4*p::mScarlet::SL2::TeTx::*let-858* 3' UTR; *myo-2*p::GFP]	this work	Fig. 4, Suppl. Fig. 5
MSB1549	*dpy-10(o)* II; *mec-4(mir50(mScarlet(11)*::*mec-4*)+*mir127*(*mec-4*::Active aptazyme) *lite-1(ce314)* X; *mirIs93*(*mec-4*p::mScarlet1-10; *unc-122*p::GFP),	this study	Fig 1, Suppl. Fig. 3
OH15000	*pha-1(e2123)* III; *otIs669* V; *otIs672* [*rab-3*p::NLS::GCaMP6s + *arrd-4*p:NLS:::GCaMP6s]	(*5*)	used to generate MSB636 & MSB661
PS7201	*syIs421*[15xUAS::Δ*pes-10*p::TeTx::*let-858* 3'UTR + *myo-2*p::NLS::GFP + pBlueScript]	(*36*)	Suppl. Fig. 1
SV2049	*he317*[*eft-3*p-lox2272NLS::BFP::*let-858* 3' UTRlox2272-NLS::mCherry] IV	(*37*)	background color switch to monitor CRE recombination
TU253	*mec-4(u253)* X	(*38*)	Fig. 1, 8, Suppl. Fig. 1, 10
TU2769	*uIs31(mec-17*p::GFP)	(*39*)	used to build MSB1499
XW18042	*qxIs722*[*dpy-7*p::*dpy-7*-sfGFP]	(*35*)	for generating MSB1487 and MSB1500

Supplementary Table 3

Table 3. Worm locomotion data: Number of plates, assay days, animals analyzed from the locomotion experiments.

strain	genotype	plates	replicates	worms
	basic *mec* locomotion assay			
N2	wild-type	16	12	284
TU253	*mec-4(u253)*	9	8	151
CB75	*mec-2(e75)*	3	3	141
CB1611	*mec-4(e1611)*	6	3	80
MSB26	*mec-12(e1607)*	4	2	55
MSB1464	*mec-4(e253);mec-10(mir110)*	8	5	116
	Tetracycline-dependent conditional rescue			
MSB1137	*mec-4(mir85[mec-4*p::Active aptazyme])	5	5	63
+2.5 μM	"	5	5	70
+5 μM	"	5	5	63
+10 μM	"	5	5	71
0 μM	wild-type	4	4	51
+5 μM	"	1	1	17
+10 μM	"	2	2	29
	TRNs killing with ph-miniSOG			
CZ23281 +hν	*mec-4*p::PH::miniSOG(Q103L)	2	2	39
CZ23281 (dark)	*mec-4*p::PH::miniSOG(Q103L)	2	2	47
N2 +hν	wild-type	2	2	43
	Disruption of synaptic transmission with tetanus toxin			
PS7201	15xUAS::Δ*pes-10*p::TeTx	1	2	44
MSB683	*mec-4*p::cGAL4+15xUAS::Δ*pes-10*p::TeTx	1	1	37
	Optogenetic silencing using ACR1			
MSB1244 + ATR	*mec-4*p::cGAL4+15xUAS::ACR1	11	4	29
ctrl + ATR	wild-type	4	4	29
	Random optogenetic activation of TRNs			
MSB990 + ATR	*mec-4(u253)*; *mec-4*p::ChR2	6	6	73
MSB990 + ATR + hν	*mec-4(u253)*; *mec-4*p::ChR2	6	6	87
	Eigenworms analysis			
N2, 0.5kPa	wild-type	n.a.	3	25
N2, 3kPa	"	n.a.	3	22
N2, 100kPa	"	n.a.	2	14
TU253, 0.5kPa	*mec-4(u253)*	n.a.	3	22
TU253, 3kPa	"	n.a.	2	22
TU253, 100kPa	"	n.a.	2	21
	PVM::*mec-4* deletion			
N2	wild-type	2	2	26
MSB1528	*mec-4*p::mScarlet1-10+PVM::split CRE	1	1	17
MSB1514	mScarlet::floxed *mec-4*	1	1	15
MSB1538	mScarlet::*mec-4*+PVM:split CRE	5	2	75
MSB1527	mScarlet::floxed *mec-4*+PVM::split CRE	1	1	30

Supplementary Table 4

Ca^{2+} dynamics in the TRNs freely moving worms

strain	neuron	1.25 kPa	6 kPa	30 kPa
GN692	PLM	16/162	7/92	13/187
GN693		17/171	10/114	12/171
GN692	PVM	17/65	7/39	7/118
GN693		14/48	10/15	12/103
GN692	ALM	11/81	5/28	10/133
GN693		10/73	7/64	9/62
GN692	AVM	4/18	5/13	8/45
GN693		4/8	5/39	7/27

Table 4. Number of worms/points used covariance and cross-covariance plots. Overall 163 neurons from 158 different worms, 7 different experimental days, each recording lasted 2 min.

Supplementary Table 5

Traction Force Microscopy

strain	1.25 kPa	6 kPa	30 kPa
N2	8	8	6
TU253	9	6	8
MSB1390	9	7	7

Table 5. Number of traction force experiments performed in the different conditions.

Supplementary References

1. Kunert, J., Shlizerman, E., Kutz, J. N., Low-dimensional functionality of complex network dynamics: Neurosensory integration in the *Caenorhabditis elegans* connectome. *Physical Review E - Statistical, Nonlinear, and Soft Matter Physics* **89**, 1–6 (2014).

2. Kunert, J. M., Maia, P. D., Kutz, J. N., Functionality and robustness of injured connectomic dynamics in *C. elegans*: Linking behavioral deficits to neural circuit damage. *PLoS Computational Biology* **13**, 1–21 (2017).

3. Varshney, L. R., Chen, B. L., Paniagua, E., Hall, D. H., Chklovskii, D. B., Structural properties of the *Caenorhabditis elegans* neuronal network. *PLoS Computational Biology* **7** (2011).

4. Chalfie, M., et al., The neural circuit for touch sensitivity in caenorhabditis elegans. *The Journal of neuroscience* **5**, 956–964 (1985).

5. Yemini, E., et al., Neuropal: A multicolor atlas for whole-brain neuronal identification in *C. elegans*. *Cell* **184**, 272–288.e11 (2020).

6. Stephens, G. J., Johnson-Kerner, B., Bialek, W., Ryu, W. S., Dimensionality and dynamics in the behavior of *C. elegans*. *PLoS Computational Biology* **4** (2008).

7. Wicks, S. R., Rankin, C. H., The integration of antagonistic reflexes revealed by laser ablation of identified neurons determines habituation kinetics of the caenorhabditis elegans tap withdrawal response. *Journal of comparative physiology. A, Sensory, neural, and behavioral physiology* **179**, 675–85 (1996).

8. Simo, J. C., A finite strain beam formulation. the three-dimensional dynamic problem. part i. *Computer Methods in Applied Mechanics and Engineering* **49**, 55–70 (1985).

9. Gray, J., Hancock, G. J., The propulsion of sea-urchin spermatozoa. *Journal Experimental Biology* **32**, 802–814 (1955).

10. Maladen, R. D., Ding, Y., Li, C., Goldman, D. I., Undulatory swimming in sand: Subsurface locomotion of the sandfish lizard. *Science* **325**, 310–314 (2009).

11. Keaveny, E. E., Brown, A. E., Predicting path from undulations for *C. elegans* using linear and nonlinear resistive force theory. *Physical Biology* **14** (2017).

12. Bijalwan, A., Muñoz, J. J., Adjoint-based optimal control of contractile elastic bodies. application to limbless locomotion on frictional substrates. *Computer Methods in Applied Mechanics and Engineering* **420** (2024).

13. Lenhart, S., Workmann, J., *Optimal Control Applied to Biological Models*. Chapman Hall/CRC (2007).

14. Sharp, J. A., Burrage, K., Simpson, M. J., Implementation and acceleration of optimal control for systems biology. *Journal of the Royal Society Interface* **18** (2021).

15. Wang, X., Luo, Y., Accurate solution of a mode-superposition inverse problem based on deep learning and particle swarm optimization. *Proceedings of the 2024 2nd International Conference on Advances in Artificial Intelligence and Applications*, 59–65, ACM (2024).

16. Olivesi, C., Muñoz, J. J., Inverse analysis for the computation of growth and boundary conditions in elastic bodies. *Computational Mechanics* (2024).

17. Armengou-Riera, N., Rabiei, N., Bijalwan, A., Rodríguez-Ferran, A., Muñoz, J. J., On the convergence of conjugate gradient and gmres algorithms in the forward backward sweep method for optimal control. *Optimal Control Applications and Methods* (2026).

18. Bijalwan, A., Muñoz, J. J., A control hamiltonian-preserving discretisation for optimal control. *Multibody System Dynamics* **59**, 19–43 (2023).

19. Sznitman, J., Purohit, P. K., Krajacic, P., Lamitina, T., Arratia, P. E., Material properties of *Caenorhabditis elegans* swimming at low reynolds number. *Biophysical Journal* **98**, 617–626 (2010).

20. Chalfie, M., Sulston, J., Developmental genetics of the mechanosensory neurons of *Caenorhabditis elegans*. *Developmental Biology* **82**, 358–370 (1981).

21. Chatzigeorgiou, M., et al., Specific roles for deg/enac and trp channels in touch and thermosensation in *C. elegans* nociceptors. *Nature neuroscience* **13**, 861–8 (2010).

22. Nekimken, A. L., et al., Pneumatic stimulation of c. elegans mechanoreceptor neurons in a microfluidic trap. *Lab Chip* **17**, 1116–1127 (2017).

23. Schafer, W. R., Mechanosensory molecules and circuits in c. elegans. *Pflugers Archiv European Journal of Physiology* **467**, 39–48 (2014).

24. Fang-Yen, C., Alkema, M. J., Samuel, A. D., Illuminating neural circuits and behaviour in caenorhabditis elegans with optogenetics. *Philos Trans R Soc Lond B Biol Sci* **370** (2015).

25. Li, T., Kumar, S., Poon, H., Leifer, A. M., Zheng, C., Functional probing of neuronal subtypes via intersectional expression of optogenetic actuators reveals non-linear components in a linear circuit. *Cell Reports* **44** (2025).

26. Stirman, J. N., et al., Real-time multimodal optical control of neurons and muscles in freely behaving caenorhabditis elegans. *Nature Methods* 1–7 (2011).

27. Huang, M., Gu, G., Ferguson, E. L., Chalfie, M., A stomatin-like protein necessary for mechanosensation in *C. elegans*. *Nature* **378**, 292–295 (1995).

28. Driscol, M., Chalfie, M., The mec-4 gene is a member of a family of *Caenorhabditis elegans* genes that can mutate to induce neuronal degeneration. *Nature* **349**, 58859–593 (1991).

29. Xu, T., et al., The expanding toolbox of in vivo bioluminescent imaging. *Frontiers in Oncology* **6**, 1–8 (2016).

30. Liu, J., et al., *C. elegans* phototransduction requires a g protein-dependent cGMP pathway and a taste receptor homolog. *Nat Neurosci* **13**, 715–722 (2010), nULL.

31. Das, R., et al., An asymmetric mechanical code ciphers curvature-dependent proprioceptor activity. *Science Advances* **7**, 1–20 (2021).

32. Sanfeliu-Cerdán, N., et al., A mec-2/stomatin condensate liquid-to-solid phase transition controls neuronal mechanotransduction during touch sensing. *Nature Cell Biology* **25**, 1590–1599 (2023).

33. Malaiwong, N., Porta-de-la Riva, M., Krieg, M., Flint: single shot safe harbor transgene integration via fluorescent landmark interference. *G3 (Bethesda, Md.)* **13**, 1–12 (2023).

34. Wurmthaler, L. A., Sack, M., Gense, K., Hartig, J. S., Gamerdinger, M., A tetracycline-dependent ribozyme switch allows conditional induction of gene expression in *Caenorhabditis elegans*. *Nature Communications* **10**, 1–8 (2019).

35. Miao, R., Li, M., Zhang, Q., Yang, C., Wang, X., An ecm-to-nucleus signaling pathway activates lysosomes for *C. elegans* larval development. *Developmental Cell* **52**, 21–37.e5 (2020).

36. Yang, W., Yuste, R., In vivo imaging of neural activity. *Nature Methods* **176**, 139–148 (2017).

37. van der Vaart, A., Godfrey, M., Portegijs, V., Dose-dependent functions of swi/snf baf in permitting and inhibiting cell proliferation in vivo. *Science Advances* (2020).

38. Chalfie, M., Au, M., Genetic control of differentiation of the caenorhabditis elegans touch receptor neurons. *Science (New York, N.Y.)* **243**, 1027–33 (1989).

39. O'Hagan, R., Chalfie, M., Goodman, M. B., The mec-4 deg/enac channel of *Caenorhabditis elegans* touch receptor neurons transduces mechanical signals. *Nature Neuroscience* **8**, 43–50 (2005).
