# Supplementary material for: Mechanosensory encoding of surface mechanics optimizes locomotion
Source: Nat Commun. 2026 Jul 25;17:9083. doi: 10.1038/s41467-026-75352-7 (PMC13518797; doi:10.1038/s41467-026-75352-7)
Supplement: Supplementary file 3 [file 41467_2026_75352_MOESM2_ESM.pdf]

nature portfolio

Peer Review File

Active sensing of surface mechanics optimizes undulatory locomotion

Corresponding Author: Professor Michael Krieg

This file contains all reviewer reports in order by version, followed by all author rebuttals in order by version.

Version 0:

Reviewer comments:

Reviewer #1

(Remarks to the Author)
This manuscript presents a novel and well-supported framework for understanding how C. elegans touch receptor neurons (TRNs), especially PVM, contribute to optimizing locomotion by actively sensing substrate friction. Using an impressive array of approaches (calcium imaging, optogenetics, microfluidics, traction force microscopy, and a connectome-based computational model) the authors argue that these neurons are not merely passive detectors of mechanical contact but dynamic sensors of frictional context.

I believe that this study expands our view of mechanosensory function and suggests intriguing parallels with friction sensing in other systems.

The main contribution this work generates is the redefinition of TRNs as friction sensors that actively modulate gait dynamics. This is both novel and important, and bridges mechanosensation and motor control in a way that has not been shown before in C. elegans.

The work also combines in vivo imaging and perturbation with computational modeling, which strengthens their conclusions and raises new questions about how substrate properties are integrated into locomotor output.

The study is likely to be of interest not only to C. elegans neurobiologists but also to researchers studying sensorimotor integration, adaptive behavior, and biomechanics more broadly.

It is precisely because of the importance of this study and its findings that I feel some work is merited by the authors to improve on the delivery which I felt was challenging. I had an easy time with the text, but the figures proved an entirely different matter.

Areas that (I feel) need addressing:

1) Ecological context
The manuscript would benefit from more explicit discussion of the naturalistic relevance of the findings. C. elegans did not evolve on agar pads, but rather navigating complex 3D matrices like rotting vegetation and soil microstructures. The authors could contextualize the relevance of friction sensing and substrate compliance within this ecological framework. For example, how might the frictional properties of soil particulates or biofilms engage this sensory system in ways not captured on flat agar?

2) Figures are overly dense/non-standard
Many figures contain too many data elements or non-standard visual encodings, making interpretation challenging. The figure captions are detailed, but much of the explanation should be transferred into the figure panels themselves—especially to aid readers from adjacent fields. Some plots (e.g., violin or heatmaps with non-intuitive color maps or lack of scales) are difficult to evaluate without re-reading the caption several times. I suggest simplifying each figure’s layout, increasing font sizes, and providing more clear visual grouping or narrative flow across panels. I mean, I am active in this very field and a fun of this effort, if I struggled to work through the figures someone in an adjacent field might give up or walk away missing key insights.

3) Modeling needs additional detail
The connectome-based modeling is compelling but under-described. While the model is stated to produce emergent oscillations, it is unclear what type of neuron model was used, how motor output was quantified, or how validation was performed. A formal summary of the model architecture, parameters, and outputs—perhaps in the supplemental information—would be helpful for reproducibility and to evaluate its mechanistic plausibility.

4) Interpretation could overreach in a few instances
While the experimental evidence supports the central claims well, some statements about PVM's sufficiency or specificity in controlling gait may be stronger than the data warrants. For example, the roles of PLM, ALM, and other TRNs could be contrasted more clearly, particularly given their overlapping circuits and partially redundant functions.

5) Methodology
The methodology overall is strong and well-conceived. However, a few experimental details (e.g., animal developmental stages, statistical effect sizes) could be more thoroughly reported. The conditional mec-4 rescue system is especially interesting, but details about rescue timing and expression verification should be provided to ensure interpretability and reproducibility.

Overall I found that this is a thoughtful and well-executed study that advances our understanding of sensorimotor integration and adds a new dimension to the role of gentle touch neurons in C. elegans. With modest clarifications, improvements to figure readability, and expansion of the ecological and modeling context, the manuscript will be of high value to the field.

PS: I could not access any of the codes in GitLab as the links required me to have a login ID and Password.

Reviewer #2

(Remarks to the Author)
This manuscript explores how C. elegans employs touch receptor neurons (TRNs), specifically those expressing the MEC-4 DEG/ENaC ion channel, to detect and respond to substrate friction during locomotion. Using a sophisticated combination of calcium imaging in freely moving animals, traction force microscopy, microfluidics, and computational modeling based on the whole connectome, the authors show that MEC-4 activity correlates with crawling velocity and traction force. They further suggest a functional coupling between surface mechanosensation and proprioceptive feedback. These findings offer new insights into the biomechanical and neural integration underlying gait control in C. elegans and suggest that TRNs, especially PVM, may play an active role in substrate sensing beyond their classical touch function.

Major comments:
1. The trajectories under 10 µm tetracycline do not show stronger rescue than under 1 µm in Fig. 1h, t, contradicting the authors' claim of dose-dependent rescue. Please clarify this inconsistency with additional quantification and statistical comparison if available.

2. The functional conclusions drawn from mec-4 mutants would be strengthened by TRN-specific expression or rescue experiments.

3. The PVM calcium signal appears to correlate positively with backward locomotion velocity, while PVM is considered to promote forward movement. Please address this contradiction, ideally with phase-aligned analyses around transition points or optogenetic validation.

Minor comments:

1. The term "Ho" in Fig. 1e needs to be clearly defined—what does it stand for, and where, or how is it measured?

2. The ALM neuron appears to be missing from the model in Fig. 2a, why? Please clarify the reason for this omission and discuss whether its absence could affect the validity of the simulation outcomes.

3. There are labeling errors, missing scale bars, and missing labels indicating "before" and "after" stimulation in Fig. 3a (ii and iii). Please revise for accuracy and completeness.

4. Please provide stacked kymographs of PDE activity in mec-4 mutants in Fig. 3c, in addition to the wild type, to support your comparisons.

5. DVA responses post-stimulation show heterogeneity, with both increases and decreases in calcium activity, including in mec-4 mutants Fig. 3f,g. Please provide average $\Delta F/F_0$ values and statistical comparisons between wild type and mec-4.

6. These panels would benefit from direct side-by-side comparative plots (e.g., bar graphs with significance testing) to facilitate interpretation for Fig. 5e, 5i, 5k.

7. Please include representative body curvature plots for both wild-type and mec-4 mutants on substrates of different stiffness in Extended Fig. 3a, b, along with all raw curvature data to support downstream quantifications.

Reviewer #3

(Remarks to the Author)
Aleksandra et al. investigate the role of body wall gentle touch receptor neurons (TRNs) in C. elegans locomotion, hypothesizing TRNs act as mechanosensors detecting environmental mechanical load to drive movement. While the hypothesis is moderately interesting, the results align with prior literature and the study lacks significant field advancement.

Specific concerns:
The claims “where this difference is strongly reduced off food while males retain their mobile activity even on food” and “this phenotype is not due to a defect in muscle function or general paralysis” require presentation of control data for validation.

TRN::CHR experiments should be reclassified as Extended Data Fig. S2a-e (not Fig. 2a-e). The unlabeled time scale in Extended Data Fig. S2a hinders data interpretation. The conclusion “MEC-4 does not induce changes in plasticity” based on optogenetic stimulation of mec-4 mutants is questionable, as results may depend on stimulation patterns and lack causal linkage.

The manuscript suffers from a significant number of errors and confusions regarding the figures. These include issues like duplicate figure pasting and chaotic figure labeling, which not only disrupt the reading flow but also raise concerns about the overall quality and rigor of the study:
Figs. 1a/b/c-d (TRN::minoSOG, TTX, AC experiments) should be relabeled as Extended Data Fig. 1a/b/c-d.
Fig. 3 lacks sample size (n) information, undermining statistical clarity.
Extended Data Fig. 8 duplicates Extended Data Fig. 7 (incorrect data).
Extended Data Fig. 9 has ambiguous labeling (a/b/c axes unclear: kPa or genotypes?).
Extended Data Fig. 10c is mislabeled (should be Fig. 10b).

The calcium imaging data in Fig. 3 is insufficient to support the claim that TRN-to-proprioceptor input loss causes locomotion deficits, given prior studies on PDE and DVA mechanosensitivity.

The manuscript requires extensive revisions to clarify data presentation, strengthen conclusions with rigorous analysis, and correct figure/writing errors to enhance scientific rigor and readability.

Reviewer #4

(Remarks to the Author)
Active sensing of surface friction optimizes undulatory locomotion.

The authors report a number of locomotory, neurophysiological, and mechanical measurements suggesting that mec-4 and its associated proprioceptive neurons show a “new role” in C. elegans locomotion, specifically in the active measurement and modification of “friction.”

While I appreciate the wide number of experiments and data shown in this paper, I only see suggested results and no proven results. In addition I have a number of problems with the modeling and I am also confused in the authors' interpretation of friction and the resulting traction forces.

First off we can start with the title. Even after reading the paper it is not clear what is meant by “active sensing.” In addition it is not clear what is being optimized.

My main concern is that the resistive force theory (RFT) model here is very naive. While this model is useful for swimming worms in bulk liquid. Crawling worms on agar or similar gels is more complicated. A number of papers show this, but none have been referenced. For example:

https://www.cell.com/biophysj/abstract/S0006-3495(12)00565-6

https://pmc.ncbi.nlm.nih.gov/articles/PMC4213666/

C. elegans is surrounded by a thin film of liquid and these lubrication and adhesion forces cause deformation of the agar surface (depending on stiffness) by the body of C. elegans forming a “groove.” The modeling of forces and friction is difficult due to this complexity.

Comments:

Main Text: “...we propose that the gentle body wall mechanoreceptors are friction sensors”

This sentence illustrates one problem the authors have when discussion friction and traction (or forces). It seems that they interchange these terms often throughout the paper and even in the figures. For example in Figure 5, the legend states “Traction map showing the uneven distribution of friction exerted on the substrate” Has friction actually been calculated from the traction measurement? If so it is not at all clear how this was done.

Results: “This suggests that MEC-4 does not induce changes in plasticity, or mec-4-induced changes in plasticity cannot be rescued using entrainment protocols.” This statement goes beyond what the experiments show. For all we know it could take many days of mec-4 dependent stimulation in a development dependent manner for changes in basal locomotory activity.

Results: “To provide direct evidence that MEC-4 function enhances vivid locomotion,” It is not clear what is meant by “vivid” locomotion. Are we not just talking about “normal” or “wild-type” locomotion?

Results: Halo-carbon oil swimming experiment. It is not clear the purpose of this experiment. Are you saying that the mechanoreceptors are not capable of sensing forces due to viscous drag and somehow only sensitive to visco-elastic forces? Are the experiments done in a similar force range?

Results: SRF-3 loss of function experiments. Much more evidence of wild-type locomotion is needed here. The reference is a Journal of Biological Chemistry paper and deals mostly with the chemical composition of the glycoconjugates.

Discussion: “Wild type worms seem able to modulate these two conditions efficiently by sensing and adapting their friction.” How are the worms modulating their friction? Nowhere is a mechanism proposed.

Version 1:

Reviewer comments:

Reviewer #2

(Remarks to the Author)
The authors have adequately addressed my comments and made substantial, valuable revisions to the manuscript. While I acknowledge the concerns regarding the predictability of the paper’s novelty raised by other reviewers, given the rigorous experimental validation presented in this work, I recommend acceptance of the manuscript.

Reviewer #3

(Remarks to the Author)
The revised manuscript is improved and addresses several of my previous concerns. The authors have corrected a number of figure and labeling errors, and moderated some of the conclusions that were previously overstated. I also appreciate the inclusion of additional experiments, particularly the new calcium imaging analyses, which strengthen the evidence for a functional link between TRNs and downstream neurons. I remain of the view that some of the mechanistic conclusions are supported only indirectly. In particular, the data are consistent with a role for TRN input in shaping proprioceptive output, but do not yet fully establish that loss of TRN-to-proprioceptor signaling is the direct cause of the locomotion defects. Overall, the revision is improved and substantially clearer, but careful wording of the main conclusions remains important.

Reviewer #4

(Remarks to the Author)
The authors have done a tremendous amount of work in response to the wide ranging comments of the reviewers. After reviewing all the changes, it is clear to me that some of my issues came down to semantics and the degree of certainty in interpretation of results. I'm generally happy with the changes made in response to my specific comments.

Open Access This Peer Review File is licensed under a Creative Commons Attribution 4.0 International License, which permits use, sharing, adaptation, distribution and reproduction in any medium or format, as long as you give appropriate credit to the original author(s) and the source, provide a link to the Creative Commons license, and indicate if changes were made.
In cases where reviewers are anonymous, credit should be given to 'Anonymous Referee' and the source.
The images or other third party material in this Peer Review File are included in the article's Creative Commons license, unless indicated otherwise in a credit line to the material. If material is not included in the article's Creative Commons license and your intended use is not permitted by statutory regulation or exceeds the permitted use, you will need to obtain permission directly from the copyright holder.
To view a copy of this license, visit https://creativecommons.org/licenses/by/4.0/

Active sensing of surface mechanics optimizes undulatory locomotion

We thank the reviewers for their constructive feedback. In response, we have performed a series of new experiments and analyses to address the points raised and to strengthen the manuscript. These include targeted genetic manipulations to selectively delete the *mec-4* gene exclusively in PVM neurons via an intersectional CRE/lox strategy, optogenetic experiments with Poisson-distributed stimuli to address whether random, spontaneous activity induces plasticity effects, and microfluidic assays to probe TRN-proprioceptor coupling. We also performed RT-PCR to validate tet-on ribozyme expression, analyzed locomotion across multiple mec alleles and conditions (including off-food and male animals), and conducted additional calcium imaging on PVM neurons on elastic substrates. Additional controls include independent replicates for the tet-on system, tetracycline dose-response tests, verification of *mec-4* gene expression upon tetracyclin treatment and *srf-3* mutant characterization (locomotion, cuticle staining, touch sensitivity). Mechanistic insights were further supported by examining correlations between *mec-4* and *dpy-7*, highlighting cuticle ridges as potential sites of force transfer. Finally, we improved the statistical analysis throughout the text, and updated the supplementary information regarding the modeling details. Collectively, we believe that these improvements and additions as suggested by the referees substantially reinforce the conclusions of the presented results.

1 Reviewer #1 (Remarks to the Author):

This manuscript presents a novel and well-supported framework for understanding how C. elegans touch receptor neurons (TRNs), especially PVM, contribute to optimizing locomotion by actively sensing substrate friction. Using an impressive array of approaches (calcium imaging, optogenetics, microfluidics, traction force microscopy, and a connectome-based computational model) the authors argue that these neurons are not merely passive detectors of mechanical contact but dynamic sensors of frictional context.

I believe that this study expands our view of mechanosensory function and suggests intriguing parallels with friction sensing in other systems.

The main contribution this work generates is the redefinition of TRNs as friction sensors that actively modulate gait dynamics. This is both novel and important, and bridges mechanosensation and motor control in a way that has not been shown before in C. elegans.

The work also combines in vivo imaging and perturbation with computational modeling, which strengthens their conclusions and raises new questions about how substrate properties are integrated into locomotor output.

The study is likely to be of interest not only to C. elegans neurobiologists but also to researchers studying sensorimotor integration, adaptive behavior, and biomechanics more broadly.

It is precisely because of the importance of this study and its findings that I feel some work is merited by the authors to improve on the delivery which I felt was challenging. I had an easy time with the text, but the figures proved an entirely different matter.

Areas that (I feel) need addressing:

1. Ecological context The manuscript would benefit from more explicit discussion of the naturalistic

relevance of the findings. C. elegans did not evolve on agar pads, but rather navigating complex 3D matrices like rotting vegetation and soil microstructures. The authors could contextualize the relevance of friction sensing and substrate compliance within this ecological framework. For example, how might the frictional properties of soil particulates or biofilms engage this sensory system in ways not captured on flat agar?

We appreciate the reviewer's insightful comment regarding the ecological relevance of our findings. While our experiments were performed on agar substrates for technical consistency and control, *C. elegans* obviously evolved in complex, heterogeneous environments such as rotting vegetation and soil, where frictional interactions are highly variable and spatially structured.

In response, we have added a new paragraph to the Discussion section that contextualizes friction sensing within the worm's natural habitat. We highlight how substrate roughness, compliance, and particulate interactions–such as those found in soil, bacterial mats, or decomposing plant material-likely impose a range of mechanical challenges that could engage this sensory system in diverse ways. We also discuss how the ability to detect frictional cues may aid in navigation through irregular terrain, anchoring during turns, or modulating gait in response to environmental stiffness.

In particular we write: "While our experiments were conducted on uniform agar substrates, *C. elegans* naturally inhabits highly heterogeneous environments such as decomposing plant matter, biofilms, and soil micropores[1]. These settings present spatially variable friction, compliance, and adhesion. Friction sensing may therefore not only modulate gait on smooth surfaces but also enable the worm to detect and adapt to local terrain properties in three-dimensional microenvironments. For example, increased friction could facilitate anchoring during high-force turns or burrowing, whereas low-friction regions might trigger adjustments in posture or direction to maintain propulsion. Although agar assays do not capture the full complexity of these environments, our findings provide a mechanistic basis for how frictional feedback could shape locomotion in natural substrates[2]."

2. Figures are overly dense/non-standard Many figures contain too many data elements or non-standard visual encodings, making interpretation challenging. The figure captions are detailed, but much of the explanation should be transferred into the figure panels themselves–especially to aid readers from adjacent fields.

   We thank the referee for their comment on the figure. We have now relaxed the content and re-evaluated the information density. To do so, we replaced some of the 2D-statistical information with estimation statistics and compared animal crawling velocity as a metric for lethargic phenotypes. This reduces the 2D angular and vectorial (forward/backward) velocity plots to an easier accessible 1D distribution visualized as a violin plot. In addition, we have transferred the important information from the caption into the panels whenever possible without overcrowding the figure display.

   Some plots (e.g., violin or heatmaps with non-intuitive color maps or lack of scales) are difficult to evaluate without re-reading the caption several times. I suggest simplifying each figure's layout, increasing font sizes, and providing more clear visual grouping or narrative flow across panels. I

mean, I am active in this very field and a fun of this effort, if I struggled to work through the figures someone in an adjacent field might give up or walk away missing key insights.

Please see our response to this point above.

3. Modeling needs additional detail The connectome-based modeling is compelling but under-described. While the model is stated to produce emergent oscillations, it is unclear what type of neuron model was used, how motor output was quantified, or how validation was performed. A formal summary of the model architecture, parameters, and outputs–perhaps in the supplemental information–would be helpful for reproducibility and to evaluate its mechanistic plausibility.

   We agree with the referee that the model warrants more explicit information and apologize for not having introduced this better before. The model is based on a recent publication (Kunert, Maia and Kutz, Functionality and Robustness of Injured Connectomic Dynamics in C. elegans: Linking Behavioral Deficits to Neural Circuit Damage, PLOS Comp. Biology, 2017; [3]) which we adapted to model the effect of the presence and absence of individual neurons on spontaneous crawling behavior. This model uses a single compartment membrane model (Kunert J, Schlizerman E, Kutz JN, Low-dimensional functionality of complex network dynamics: Neuro- sensory integration in the Caenorhabditis elegans connectome. Phys Rev E. 2014; [4]), which model the full somatic connectome with neurons as passive linear units that are connected by linear gap junctions and nonlinear chemical synapses. As this connectome model lacks the incorporation of mechanical elements such as muscles contraction, we used the singular value decomposition of the ensuing motor neuron activity as a readout for muscle function. We observed that under normal conditions, motorneuron response is oscillatory and forms a limit cycle in the reduced phase space, indicative for rhythmic locomotion activity. We also observed that this rhythmic activity is altered in situations in which touch receptor neurons are decoupled from the network. The effect of different TRNs on motorneuron dynamics was different, as ALM and AVM ablation lead to a reduction in activity while PVM ablation lead to increased motor neuron activity. This warrants the hypothesis that TRN activity in general and PVM activity in particular may be required for proper locomotion activity and modulates the motor neuron output through synaptic interaction with PDE and DVA. We have now added a more detailed description of the model, its parameters and outputs in the supplementary material. We have tested this prediction and our hypothesis specifically in a microfluidic experiment, delivering mechanical forces to the body wall of immobilized animals in which TRN synaptic transmission was disrupted through expression of a tetanus toxin. We found that both, DVA and PDE responded less to body wall touch in the TRN::TeTx expressing animals than their control counterparts.

4. Interpretation could overreach in a few instances While the experimental evidence supports the central claims well, some statements about PVM's sufficiency or specificity in controlling gait may be stronger than the data warrants. For example, the roles of PLM, ALM, and other TRNs could be contrasted more clearly, particularly given their overlapping circuits and partially redundant functions.

   We agree with the referee and thank her/him for motivating us to dissect this response in greater detail. To directly address the question whether or not PVM has a role in locomotion and friction sensing, we have selectively deleted *mec-4* exclusively in PVM using a conditional CRE/lox recom-

bination strategy. In short, we combined two constructs that express a split CRE recombinase under the *flp-8* and *twk-49* promotors, respectively. The two promotors intersect with MEC-4 expression only in PVM and therefore results in a highly specific, cell-exclusive knock out of MEC-4 in a single TRN [5]. We proved the excision using a CRE-dependent color switch as described in Das et al, Science Advances 2021; [6], and confirmed nearly 100% reconstitution in PVM. We then generated a loxP:MEC-4::mScarlet11:loxP strain carrying a mScarlet1-10 complementation transgene resulting in functional fluorescence of the host protein allowing us to follow MEC-4 expression. This fusion, importantly, did not result in a defective channel, as touch-evoked behavior remained unchanged compared to control animals. Finally, we then expressed the split CRE together with the floxed MEC-4, verified loss of fluorescence in PVM and recorded locomotion videos. Importantly, these animals are touch sensitive and comparable to wildtype animals. We found that animals expressing CRE recombinase in PVM specifically and hence lack functional MEC-4 in PVM were lethargic - animals that only expressed PVM:CRE were not. This data supports the hypothesis that PVM is may have unrecognized roles in locomotion. We have included this data as a new Figure 5.

5. Methodology The methodology overall is strong and well-conceived. However, a few experimental details (e.g., animal developmental stages, statistical effect sizes) could be more thoroughly reported. We have now added the missing experimental details into the methods and statistical information into the appropriate subsection and figure legends throughout the manuscript.

   The conditional mec-4 rescue system is especially interesting, but details about rescue timing and expression verification should be provided to ensure interpretability and reproducibility.
   We have now repeated these experiments and added additional detail to the methods. We have also verified that the introduction of the ribozyme-aptamer (aptazyme) leads to a loss of expression of MEC-4 protein, whereas the addition of tetracycline blocked the ribozyme function leading to reexpression of the MEC-4 protein. In order to follow this and retain functionality of the MEC-4 protein, we leveraged a split mScarlet strategy as described above [7]. To do so, we introduced the 11th strand of the fluorescent protein mScarlet on the C-terminus of MEC-4 at its endogenous genomic locus using CRISPR/Cas9, co-expressed the 1-10 fragment under the *mec-4* promotor and verified that its localization is indistinguishable to the literature. We then introduced the aptazyme into the 3' UTR of this transgenic locus and found that this effectively abolished MEC-4 expression to a level that is undetectable by fluorescence microscopy, whereas the addition of 10μM tetracycline rescued protein expression as determined by fluorescence microscopy. In addition, we investigated the expression of *mec-4* cDNA in absence and presence of tetracycline. We performed a series of semiquantitative RT-PCR experiments and found that concentrations above 5μM tetracycline added to the plates robustly and reproducibly increased *mec-4* cDNA. We have added this information to the Supplementary Figure S1. To further confirm that the addition of tetracycline itself does not conform with a change in locomotion, we added the appropriate amounts to wildtype worms and measured the speed (Supplementary Figure S1). Importantly, we did not observe a significant change in speed in lower concentrations of 5μM and a subtle increase of animals in presence of 10μM tetracyline.

Overall I found that this is a thoughtful and well-executed study that advances our understanding of

sensorimotor integration and adds a new dimension to the role of gentle touch neurons in *C. elegans*. With modest clarifications, improvements to figure readability, and expansion of the ecological and modeling context, the manuscript will be of high value to the field.

We deeply appreciate the reviewers comments and thank him/her for their insightful ideas and suggestions for improvement.

PS: I could not access any of the codes in GitLab as the links required me to have a login ID and Password.

2 Reviewer #2 (Remarks to the Author):

This manuscript explores how *C. elegans* employs touch receptor neurons (TRNs), specifically those expressing the MEC-4 DEG/ENaC ion channel, to detect and respond to substrate friction during locomotion. Using a sophisticated combination of calcium imaging in freely moving animals, traction force microscopy, microfluidics, and computational modeling based on the whole connectome, the authors show that MEC-4 activity correlates with crawling velocity and traction force. They further suggest a functional coupling between surface mechanosensation and proprioceptive feedback. These findings offer new insights into the biomechanical and neural integration underlying gait control in *C. elegans* and suggest that TRNs, especially PVM, may play an active role in substrate sensing beyond their classical touch function.

2.1 Major comments:

1. The trajectories under 10 µM tetracycline do not show stronger rescue than under 1 µM in Fig. 1h, t, contradicting the authors' claim of dose-dependent rescue. Please clarify this inconsistency with additional quantification and statistical comparison if available.
   We thank the referee for pointing this out. We agree, the dose-response was overstated and we have toned down our claim and we have now conducted additional experiments and analyses and compared the results statistically. Indeed, we observed a rescue of locomotion behavior already at 2.5 µM tetracycline concentration which only marginally increased with higher and was not statistically significant between 5 and 10 µM. We should point out though that a dose-response was observed in the touch test, where a doubling of the tetracycline concentration lead to a statistically significant increase in the touch response. We have appropriately modeled the binary response probability using a mixed-effects logistic regression model. In our mixed-effects logistic regression, individual animals were included as random intercepts to account for repeated measurements (multiple touches) within each animal. The estimated random intercept standard deviation (SD = 0.89) reflects substantial animal-to-animal variability in baseline responsiveness that is independent of tetracycline dose. Specifically, this value indicates that the baseline log-odds of responding at 0 µM tetracycline vary across animals by approximately ± 0.89 around the population mean, corresponding to a broad range of baseline response probabilities. Including this random effect ensures that inter-animal heterogeneity is appropriately modeled and prevents inflation of the fixed-effect estimate for dose. We have clarified this interpretation in the Results section. In addition to these behavioral tests, we have verified transgene re-expression upon supplementation of tetracycline (which inhibits the ribozyme activity and leads to

functional mRNA translation).

2. The functional conclusions drawn from mec-4 mutants would be strengthened by TRN-specific expression or rescue experiments.

   MEC-4 reporter constructs are exclusively expressed in TRNs, as evidenced by us and others (e.g. Hong, Nature, 1994). We therefore believe that expressing a TRN-specific rescue construct does not add any additional evidence to the results presented here and in the literature that MEC-4 acts TRN autonomously. Instead, we have extensively verified the the re-expression of mec-4 mRNA in our spatiotemporal gene expression control using the tet-on ribozyme.

3. The PVM calcium signal appears to correlate positively with backward locomotion velocity, while PVM is considered to promote forward movement. Please address this contradiction, ideally with phase-aligned analyses around transition points or optogenetic validation.

   We thank the reviewer for the opportunity to clarify the interpretation of the cross-covariance analysis shown in Figure 4e i.

   We first want take advantage to clarify the 'classical' role of the different TRNs in driving mechanosensory avoidance. Laser killing experiments carried out on individually ablated neurons has found that anterior neurons ALML/R and AVM are mediating a reversal escape response when animals are stimulated on the anterior body segments, PLML/R have been shown to be crucial for acceleration when animals are stimulated on the posterior body part. In contrast killing PVM has little consequences on touch-evoked behavior [8], despite PVM being mechanoresponse to body touch (Nekimken, LoC, 2017 [9]; Chatzigerogiou, JNeurophys, 2010 [10]). Thus, the consensus in the literature still is that PVM is not necessary and not sufficient to evoke mechanosensory behavior. In particular, in Chalfie, J Neuroscience, 1985 [8] we learned: "Ablation of PVM has no detectable effect upon touch sensitivity. Killing all the microtubule cells, or all except PVM, results in animals that are touch insensitive over their entire length, except at the very front of the head." Recent reviews from the Schafer and Fang-Yen lab and additional optogenetic activations corroborate that a functional role for PVM has not yet been demonstrated. In addition, a recent investigation aiming to decipher the role of individual TRNs on behavior suggested that PVM has a modulatory, rather than a driving role in the touch response (PMID: 26240427, PMID: 40975868). At present, we therefore do not have enough evidence to assume that PVM promotes escape behavior.

   The reviewer asked to perform optogenetic validations, but PVM-specific expression of channelrhodopsin was not possible to date by us and others [11].

   That being said, we agree with the referee that the calcium dynamics showed a complex and context dependent behavior which deserves a better explanation, especially since this is the first in depth study of how PVM activity relates to animal movement.

   The plots in Figure 4 display the distribution of cross-covariance values between calcium activity in the sensory neuron and locomotion velocity across lags (τ). Cross-covariance was computed for each recording trace, and the distributions for control and *mec-4* animals are shown as density maps (center) with their respective marginal distributions projected onto the top and right axes.

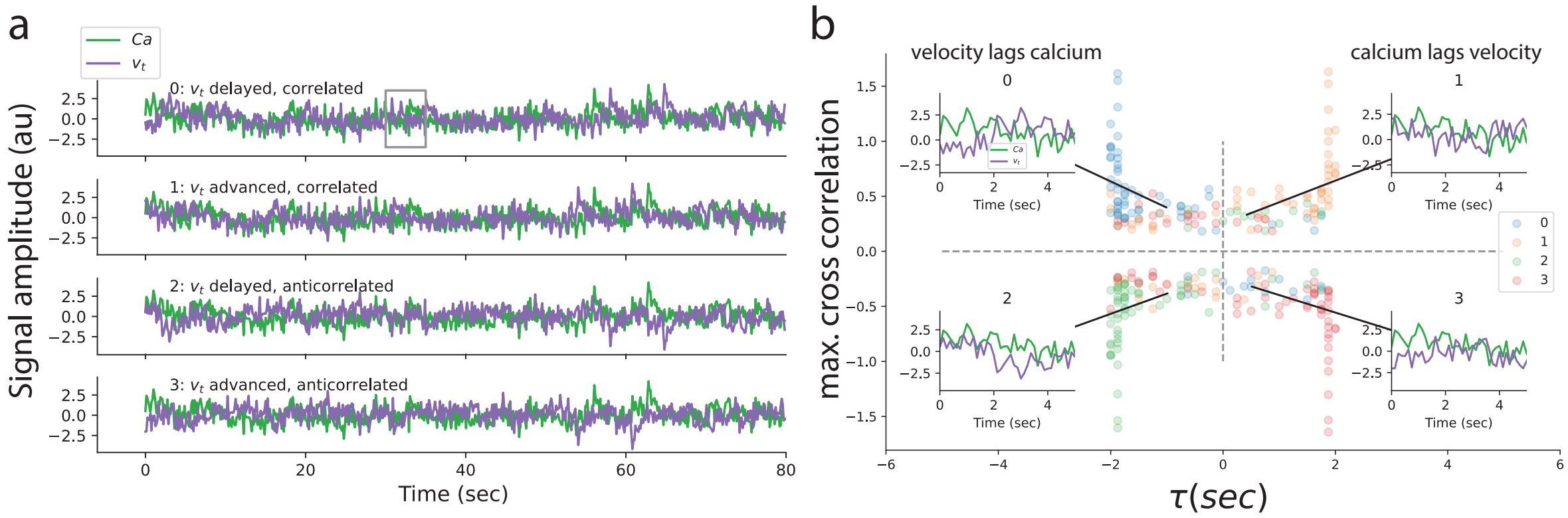

Fig. 1: Calculation of the cross-covariance from the worm velocity and calcium activity. **a,** Four different time-varying signals representing correlated and anticorrelated calcium activity and animal velocity. **b,** Corresponding calculation of their cross-covariance showing which trace maps to which quadrant.

Lags are defined such that positive τ values indicate velocity leading calcium, and correspond to sensory encoding, where changes in velocity precede and are followed by changes in neuronal calcium. In contrast, negative τ values indicate calcium leading velocity and the left quadrants reflect motor command generation, during which TRN calcium signals precede behavioral reversals or deceleration. For further clarification, we simulated the cross-covariance response from two correlated, noisy timeseries (Fig. R1).

Indeed, we observed a *mec-4* dependent correlation in which calcium activity preceded an increase in velocity (left quadrant in Fig 4e) and also an increase after a peak in crawling velocity (right quadrant in Fig 4e).

The density of covariance values near $\tau = 0$ in PVM indicates a tight temporal coupling between sensory neuron activity and ongoing locomotion. Whereas control animals show a more pronounced and consistent covariance peak at negative and positive lag, *mec-4* mutants generally exhibit a broader and less coherent distribution, suggesting reduced or more variable coupling when mechanotransduction is impaired.

The covariance structure therefore reflects a combination of sensory encoding (velocity $\rightarrow$ Ca; $\tau > 0$) and motor command generation (Ca $\rightarrow$ velocity change; $\tau < 0$). Thus, the recorded neuron encodes both aspects of the animal's movement, responding to velocity and to drive reversals when activated.

In control animals, the sharp covariance structure suggests:

- Reliable sensory encoding: changes in velocity are consistently followed by changes in Ca at short poitive lags.
- Effective motor output: increases in Ca often precede behavioral change in locomotion at short negative lags.

In *mec-4* mutants, the flat covariance patterns indicate an impaired mechanosensory drive: the neuron's calcium signal tracks velocity less reliably. It also suggests a weaker motor influence: calcium events do not translate into consistent reversal initiation or velocity changes.

The overall reduction in cross-covariance amplitude and temporal precision in *mec-4* animals is consistent with a loss of mechanosensory input through the MEC-4 channel, affecting both how movement is encoded and how sensory activation is converted into behavioral output.

As displayed in the Supplementary Fig. 4 (and underlined by the simulations in Referee Fig above), the individual quadrants can be explained by the following:

(a) The positive lag of the positive cross-covariance indicates that forward movement triggers calcium activity. This is consistent with our hypothesis that PVM is activated by velocity-dependent sensing of substrate mechanics (e.g. traction-dependent activity).

(b) The negative lag of the positive covariance indicates that calcium activity triggers forward movement. This is consistent with the idea that PVM causes accelerations similar to other posterior touch receptor neurons such as PLM.

(c) The negative lag of the negative covariance indicates that calcium activity in PVM triggers backward locomotion. This is inconsistent with the idea of the standard touch response.

(d) The positive lag of the negative covariance indicates that the backward locomotion triggers a PVM calcium transient. This is consistent with the hypothesis that PVM is activated by velocity-dependent substrate sensing (e.g. traction-dependent activity).

Because of the high covariance in quadrant 1, it gives first direct hints that PVM has a role on substrate and velocity sensation as studied in detail in this paper. The high covariance in Q2 also suggests that PVM may drive acceleration and reversals. Together, we put the idea forward that PVM has a dual context dependent role as a substrate mechanosensor to adjust proprioception and motor output.

We have expanded on this in the main text and added this detailed explanation as a supplementary discussion into the Supplementary Text and refer the interested reader to this part of the manuscript.

2.2 Minor comments:

1. The term “Ho” in Fig. 1e needs to be clearly defined–what does it stand for, and where, or how is it measured?
   We have omitted the panels with the 2D statistics to simplify the presentation as suggested by the referees.

2. The ALM neuron appears to be missing from the model in Fig. 2a, why? Please clarify the reason for this omission and discuss whether its absence could affect the validity of the simulation outcomes.
   Thanks for catching this. The model contains ALM and all other neurons and their described connections. We have omitted ALM from the sketch for clarity and convenience as only PVM and PLM make connections to the proprioceptive neurons. Attached is an updated schematic for the information of the referee, but this does not affect the model. As a matter of fact, ALM is also plotted in Figure 2e, showing the fold-change in activity of all neurons following the removal of PVM input ($I_{\mathrm{PVM}} = 0$). Intriguingly, when we performed the simulations after removing ALM from the connectome, the motorneuron output is also severely affected and reduced, leading to a fixed point in the

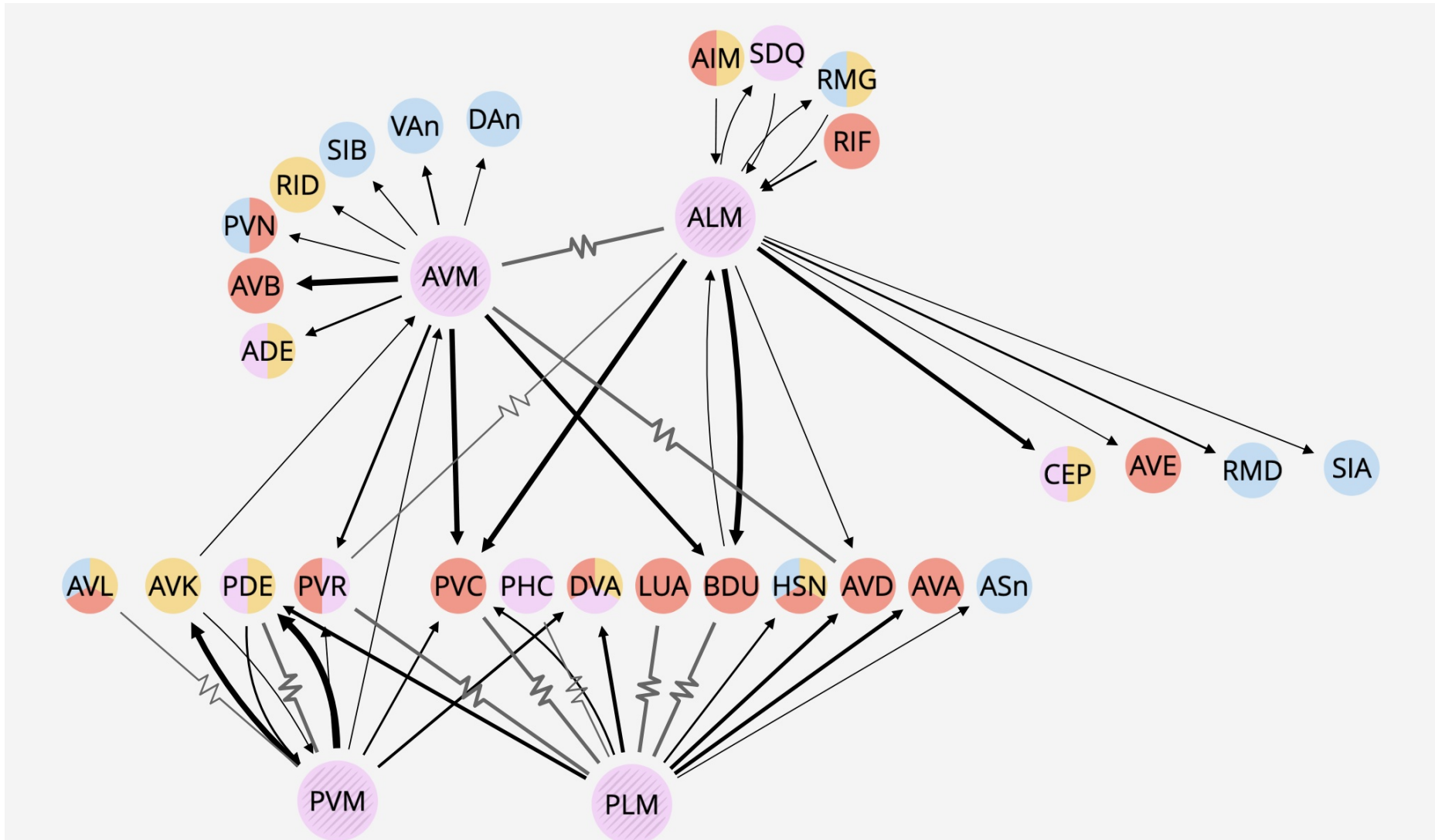

Fig. 2: Depiction of the TRN connectome including posterior and anterior touch receptors. Only posterior neurons are coupled to proprioceptive PDE and DVA neurons. Adapted from nemanode.org

principle component subspace (Suppl. Fig. S2). We have commented this in the text but prefer to keep the simplified figure. We have also added a detailed section into the Supplementary Discussion with the relevant details of the model.

3. There are labeling errors, missing scale bars, and missing labels indicating "before" and "after" stimulation in Fig. 3a (ii and iii). Please revise for accuracy and completeness.

   Thank you, we have fixed these labels.

4. Please provide stacked kymographs of PDE activity in mec-4 mutants in Fig. 3c, in addition to the wild type, to support your comparisons.
   Thank you for this suggestion. We have now repeated and expanded the dataset and included the stacked kymographs for all conditions tested, including TeTx manipulations in TRNs. In short, both mec-4 mutations and TRNs neurotransmission affected that measured PDE activity, indicating that gentle touch response modulates proprioceptive signaling.

5. DVA responses post-stimulation show heterogeneity, with both increases and decreases in calcium activity, including in mec-4 mutants Fig. 3f,g. Please provide average $\Delta F/F_0$ values and statistical comparisons between wild type and mec-4.
   Thank you for pointing this out. Due to the variability and context dependent response of DVA to the mechanical stimulus, we have initially decided to avoid averaging individual recordings and rather display individual responses in the stacked kymographs. However, we agree with the reviewer that this average response may still be helpful to some of our readers and determine a collective response of DVA. We included this analysis as Extended Data Fig 4, together with the statistical comparison of the two averages. We performed the same analyses with PDE and the mec-4::TeTx transgenic animals. To do so, we performed a pairwise comparison and calculated a p-value at each given time point in the timeseries, as described before ([12, 13, 14]. In short we write in the methods: "After arithmetic averaging all recordings, the t-test statistics for each timepoint in the timeseries was calculated according to $t = \frac{\mu_1 - \mu_0}{s_1/\sqrt{n_1} + s_0/\sqrt{n_0}}$ where μ_1 and μ_0 are the mean values of each group at time t,

s_1 and s_0 are the standard deviations for each group at time t and n equals the number of recording for each group. The t-test statistics was then used to calculate the p-value considering the degrees of freedom $n_1 + n_0 - 2$ and the null hypothesis was rejected if the absolute value of t was larger than its critical value at the level of significance α=0.01. All operations were performed in R using custom routines."

6. These panels would benefit from direct side-by-side comparative plots (e.g., bar graphs with significance testing) to facilitate interpretation for Fig. 5e, 5i, 5k.

   We appreciate the reviewer's suggestion to include direct side-by-side comparisons. However, the quantities shown in these panels are intrinsically two-dimensional distributions, and reducing them to one-dimensional bar plots would obscure the key features (e.g., the joint structure, asymmetry, and covariance) that are central to the biological interpretation. For this reason, a bar-graph summary would not faithfully represent the underlying data.

   To facilitate comparison while preserving the full structure, we have instead added marginal projections of the traction force along the y-axis, which allow direct visual and quantitative comparison between conditions without collapsing the data in a way that risks misinterpretation. We hope this addresses the reviewer's goal of improving clarity while maintaining the integrity of the multidimensional dataset.

   To enable direct comparison between conditions while respecting the intrinsic two-dimensional nature of the data, we also included a violin plot summarizing the distribution of average tractions at a given body coordinate (Fig. 6n). To appropriately test for differences, we employed a linear mixed-effects model with strain as a fixed effect and body coordinate as both a fixed and random effect to account for repeated measurements along the body. This model captures both the systematic influence of strain and the intra-worm correlation of tractions along the body, allowing us to assess the main effects of strain, body position, and their interaction while appropriately handling the nested structure of the data. F-statistics and p-values from this model are reported in the figure legend and the main text.

7. Please include representative body curvature plots for both wild-type and mec-4 mutants on substrates of different stiffness in Extended Fig. 3a, b, along with all raw curvature data to support downstream quantifications.

   We have now added representative examples to these figures and the raw curvature data can be found on zenodo.org under 10.5281/zenodo.18153808.

3 Reviewer #3 (Remarks to the Author):

Aleksandra et al. investigate the role of body wall gentle touch receptor neurons (TRNs) in C. elegans locomotion, hypothesizing TRNs act as mechanosensors detecting environmental mechanical load to drive movement. While the hypothesis is moderately interesting, the results align with prior literature and the study lacks significant field advancement.

3.1 Specific concerns:

1. The claims “where this difference is strongly reduced off food while males retain their mobile activity even on food” and “this phenotype is not due to a defect in muscle function or general paralysis” require presentation of control data for validation.

   mec-4 mutant animals are perfectly able to crawl when stimulated using light or other sensory stimuli, despite being insensitive to gentle body touch and being lethargic. This has been published multiple times and we now refer the reader to these earlier results [15, 16]. Even animals lacking TRNs are not paralyzed but completely capable of moving when stimulated otherwise. From Husson et al [17]: ”Interestingly, *mec-4(d)* mutants lacking TRNs responded with accelerations when we mechanically stimulated the tail (likely due to PVD activation) and reversed when anteriorly poked (Figure 4D). This may be explained by the action of additional mechanosensory neurons; possible candidates are the branched FLP neurons that envelop the anterior region and mainly synapse to AVA.”. We have cited this paper in the main text of the manuscript.

   In addition we have repeated experiments in N2, *mec-2* and *mec-4* males and show that they are not lethargic and even move more than N2 hermaphrodites. We have added this quantification to the Extended Data Fig. 1, and a statement in the main text. This confirms what is known from the literature and indicates that TRNs have a context-dependent and sexually dimorphic role in locomotion.

2. TRN::CHR experiments should be reclassified as Extended Data Fig. S2a-e (not Fig. 2a-e). The unlabeled time scale in Extended Data Fig. S2a hinders data interpretation.

   We apologize for the confusion and thank the referee for pointing towards this mistake, we have corrected this in the new version.

   The conclusion “MEC-4 does not induce changes in plasticity” based on optogenetic stimulation of mec-4 mutants is questionable, as results may depend on stimulation patterns and lack causal linkage.

   We have now rephrased the conclusion more conservatively and interpreted that random induced activity in TRNs in absence of MEC-4 does not alleviate the lethargic phenotype. In particular we write: “Together, our findings indicate that the entrainment protocols used here are insufficient to explain plasticity-related phenotypes in the absence of MEC-4. However, we cannot rule out the possibility that MEC-4-dependent plasticity requires longer or developmentally timed stimulation protocols.”

3. The manuscript suffers from a significant number of errors and confusions regarding the figures. These include issues like duplicate figure pasting and chaotic figure labeling, which not only disrupt the reading flow but also raise concerns about the overall quality and rigor of the study:

   - Figs. 1a/b/c-d (TRN::minoSOG, TTX, AC experiments) should be relabeled as Extended Data Fig. 1a/b/c-d.
     Done
   - Fig. 3 lacks sample size (n) information, undermining statistical clarity. We have added the sample size of tested animals to the figure legend. We have also conducted statistical analysis and added this into Extended Data Figure 4.

- Extended Data Fig. 8 duplicates Extended Data Fig. 7 (incorrect data).
  We sincerely apologize for this error, we have now corrected this and provided the correct figure.
- Extended Data Fig. 9 has ambiguous labeling (a/b/c axes unclear: kPa or genotypes?).
  We have now indicated the categorical variable next to the panel labels. In short, a, b and c show the tractions (a stress that has units in Pa) measured on different elastic substrates (that have also units Pa for their elasticity) for three different genotypes (i-iii).
- Extended Data Fig. 10c is mislabeled (should be Fig. 10b).
  Done

4. The calcium imaging data in Fig. 3 is insufficient to support the claim that TRN-to-proprioceptor input loss causes locomotion deficits, given prior studies on PDE and DVA mechanosensitivity.
   We thank the reviewer for their insightful comment. We agree that previous studies show that DVA and PDE are mechanosensitive, and that our original data were insufficient to definitively link TRN input loss to locomotion defects. We have therefore moderated our conclusions to a predictive statement.

   To address this, we performed additional calcium imaging experiments in mec-4 mutants and in animals with TRN neurotransmission blocked via tetanus toxin. We found that:

   - In mec-4 mutants, PDE and DVA responses to TRN mechanical stimulation were strongly reduced, suggesting that PDE and DVA activity depends on TRN input.
   - In TRN-silenced animals, DVA responses were altered: deactivation events decreased while activation events increased, leading to an overall increase in net DVA activity.

   These results are consistent with prior reports that DVA mechanosensitivity influences locomotion [6] and support a role for TRN input in regulating proprioceptive output. Regarding PDE, although its cell-autonomous mechanosensitivity has not been fully characterized, our data indicate that PDE receives synaptic input from TRNs, as mec-4 mutation abolishes PDE responses despite the absence of mec-4 expression in PDE.

5. The manuscript requires extensive revisions to clarify data presentation, strengthen conclusions with rigorous analysis, and correct figure/writing errors to enhance scientific rigor and readability.
   We thank the referee for her/his time to critically assess the manuscript. We hope that the referee appreciates the additional experiments and analyses that we put into it, with the aim to enhance the scientific rigor and readability.

4 Reviewer #4 (Remarks to the Author):

Active sensing of surface friction optimizes undulatory locomotion.

The authors report a number of locomotory, neurophysiological, and mechanical measurements suggesting that mec-4 and its associated proprioceptive neurons show a "new role" in C. elegans locomotion, specifically in the active measurement and modification of "friction."

While I appreciate the wide number of experiments and data shown in this paper, I only see suggested results and no proven results.

We thank the reviewer for their appreciation and the critical comment. We have now added substantial more data to underline our claims and test our hypotheses. In addition, we phrased conclusions more conservatively, and clearly differentiate between experimental results and their interpretation. To our knowledge, our experiments and results are new to the field and provide new insight into the role of substrate mechanosensation during locomotion. In addition, we added new data to strengthen the conclusions, performed new analyses to ground the comparisons in statistical rigor and related the results to previous literature. Among the new experiments we performed:

- Performed new calcium imaging in PVM on elastic substrate to ensure reproducibility, obtain more replicates to understand the influence on animal and behavioral variability and perform appropriate copmarisons

- We repeated the spontaneous locomotion experiment with a set of mutants and conditions to perturb TRN function, such as more mutant alleles involving *mec-12*, *mec-10* and *mec-4*, but also interfered with synaptic transmission using a tetanus toxin. Lastly we performed careful acute inhibition of TRN activity using light-controlled anion channelrhodopsins that suppress calcium signaling. This data is presented in Extended Data Fig 1.

- We investigated TRN $\rightarrow$ proprioceptor signaling in the microfluidic device and visualized how PDE and DVA activity depends on TRN activity and synaptic output. We repeat these experiments in the mec-4 mutant and in an animal with defective synaptic transmission in TRNs. We observed an almost complete absence of PDE and DVA activity in the *mec-4* mutant. We also observed a strong reduction on PDE and DVA activity when synaptic signaling in TRNs is perturbed.

- We examined the spatial relationship between MEC-4, the pore-forming subunit of the mechanosensitive channel, and DPY-7, a well-established marker of cuticle furrows. Our analysis shows that MEC-4 is, on average, positioned between DPY-7–labeled furrows, indicating that the channel preferentially localizes to cuticle ridges. This spatial bias may have functional significance, as it suggests that mechanosensory input could be transmitted through the ridged regions of the cuticle.

Finally, we revised the text to reflect when talking about the interpretation, we carefully use language like ”suggest,” ”may indicate,” or ”are consistent with” but also explicitly acknowledge limitations in the discussion.

In addition I have a number of problems with the modeling and I am also confused in the authors' interpretation of friction and the resulting traction forces.

1. First off we can start with the title. Even after reading the paper it is not clear what is meant by “active sensing.” In addition it is not clear what is being optimized.

   By 'active sensing' we mean the neural sensing of substrate traction, which adjusts proprioceptive activity and entices undulatory locomotion. This mechanosensory feedback from surface mechanics overcome locomotor quiescence in C. elegans. For avoiding confusion, we have replaced 'friction' in the title by 'traction', to emphasize that the feedback is based on forces rather than friction coefficients, which are less direct to measure and sense.

By optimization of the locomotion we mean the efficiency of the undulatory movement on producing net displacement of the worm center of mass. We have now tried to measure such efficiency by computing the ratio of dissipated and bending energy, E_d/E_b. The dissipated energy is computed as $E_d = \int_0^T \int_0^L (\mu_t||v_t||^2 + \mu_n||v_n||^2) dl\ dt$ and measures the loos of energy from the normal and tangential relative velocities (v_t, v_n) with respect to the substrate, while $E_b = EI \int_0^T \int_0^L (\theta')^2 dl\ dt$ measures the active energy spent by the worm (see Methods). We have found that on *mec-4* mutants, the ratio E_d/E_b was substantially larger (see Fig. 6k), providing a proxy for efficiency.

2. My main concern is that the resistive force theory (RFT) model here is very naive. While this model is useful for swimming worms in bulk liquid. Crawling worms on agar or similar gels is more complicated. A number of papers show this, but none have been referenced. For example:

   https://www.cell.com/biophysj/abstract/S0006-3495(12)00565-6

   https://pmc.ncbi.nlm.nih.gov/articles/PMC4213666/

   C. elegans is surrounded by a thin film of liquid and these lubrication and adhesion forces cause deformation of the agar surface (depending on stiffness) by the body of C. elegans forming a "groove." The modeling of forces and friction is difficult due to this complexity.

   We recognise that our friction law is a simple one. However, adding more complex laws such as the lubrication theory (LT) cited by the reviewer would complicate exceedingly the inference of the parameters in the inverse problem. In fact, lubrication theory provides an adaptive mechanism that the worm can use for tuning the drag force. In LT, the forces per unit of length along the worm body are inversely proportional to a normalised film thickness $e = h_0/C$, with h_0 the thickness at the midline and $C = (1/2)(R_w^{-1} - R_a^{-1})R_w^2$ a shape factor that takes into account the radius of the assumed circular cross-section R_w and the radius of the film-substrate interface R_a.

   According to the expression in Shen et al., Phys. Biol. 2012 [18], as the radius R_w increases, the tangential component of friction (μ_t) increases, while the normal component (μ_n) decreases, resulting in a net decrease of the drag coefficient μ_n/μ_t. We show in Figure 3 these trends for the typical case $h_0 = 5\mu m$, $R_a = 100\mu m$, and a groove width of 28 μm (Shen et al,2012). In this manner, it is explained how the worm can modulate its friction with the substrate: modifying the radius of the circular cross-section at different points of the backbone. We note that other manners can be hypothesised, such as adopting an elliptical cross-section rather than circular, without changing the cross-section area.

   Additionally, in these references the drag ratio is close to 10, which is much closer to our values, than those previously reported as ~ 1.5 [19, 20].

   We have included one of the references and the potential strategy for modulating the drag coefficient in the Discussion section. We acknowledge the reviewer for pointing this out.

4.1 Comments:

1. Main Text: "...we propose that the gentle body wall mechanoreceptors are friction sensors" This sentence illustrates one problem the authors have when discussion friction and traction (or forces). It seems that they interchange these terms often throughout the paper and even in the figures. For

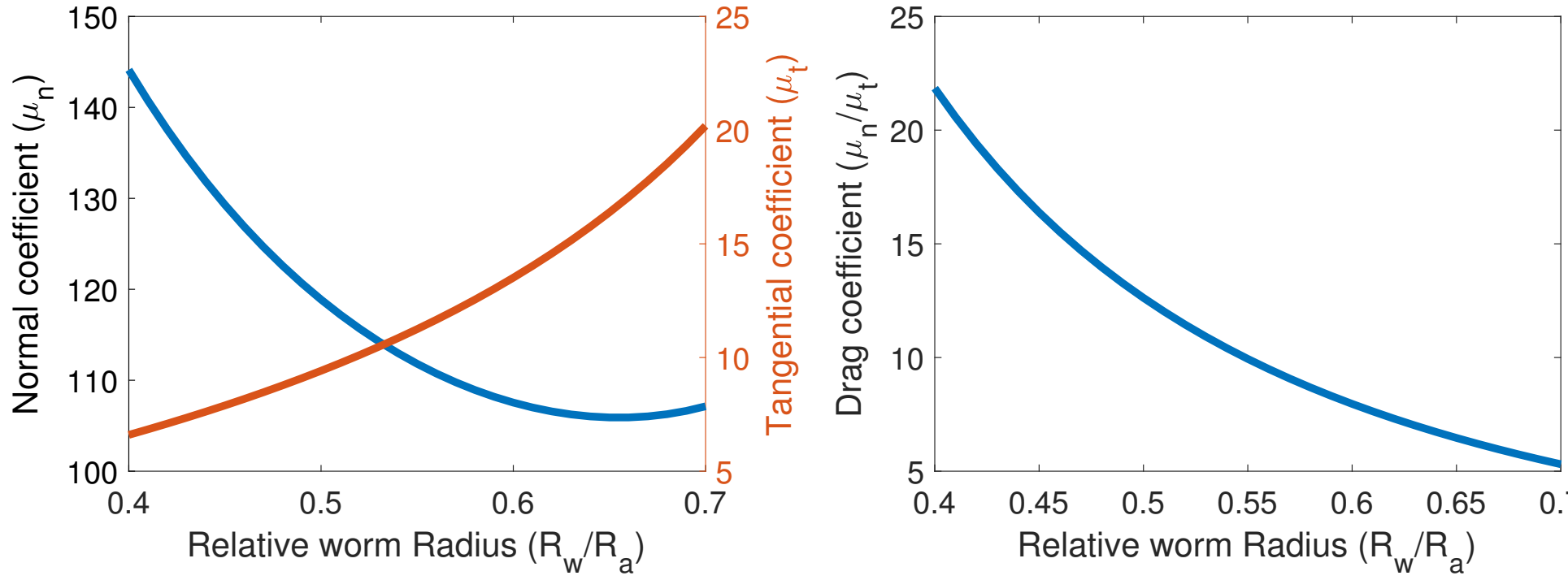

Fig. 3: Evolution of friction coefficients and Drag coefficient ratio as function of worm radius R_w for some typical values of groove radius $R_a = 100\mu m$ and central distance of worm-substrate $h_0 = 5\mu m$.

example in Figure 5, the legend states "Traction map showing the uneven distribution of friction exerted on the substrate" Has friction actually been calculated from the traction measurement? If so it is not at all clear how this was done.

Indeed, we have used the term "friction" for both, "friction forces" (tractions) and "friction coefficients", which has resulted in some confusions. We have now revised the text for consistency, and written explicitly to which term we are referring to, "friction forces", "friction tractions" (forces per unit of surface), or "friction coefficients". To answer the question of the referee, we directly calculated friction from the traction map but found the resulting maps not interpretable due to abundance of experimental noise. To assess the frictional interaction of the animals with the substrate, we therefore resorted to the mechanical model that allowed us to extract the tractions and the friction coefficient as a function of body coordinate and time from the worm kinematics. This is now explicitly stated in the text and in the new figure 6 g and h.

2. Results: "This suggests that MEC-4 does not induce changes in plasticity, or mec-4-induced changes in plasticity cannot be rescued using entrainment protocols." This statement goes beyond what the experiments show. For all we know it could take many days of mec-4 dependent stimulation in a development dependent manner for changes in basal locomotory activity.

   We thank the reviewer for this important point. We agree that the current data do not rule out the possibility that MEC-4 influences plasticity through mechanisms that require extended stimulation, specific developmental timing, or different entrainment protocols. We have revised the text to reflect this and now frame our interpretation more conservatively to avoid overstating the conclusion.

   In addition, we have extended the paradigm and tested various stimulation protocols and durations, starting from L2 animals as described in the new material methods section.

   In particular we write: "Together, our findings indicate that the entrainment protocols used here are insufficient to explain plasticity-related phenotypes in the absence of MEC-4. However, we cannot rule out the possibility that MEC-4-dependent plasticity requires longer or developmentally timed stimulation protocols."

3. Results: "To provide direct evidence that MEC-4 function enhances vivid locomotion," It is not clear what is meant by "vivid" locomotion. Are we not just talking about "normal" or "wild-type" locomo-

tion? We have rephrased this sentence and replace the word 'vivid'.

4. Results: Halo-carbon oil swimming experiment. It is not clear the purpose of this experiment. Are you saying that the mechanoreceptors are not capable of sensing forces due to viscous drag and somehow only sensitive to visco-elastic forces? Are the experiments done in a similar force range?

   We thank the referee for asking this question and motivating us to re-describe the motivation behind this assay. To recapitulate: In wild-type animals, TRN calcium activity is tightly coupled to locomotion in a bidirectional manner: negative τ peaks indicate that TRN transients precede and may drive velocity changes, while positive τ peaks reflect reafferent feedback from ongoing movement. This structure highlights the closed sensorimotor loop in which touch neurons both sense frictional forces during crawling and modulate locomotor output. In mec-4 mutants lacking functional mechanosensory channels, this correlation collapses, and TRN signals neither predict nor follow movement–consistent with the loss of mechanosensory drive. To understand if substrate friction is required for TRN activation, we asked if animal locomotion in a viscous medium produces enough drag force to elicit mechanoreceptor activity, as the drag force is transmitted through the cuticle and body wall and could, in principle, stretch or shear the membrane or cuticle at the TRN endings and open mechanotransduction channels.

   However, when animals move in viscous halocarbon oil, these correlations nearly disappear. Cross-covariance becomes weak, broad, and symmetric, with control and mec-4 traces largely overlapping. This loss of structured coupling suggests that TRNs are not strongly activated when worms locomote against distributed viscous drag. If drag were a strong, temporally-structured driver, we would expect TRN activity to show clearer coupling to the phase of bending (for example, peaks aligned to maximum curvature or to bending velocity) – but the cross-covariance shows no strong phase-locked structure. However, we would like to point out that this does not prove drag has zero effect – it only shows drag is not producing the same, strong, MEC-4–dependent sensorimotor coupling found on agar.

   As per the question if the experiments are done in the similar force range, we evaluated whether viscous drag in halocarbon oil could itself activate TRNs, and estimated the magnitude of local forces under our experimental conditions. For a worm of length $L = 1$mm and radius $a \approx 40$ µm, the drag per unit length on a slender cylinder moving at velocity v in a Newtonian fluid of viscosity η can be approximated by resistive force theory:

$$\zeta_\perp \approx 4\pi\eta \ln(L/a) + 12, \qquad \zeta_{||} \approx 2\pi\eta \ln(L/a) - 12$$

   With the viscosity of halocarbon oil (η=700 cSt $\approx$ 0.7 Pa· s), we obtain $\zeta_\perp \approx 2.36$ N· s/m^2, $\zeta_{||} \approx$ 1.62N· s/m^2. The drag per unit length is given by $f = \zeta \cdot v$. For a representative normal velocity during body bending of $v \approx 50$µm/s$\approx 5 \times 10^{-5}$m/s (as measured in Figure 4b), the transverse drag becomes $f_\perp = 2.36 \cdot 5 \cdot 10^{-5} = 1.18 \cdot 10^{-4}$N/m. Over the length of a TRN-relevant segment ($\Delta x \approx 50$µm), the total local viscous load is therefore $F_{vis} = f_\perp \cdot \Delta x = 1.18 \cdot 10^{-4}$ N/m·5010^{-6}m$\approx 6 \cdot 10^{-9}$ N. That is, approximately 6 nN of distributed drag force per 50 µm of neuronal coverage.

This value is at least an order of magnitude smaller than the $\sim$ 100-200 nN indentation forces reported to robustly activate TRNs with a glass probe [21]. Although viscous drag is larger than the piconewton scale required to gate single MEC-4 channels, the viscoelastic nature of the cuticle and the underlying tissues lead to dampening and eventual dissipation of the mechanical force.

By contrast, during crawling on agar, the forces are dominated by substrate friction. A simple expression is:

$$F_{friction} = \mu N,$$

where μ is the effective coefficient of friction between the worm body and agar, and N is the normal force (body weight per unit length). Effective friction coefficients for *C. elegans* on agar have been inferred from traction force measurements, yielding forces in the range of 10–1000 μN per animal [22, 23, 19]. On the scale of a 50 μm TRN segment, this corresponds to hundreds of nN of local load–well above the behavioral activation threshold.

Together, these estimates strongly support the interpretation that viscous drag in oil is insufficient to directly activate TRNs, whereas crawling on agar produces large frictional forces that robustly engage MEC-4–dependent mechanotransduction.

5. Results: SRF-3 loss of function experiments. Much more evidence of wild-type locomotion is needed here. The reference is a Journal of Biological Chemistry paper and deals mostly with the chemical composition of the glycoconjugates.

   We agree with the referee and have conducted a better characterization of the newly generated srf-3 loss of function. First we performed a classical antigenicity test by incubation wildtype and srf worms in fluorescently labeled wheat germ agglutinin (WGA) and found, consistent with previous reports, that wildtype animals did not stain for WGA. In contrast and as reported before, srf knockout mutants strongly bound to WGA, confirmed the absence of the surface glycocalyx. Next, we performed locomotion analysis on agar substrates and found a slight but significant reduction in crawling speed. We also found that the overall cuticle structure and organization seemed unchanged, as indicated by a 'normal' distribution of DPY-7, a marker for cuticle furrow collagens. We have summarized this data and analyses in a new Extended Data Figure and describe this in the text as follows: "Using CRISPR Cas9, we created a *srf-3* loss-of-function allele (Fig. 6l) which led to strong cuticle staining with wheat germ agglutinin (Extended Data Fig. 9a) as described before. Even though crawling speed on agar was slightly slower (Extended Data Fig. 9b), neither body shape nor cuticle structure was affected by *srf-3* loss of function, as determined by quantifying *dpy-7* distribution, a label for cuticle furrows (Extended Data Fig. 9d-g). We then tested their locomotion on PAA gels using traction force microscopy."

6. Discussion: "Wild type worms seem able to modulate these two conditions efficiently by sensing and adapting their friction." How are the worms modulating their friction? Nowhere is a mechanism proposed.

   We thank the reviewer for highlighting this important point. We agree that our original statement may

have implied an active modulation of friction without sufficient mechanistic support. We have revised the text to clarify that wild-type worms likely adapt their locomotor behavior in response to traction force, rather than directly modulating frictional properties themselves. While the exact mechanism remains to be determined, our findings suggest that sensory feedback allows them to adjust gait and posture in a manner consistent with the sensation of traction forces.

We specifically write: "Wild-type worms seem to modulate these two conditions efficiently by sensing and adapting their traction force. Some partial explanation for this modulation may be found by resorting to more sophisticated friction laws, such as lubrication theory [18], where frictional coefficients depend on the curvature of the groove and the worm cross-section. Consequently, by changing the curvature of the cross-section while bending, the worm may be able to modify the contact area and the drag coefficient ratio between normal and tangential friction coefficients. *mec-4* animals are sensory deficient and thus are not able to properly synchronize their muscle activity with their curvature profile. Intriguingly, lab cultivated strains seem to have lost the higher effective drag anisotropy, which means the wild isolates crawl with less surface slip[24]. Interestingly though, *srf-3* mutants may crawl less on all substrates assay, despite having a higher traction force. This points to a scenario where the defective glycocalyx leads to stonger adhesion/friction of the animal with the substrate such that it appears to move less."

References

[1] Schulenburg, H., Felix, M. A., The natural biotic environment of caenorhabditis elegans. *Genetics* **206**, 55–86 (2017).

[2] Wang, T., et al., Mechanical intelligence simplifies control in terrestrial limbless locomotion. *Sci. Robot* **8**, 2243 (2023).

[3] Kunert, J. M., Maia, P. D., Kutz, J. N., Functionality and robustness of injured connectomic dynamics in c. elegans: Linking behavioral deficits to neural circuit damage. *PLoS Computational Biology* **13**, 1–21 (2017).

[4] Kunert, J., Shlizerman, E., Kutz, J. N., Low-dimensional functionality of complex network dynamics: Neurosensory integration in the caenorhabditis elegans connectome. *Physical Review E - Statistical, Nonlinear, and Soft Matter Physics* **89**, 1–6 (2014).

[5] Taylor, S. R., et al., Molecular topography of an entire nervous system. *Cell* **184**, 1–19 (2021).

[6] Das, R., et al., An asymmetric mechanical code ciphers curvature-dependent proprioceptor activity. *Science Advances* **7**, 1–20 (2021).

[7] Goudeau, J., et al., Split-wrmscarlet and split-sfgfp: tools for faster, easier fluorescent labeling of endogenous proteins in caenorhabditis elegans. *Genetics* **217** (2021).

[8] Chalfie, M., et al., The neural circuit for touch sensitivity in caenorhabditis elegans. *The Journal of neuroscience : the official journal of the Society for Neuroscience* **5**, 956–964 (1985).

[9] Nekimken, A. L., et al., Pneumatic stimulation of c. elegans mechanoreceptor neurons in a microfluidic trap. *Lab Chip* **17**, 1116–1127 (2017).

[10] Chatzigeorgiou, M., et al., Specific roles for deg/enac and trp channels in touch and thermosensation in c. elegans nociceptors. *Nature neuroscience* **13**, 861–8 (2010).

[11] Li, T., Kumar, S., Poon, H., Leifer, A. M., Zheng, C., Functional probing of neuronal subtypes via intersectional expression of optogenetic actuators reveals non-linear components in a linear circuit. *Cell Reports* **44** (2025).

[12] de-la Riva, M. P., et al., Neural engineering with photons as synaptic transmitters. *Nature Methods* **20**, 761–769 (2023).

[13] Setty, H., et al., Sexually dimorphic architecture and function of a mechanosensory circuit in c. elegans. *Nature Communications* **13**, 1–36 (2022).

[14] Inberg, S., et al., Sensory experience controls dendritic structure and behavior by distinct pathways involving degenerins. *eLife* **14** (2025).

[15] Nagel, G., et al., Light activation of channelrhodopsin-2 in excitable cells of caenorhabditis elegans triggers rapid behavioral responses. *Current Biology* **15**, 2279–2284 (2005).

[16] Krieg, M., Dunn, A. R., Goodman, M. B., Mechanical control of the sense of touch by β-spectrin. *Nature Cell Biology* **16**, 224–233 (2014).

[17] Husson, S. J., et al., Optogenetic analysis of a nociceptor neuron and network reveals ion channels acting downstream of primary sensors. *Current Biology* **22**, 743–752 (2012).

[18] Shen, C., Khonsari, M. M., Effect of dimple's internal structure on hydrodynamic lubrication. *Tribology Letters* **52**, 415–430 (2013).

[19] Rabets, Y., Backholm, M., Dalnoki-Veress, K., Ryu, W. S., Direct measurements of drag forces in c. elegans crawling locomotion. *Biophysical Journal* **107**, 1980–1987 (2014).

[20] Gray, J., Lissmann, H. W., The locomotion of nematodes. *J. Exp. Biol* **41**, 3–4 (1964).

[21] O'Hagan, R., Chalfie, M., Goodman, M. B., The mec-4 deg/enac channel of caenorhabditis elegans touch receptor neurons transduces mechanical signals. *Nature Neuroscience* **8**, 43–50 (2005).

[22] Fang-Yen, C., et al., Biomechanical analysis of gait adaptation in the nematode caenorhabditis elegans. *Proc Natl Acad Sci U S A* (2010).

[23] Sznitman, J., Purohit, P. K., Krajacic, P., Lamitina, T., Arratia, P. E., Material properties of caenorhabditis elegans swimming at low reynolds number. *Biophysical Journal* **98**, 617–626 (2010).

[24] Keaveny, E. E., Brown, A. E., Predicting path from undulations for c. elegans using linear and nonlinear resistive force theory. *Physical Biology* **14** (2017).
