# Supplementary material for: Mechanosensory encoding of surface mechanics optimizes locomotion
Source: Nat Commun. 2026 Jul 25;17:9083. doi: 10.1038/s41467-026-75352-7 (PMC13518797; doi:10.1038/s41467-026-75352-7)
Supplement: Supplementary file 4 [file 41467_2026_75352_MOESM3_ESM.pdf]

Description of Additional Supplementary Files

File name: Supplementary Movie 1
Description: Representative video of N2 control, *mec-4* and *mec-2* mutant animals on NGM agar supplemented with food.

File name: Supplementary Movie 2
Description: Representative video of the crawling N2 control animal on 0.5kPA PAA hydrogel.

File name: Supplementary Movie 3
Description: Representative video of the *mec-4* mutant animal crawling on 0.5kPA PAA hydrogel.

File name: Supplementary Movie 4
Description: Representative video of control animal expressing GCaMP6s in PDE, PVM, PVD and SDQ, subjected to three consecutive mechanical stimuli anterior to the PVM cell body (left most stimulator). Posterior to the right.

File name: Supplementary Movie 5
Description: Representative video of the control animal expressing GCaMP6 in DVA, subjected to three consecutive mechanical stimuli to the PVM neurites.

File name: Supplementary Movie 6
Description: Representative calcium recording of control animals expressing GCaMP6s in TRNs crawling on 1.25 kPA PAA hydrogel.

File name: Supplementary Movie 7
Description: Representative calcium recording of *mec-4* mutant animals expressing GCaMP6s in TRNs crawling on 1.25 kPA PAA hydrogel.

File name: Supplementary Movie 8
Description: Representative recording of a traction force microscopy experiment using wild-type control animal crawling on 1.25 kPA poly-acrylamide hydrogel. Displacement is visualized using gel-embedded microspheres.

File name: Supplementary Movie 9
Description: Representative result of the model used to infer the friction coefficients, showing the locomotion of the worm on 6kPa substrate and the underlying traction components. The static dashed line is the initial body posture, thick line is the worm's backbone with inferred non-homogeneous friction and the thin blue line is the hypothetical worm's backbone with homogeneous friction coefficient.
